# Systematic Expansion of an Ugi-Based Multicomponent Synthesis of Tetrasubstituted Imidazoles

**DOI:** 10.1021/acsomega.5c13353

**Published:** 2026-03-27

**Authors:** Robin van der Straat, Justyna Kalinowska-Tluscik, Katarzyna Kurpiewska, Alexander Dömling

**Affiliations:** † Department of Medicinal Chemistry, Photopharmacology and Imaging, Groningen Research Institute of Pharmacy, University of Groningen, 9713 AV Groningen, The Netherlands; ‡ Department of Crystal Chemistry and Crystal Physics, Faculty of Chemistry, Jagiellonian University, Gronostajowa 2, 30-387 Krakow, Poland; § Innovative Chemistry Group, Czech Advanced Technology and Research Institute (CATRIN), Palacký University Olomouc, Slechtitelů 27, 77900 Olomouc, Czech Republic; ⊥ Innovative Chemistry Group, Institute of Molecular and Translational Medicine (IMTM), Faculty of Medicine and Dentistry, Palacký University and University Hospital Olomouc, 77900 Olomouc, Czech Republic

## Abstract

Highly substituted imidazoles are privileged scaffolds in medicinal and synthetic chemistry; however, general and modular access to densely substituted variants remains limited. Although Ugi-derived imidazole formation has been reported in isolated cases, its broader applicability has not been systematically explored. Herein, we present a comprehensive expansion and optimization of an Ugi-based one-pot synthesis enabling the preparation of tetrasubstituted imidazoles from readily accessible glyoxal derivatives. In contrast to earlier studies largely restricted to aryl glyoxals, this protocol demonstrates broad compatibility with aliphatic and aromatic glyoxals, as well as diverse amines, carboxylic acids, and isocyanides, providing full substitution control over all four positions of the imidazole ring. Key parameters governing chemoselectivity and ammonium-induced cyclization were identified, affording the target imidazoles in moderate to excellent yields. This study establishes the Ugi–imidazole transformation as a robust and diversity-oriented synthetic platform suitable for the rapid generation of medicinally relevant imidazole scaffolds.

## Graphical abstract

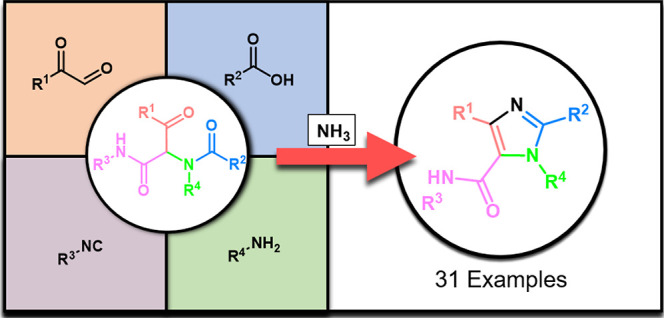

## Introduction

Imidazoles represent a privileged class of heterocycles that are ubiquitous in natural products, pharmaceuticals, and functional materials (Figure 1). The imidazole motif is found in biologically essential structures such as the amino acid histidine and is present in numerous therapeutic agents exhibiting antifungal, antibacterial, anti-inflammatory, and analgesic activities.^1–4^ Beyond medicinal chemistry, imidazoles also play important roles as ligands in organometallic catalysis and as components of ionic liquids, further underscoring their broad synthetic relevance,^5,6^ many synthetic approaches have been developed to yield differently substituted imidazoles. Owing to their importance^7^ and since its first synthesis in 1858 by Heinrich Debus,^8^ a wide range of synthetic strategies toward imidazoles has been developed. Classical approaches include the Debus–Radziszewski synthesis, cyclization of α-acylaminoketones,^9^ dehydrogenation of imidazolines,^10^ and the van Leusen three-component reaction.^11^ While these methods enable access to a variety of substituted imidazoles, many suffer from limitations such as the requirement for prefunctionalized or symmetric building blocks (e.g., benzil), restricted substitution patterns, or limited flexibility in introducing aliphatic diversity.

**Figure 1:**
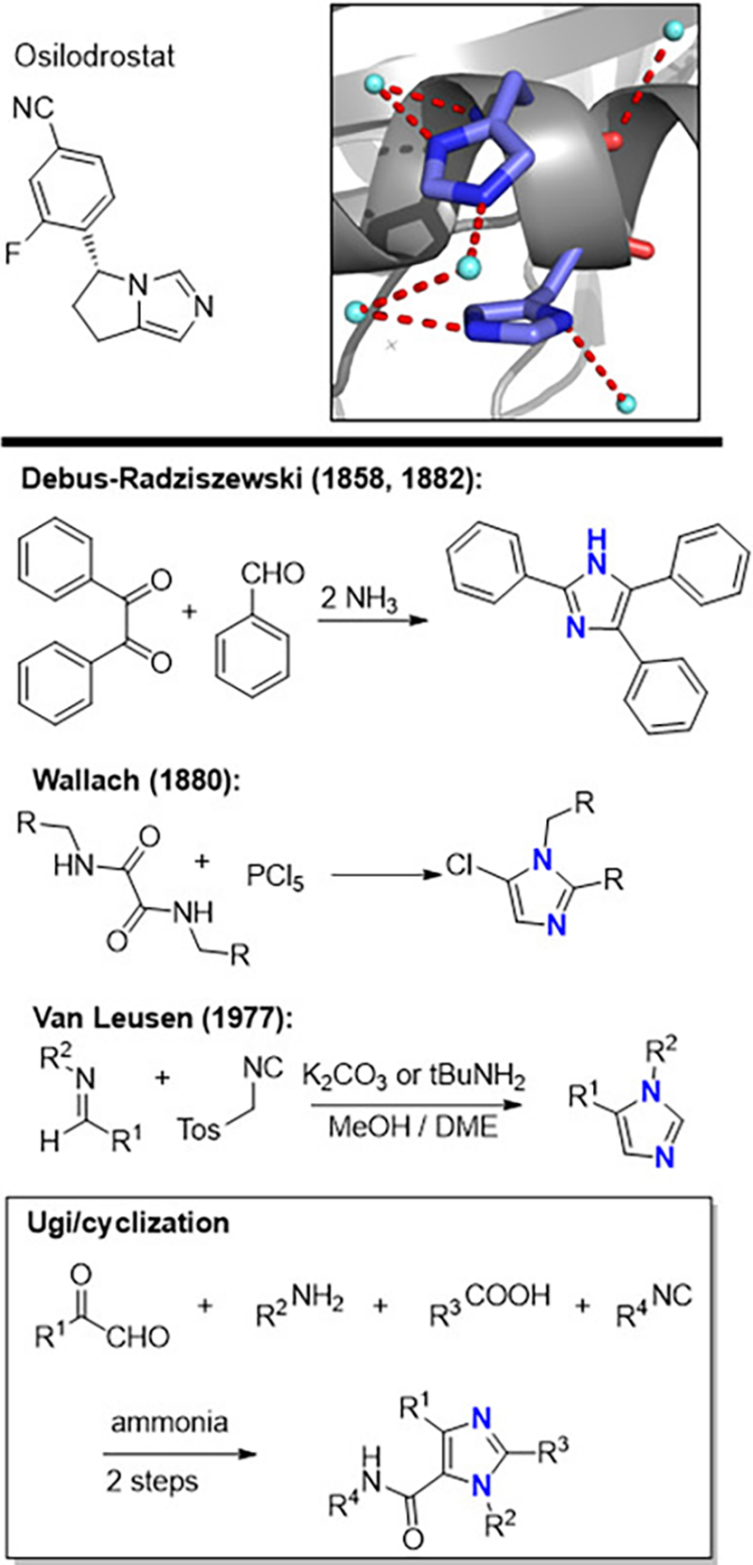
Importance of imidazole and their synthesis. Above (left) Osilodrostat is an inhibitor of 11β-hydroxylase for the treatment of Cushing’s disease. Above (right) His94 and His95 in the oncogene RAS forming a stacking interaction and involved in multiple hydrogen bondings with a water network (PDBB ID 6OIM). (Middle) Several classical imidazole synthese. (Below) 2-Step imidazole synthesis involving an Ugi reaction with four points of diversity.

In particular, general methods that allow independent variation at all four positions of the imidazole ring remain scarce. Isocyanide-based multicomponent reactions (IMCRs), such as the Ugi and Passerini reactions, are highly convergent transformations that have had a profound impact on diversity-oriented synthesis and drug discovery.^12^ Their modular nature, operational simplicity, and tolerance toward a wide range of functional groups make them attractive platforms for the rapid assembly of complex molecular architectures. In this context, Ugi-derived heterocycle syntheses have emerged as powerful postcondensation strategies for accessing heterocyclic scaffolds from common intermediates.^13^ Formation of imidazoles from Ugi adducts derived from glyoxal-type oxo components has been reported in a small number of studies.^14–17^ However, these examples were largely restricted to aryl glyoxals and provided limited insight into substrate scope, functional group tolerance, or reaction limitations. As a result, the broader synthetic potential of the Ugi-based imidazole transformation—particularly its applicability to aliphatic glyoxal derivatives and densely substituted imidazole frameworks—has remained underexplored. Prompted by these limitations, we sought to systematically investigate and optimize the Ugi-based synthesis of tetrasubstituted imidazoles. Special emphasis was placed on expanding the oxo-component scope to include previously unexplored aliphatic glyoxals, evaluating the influence of all four Ugi components on cyclization efficiency, and identifying practical reaction conditions that enable reliable one-pot access to highly substituted imidazole scaffolds. Herein, we report a comprehensive scope and optimization study that establishes the Ugi-imidazole transformation as a general and robust platform for the synthesis of tetrasubstituted imidazoles with full positional diversity.

## Results and Discussion

### Reaction Design and General Strategy

The synthetic strategy is based on a two-step, one-pot sequence comprising an Ugi four-component reaction (U-4CR) followed by an ammonium-induced cyclization to furnish tetrasubstituted imidazoles (Scheme 1). Glyoxal derivatives serve as the oxo component, enabling direct incorporation of substitution at the imidazole C4 position, while the amine, carboxylic acid, and isocyanide components independently define the remaining substitution pattern. This modular design allows full control over all four positions of the imidazole ring and renders the approach particularly attractive for diversity-oriented synthesis.

**Scheme 1:**
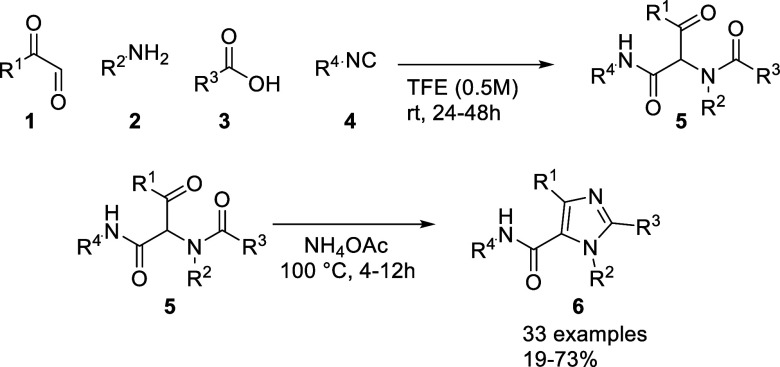
Preparation of Tetra-Substituted Imidazoles

### Preparation of α-Oxoaldehyde Starting Materials

Access to structurally diverse α-oxoaldehydes (glyoxal derivatives) was a prerequisite for evaluating the scope of the Ugi–imidazole transformation. Commercial access to α-oxoaldehydes is limited, and structurally diverse representatives are often available only at high cost, necessitating a reliable in-house preparation of these oxo components. Accordingly, the required glyoxal derivatives were generated from the corresponding methyl ketones via a microwave-assisted Riley oxidation using selenium dioxide (Scheme 2), providing rapid and efficient access to both aromatic and aliphatic α-oxoaldehydes,^18^ compared with other methods such as the Kornblum oxidation of aryl methyl ketones in DMSO,^19^ oxidation of diazoketones with Murray’s reagent,^20^ or reduction of oxoacetyl chlorides using tributyltin hydride.^21^ Under the optimized conditions, methyl ketones were oxidized in aqueous tetrahydrofuran under microwave irradiation, affording the desired α-oxoaldehydes within short reaction times. After completion, the reaction mixtures were filtered over Celite to remove selenium residues, concentrated, and directly redissolved in trifluoroethanol. Importantly, the crude α-oxoaldehyde solutions could be used directly in the subsequent Ugi reaction without chromatographic purification, demonstrating the operational simplicity and robustness of the overall protocol. No adverse effects on the Ugi reaction or the ammonium-induced cyclization step were observed when using these crude oxo components. This straightforward and scalable preparation of α-oxoaldehydes significantly expands the accessible substrate space for the Ugi-imidazole reaction and enables systematic exploration of both aryl and aliphatic substitution patterns at the imidazole C4 position.

**Scheme 2:**
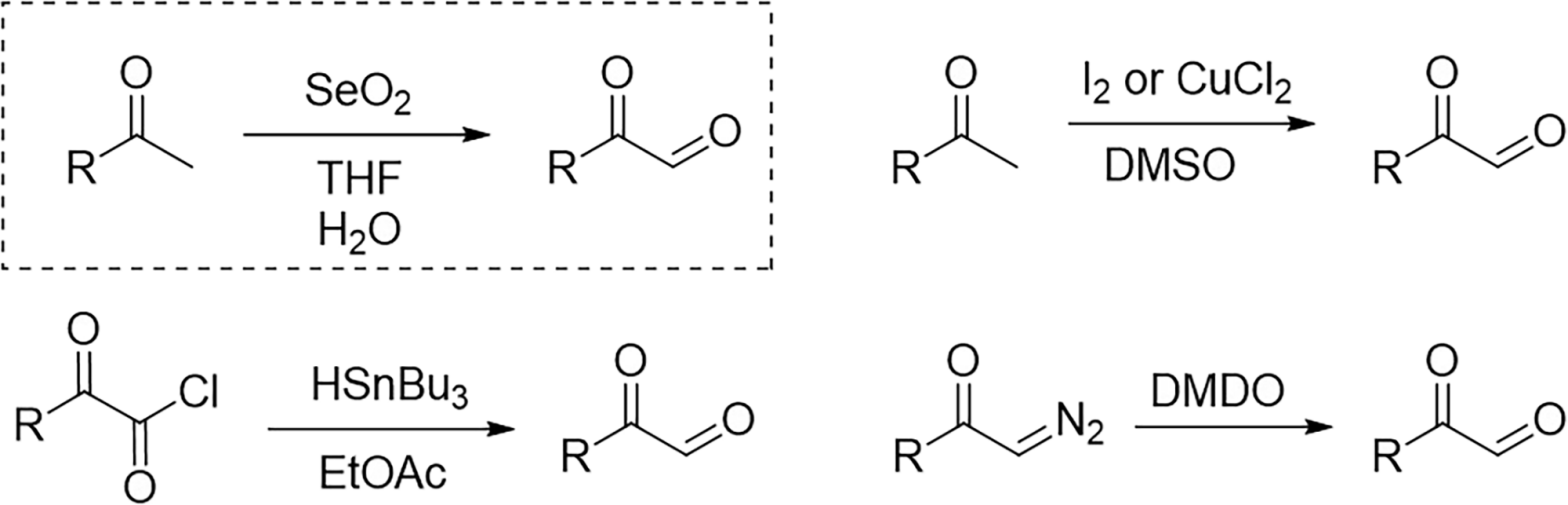
Synthetic Pathways towards Glyoxal Derivatives

### Reaction Optimization

Initial investigations revealed that the Ugi reaction involving glyoxal derivatives is highly sensitive to chemoselectivity and order of addition. Direct combination of all four Ugi components resulted in low conversion or complex mixtures. Optimal results were obtained when the glyoxal derivative was added dropwise as the final component to a preformed mixture of amine, carboxylic acid, and isocyanide in trifluoroethanol at room temperature. Under these conditions, the desired Ugi adducts were formed reproducibly. Following completion of the Ugi reaction, cyclization was induced by the addition of an ammonium source. A range of ammonium salts and ammonia equivalents were evaluated, including ammonium chloride, ammonium nitrate, ammonium sulfate, ammonium carbonate, ammonium hydroxide, and ammonium acetate. While several of these additives promoted imidazole formation, ammonium acetate consistently afforded the cleanest conversion with minimal byproduct formation. Increasing the amount of ammonium acetate accelerated the cyclization step, with 10 equiv identified as optimal. The optimized conditions therefore consist of reacting the amine, carboxylic acid, and isocyanide (1.0 equiv each) with the glyoxal derivative (1.5 equiv) in trifluoroethanol at room temperature for 12–24 h, followed by heating at 100 °C in the presence of ammonium acetate for 4–12 h. Under these conditions, tetrasubstituted imidazoles were obtained in low to excellent yields, depending on the nature of the Ugi components (Table 1).

**Table 1:**
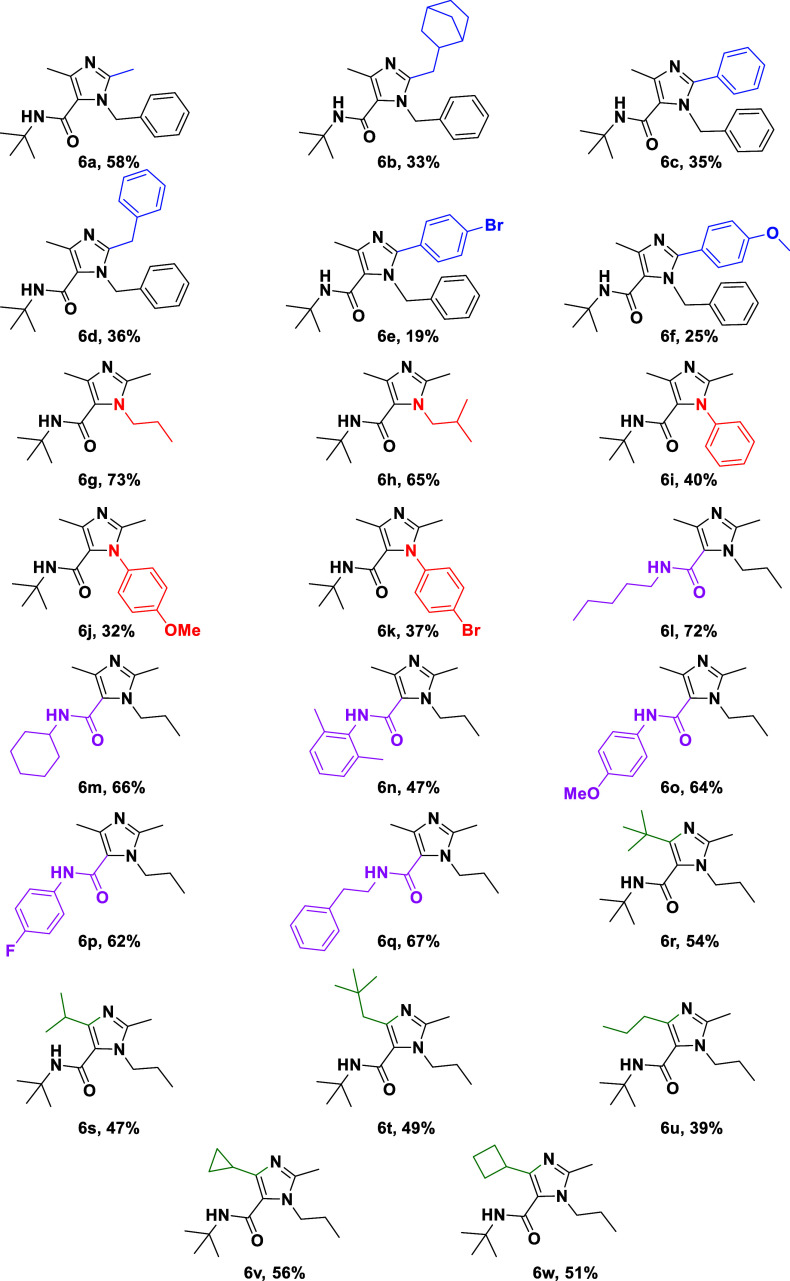
Synthesis of Tetra-Substituted Imidazoles **6**

### Scope with Respect to the Carboxylic Acid Component

The influence of the carboxylic acid component was first examined (Table 1, compounds **6a–f**). Aliphatic carboxylic acids generally afforded higher yields than aromatic acids, suggesting that steric and electronic factors play a significant role during the cyclization step. para-Substituted benzoic acids, irrespective of electron-donating or electron-withdrawing substituents, resulted in diminished yields. Ortho-substituted aromatic acids were particularly problematic. When 2-chlorobenzoic acid was employed, the expected imidazole product was not obtained, and mass spectrometric analysis indicated substitution of chlorine during the cyclization step. In the case of sterically demanding biphenyl-2-carboxylic acid, cyclization required prolonged heating and the isolated product exhibited limited stability, likely due to steric strain around the imidazole core. These observations delineate clear steric limitations for the acid component in this transformation.

### Scope with Respect to the Amine Component

Next, the effect of the amine component was evaluated (Table 1, compounds **6g–k**). Aliphatic amines consistently afforded the desired imidazoles in moderate to good yields, whereas aniline-derived amines were less effective. In these cases, reduced yields were observed despite formation of the corresponding Ugi intermediates, indicating that the cyclization step is particularly sensitive to the electronic nature of the amine substituent. The use of tritylamine provided further insight into the reaction pathway. While the Ugi adduct was detected by mass spectrometry, cyclization resulted in rapid cleavage of the trityl group, yielding a trisubstituted imidazole. This outcome highlights the compatibility of the reaction with acid-labile protecting groups but also illustrates a limitation when bulky, cation-stabilizing substituents are present. At the same time, this behavior provides a convenient entry to trisubstituted imidazoles from appropriately protected amines, offering an additional level of structural flexibility within the Ugi–imidazole framework.

### Scope with Respect to the Isocyanide Component

A broad range of isocyanides was compatible with the optimized conditions (Table 1, compounds **6l–q**). Both aliphatic and bulky isocyanides participated smoothly in the Ugi reaction and subsequent cyclization, generally affording the corresponding imidazoles in good yields. These results indicate that the isocyanide component is comparatively tolerant and does not represent a major limiting factor in this transformation.

### Scope with Respect to the Glyoxal Component

The scope of the glyoxal component was of particular interest, as prior studies had largely focused on aryl glyoxals.^14–17^ In the present work, both aryl and aliphatic glyoxal derivatives were successfully employed (Table 1, compounds **6r–w**). Glyoxals bearing two α-methylene positions resulted in slightly reduced yields, consistent with increased conformational flexibility and competing side reactions. In contrast, strongly electron-deficient glyoxals, such as trifluoropyruvic aldehyde, failed to undergo cyclization. This behavior can be rationalized by decreased nucleophilicity of the intermediate hemiaminal nitrogen, which hampers ring closure under ammonium-induced conditions. These findings provide useful guidance for future applications of the method and define clear electronic boundaries for the oxo component.

### Extended Scope and Functional Group Tolerance

To further demonstrate the versatility of the method, a selection of structurally diverse substrates was examined (Table 2). Notably, indole-3-acetic acid, a plant hormone derivative, was well tolerated, highlighting the compatibility of the protocol with heteroaromatic and biologically relevant motifs (**6ae**). In addition, Boc-protected amino acid survived the reaction conditions intact, affording imidazole products amenable to downstream functionalization (**6aa**). Similarly, mono Boc-protected diamines worked nicely (**6ab**).

**Table 2:**
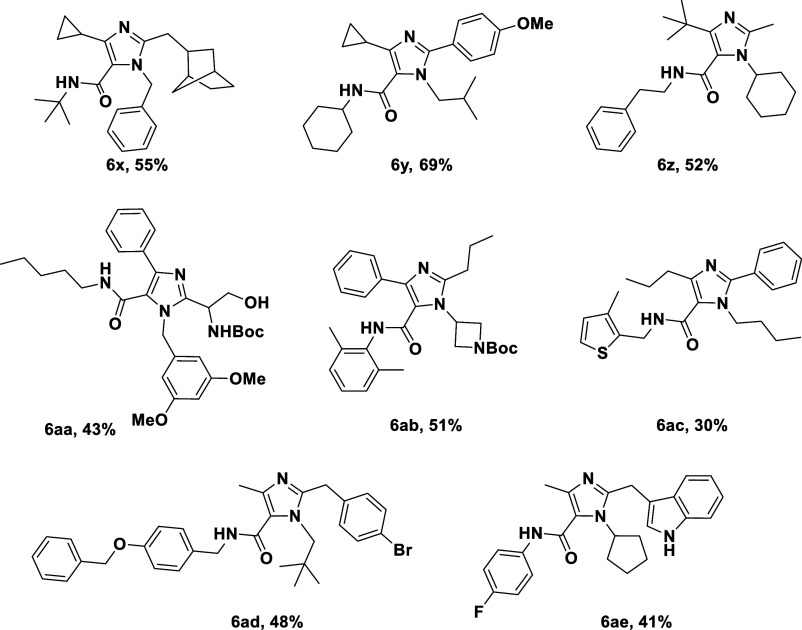
Extended Scope of Tetra-Substituted Imidazole **6**

Single-crystal X-ray diffraction analysis of compound **6o** unambiguously confirmed the imidazole core structure and substitution pattern, providing structural validation of the transformation (Figure 2).

**Figure 2:**
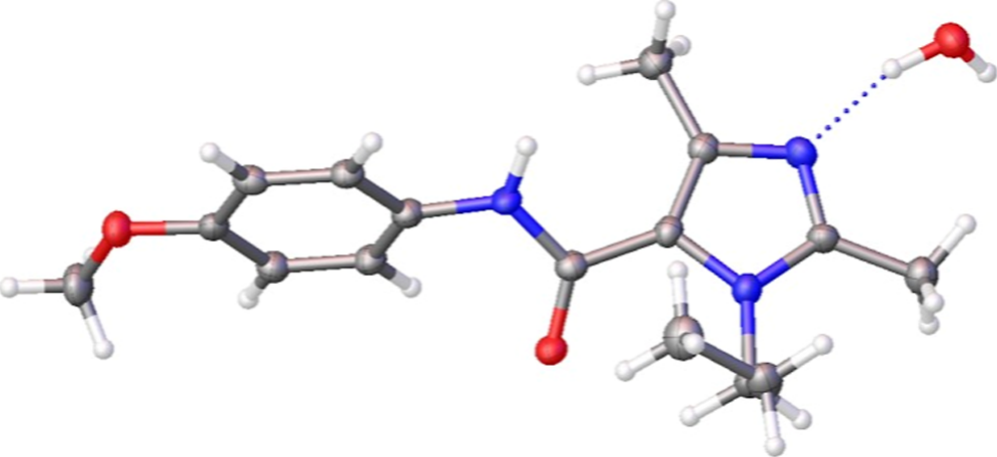
Crystal structure of tetra substituted imidazole **6o**.

### Mechanistic Considerations

A plausible mechanism for the ammonium-induced cyclization is depicted in Scheme 3. Initial formation of a hemiaminal through reaction of ammonia, generated via thermal decomposition of ammonium acetate,^22^ with the carbonyl group of the Ugi adduct is followed by intramolecular cyclization onto the second carbonyl functionality. Subsequent proton transfers and stepwise elimination of two molecules of water furnish the aromatic imidazole core. The observed sensitivity to electronic and steric effects is consistent with this mechanistic proposal.

**Scheme 3:**
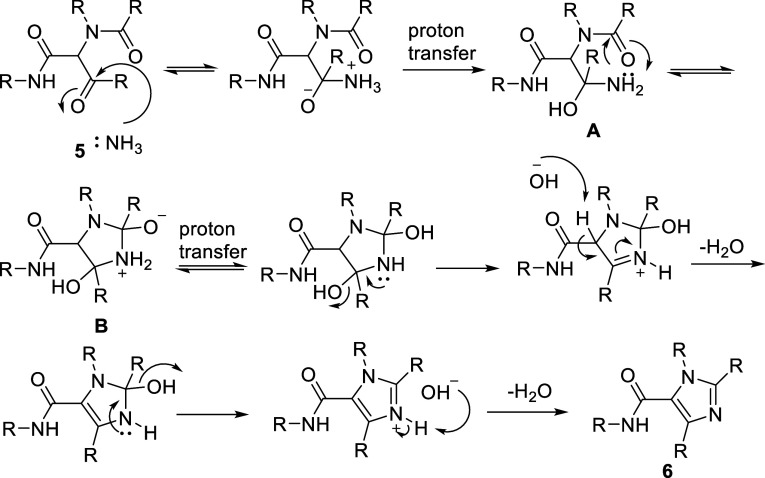
Proposed Reaction Mechanism of Ammonia Induced Cyclization of Ugi Adduct 5 towards Imidazole **6**

## Conclusions

In summary, a systematic expansion and optimization of an Ugi-based one-pot synthesis of tetrasubstituted imidazoles has been accomplished. By combining readily accessible α-oxoaldehydes with amines, carboxylic acids, and isocyanides, the method enables full substitution and regio selective control over all four positions of the imidazole ring. In contrast to earlier reports limited largely to aryl glyoxals, the present study demonstrates broad compatibility with both aliphatic and aromatic α-oxoaldehydes, significantly expanding the accessible chemical space. Key reaction parameters governing chemoselectivity and ammonium-induced cyclization were identified, resulting in a robust and operationally simple protocol that tolerates a wide range of functional groups. The ability to employ crude α-oxoaldehydes generated via microwave-assisted Riley oxidation further underscores the practicality of the approach. Scope studies delineated both the strengths and limitations of the transformation, providing useful guidance for future applications. Overall, this work establishes the Ugi-imidazole transformation as a reliable and diversity-oriented synthetic platform for the rapid generation of highly substituted imidazole scaffolds, with particular relevance for medicinal chemistry and small-molecule library synthesis.

## Methods

### General Procedure for the Synthesis of α-Oxoaldehydes

In a 5 mL microwave vial equipped with a magnetic stirring bar methylketone (1.0 mmol) was dissolved in THF (0.66 M, 1.5 mL) and H_2_O (60 μL). Selenium dioxide (1.1 mmol, 110.9 mg) was added and the vial was sealed with a cap. The reaction mixture was microwave irradiated at 160 °C, low absorption, for 30 min. Upon completion the crude product was filtered over Celite and flushed with DCM. The filtrate was concentrated in vacuo and redissolved in trifluoroethanol (1 mL). The crude solution was further used without purification.

### General Procedure for the Synthesis of Imidazoles

In a 4 mL glass vial equipped with a magnetic stirring bar amine (1.0 mmol, 1 equiv), carboxylic acid (1.0 mmol, 1 equiv), and isocyanide (1.0 mmol, 1 equiv) were dissolved in trifluoroethanol (1 M, 1 mL). The crude α-oxoaldehyde (1.5 mmol, 1.5 equiv) was dissolved in trifluoroethanol (1.5 M, 1 mL) and was dropwise added to the reaction. The reaction mixture was stirred for 12–24 h at room temperature. Ammonium acetate (770 mg, 10.0 mmol) was added and the reaction mixture was heated at 100 °C for 12–24 h. Upon completion, the reaction mixture was coated on silica and purified by column chromatography.

## Supplementary Material



## Notes

This is Crystallographic Information File of compound **6o** (CIF) Experimental procedures, characterization, NMR spectral data for compounds **6a–6ae**, and X-ray crystallographic structure determination for compound **6o** (PDF)
