# Supplementary material for: Systematic Expansion of an Ugi-Based Multicomponent Synthesis of Tetrasubstituted Imidazoles
Source: ACS Omega. 2026 Mar 27;11(13):20889–94. doi: 10.1021/acsomega.5c13353 (PMC13063188; doi:10.1021/acsomega.5c13353)
Supplement: Supplementary file 1 [file ao5c13353_si_001.pdf]

Supporting information for

Systematic Expansion of an Ugi-Based Multicomponent Synthesis of Tetrasubstituted Imidazoles

*Robin van der Straat,[1] Justyna Kalinowska-Tluscik,[2] Katarzyna Kurpiewska[2] and Alexander Dömling[3]**

[1] Department of Medicinal Chemistry, Photopharmacology and Imaging, Groningen Research Institute of Pharmacy, University of Groningen, 9713 AV Groningen, The Netherlands

[2] Department of Crystal Chemistry and Crystal Physics, Faculty of Chemistry, Jagiellonian University, Gronostajowa 2, 30-387 Krakow, Poland

[3] Innovative Chemistry Group, CATRIN, and Institute of Molecular and Translational Medicine, Faculty of Medicine and Dentistry, Palacky University, Olomouc, Czech Republic.

*Corresponding Author. E-mail: alexander.domling@upol.cz

Table of Contents

I. General information……………………………………………………………..S3

II. Experimental procedures and analytical data …………………………..S3-S13

III. Crystallographic data for compound **6o** …………………………........S14-S16

IV. Copies of ^{1}**H** and ^{13}**C NMR** spectra of all new compounds …………....S17-S47

V. References……………………………………………………………….S48

1. General Information

Nuclear magnetic resonance spectra were recorded on a Bruker Avance 500 spectrometer ^{1}H NMR (500 MHz), ^{13}C NMR (126 MHz). Chemical shifts for ^{1}H NMR were reported as δ values and coupling constants were in hertz (Hz); The following abbreviations were used for spin multiplicity: s = singlet, bs = broad singlet, d = doublet, t = triplet, q = quartet, quin = quintet, dd = double of doublets, ddd = double of doublet of doublets, m = multiplet. Chemical shifts for ^{13}C NMR reported in ppm relative to the solvent peak. Analytical thin-layer chromatography was performed using precoated silica gel 60 F_{254} plates (Merck, Darmstadt), and the spots were visualized with UV light at 254 nm or alternatively by staining with potassium permanganate, or ninhydrin solutions. Column chromatography was carried out with silica gel 60 (0.040–0.063 mm, 230–400 mesh). Reagents were available from commercial suppliers (Sigma Aldrich, ABCR, Acros, AK Scientific and Fluorochem) and used without any purification unless otherwise noted. Melting points were determined using a OEM Electrothermal melting point apparatus 1A 8103. Electrospray ionization mass spectra (ESI-MS) were recorded on a Waters Investigator Semi-prep 15 SFC-MS instrument. High resolution mass spectra were recorded using a QTOF Bruker Maxis Plus, mass range 100-1500 m/z, spectra rate 2.00 Hz.

2. Experimental procedures and analytical data

General procedure for the synthesis of α-oxoaldehydes:

In a 5-mL microwave vial equipped with a magnetic stirring bar methylketone (1.0 mmol) was dissolved in THF (0.66 M, 1.5 mL) and H_2O (60 μL). Selenium dioxide (1.1 mmol, 110.9 mg) was added and the vial was sealed with a cap. The reaction mixture was microwave irradiated at 160 ^{0}C, low absorption, for 30 minutes. Upon completion the crude product was filtered over celite and flushed with DCM. The filtrate was concentrated *in vacuo* and redissolved in trifluoroethanol (1 mL). The crude solution was further used without purification.

General procedure Imidazoles:

In a 4-mL glass vial equipped with a magnetic stirring bar amine (1.0 mmol, 1 eq.), carboxylic acid (1.0 mmol, 1 eq.), and isocyanide (1.0 mmol, 1 eq.) were dissolved in trifluoroethanol (1M, 1 mL). The crude α-oxoaldehyde (1.5 mmol, 1.5 eq.) was dissolved in trifluoroethanol (1.5 M, 1 mL) and was dropwise added to the reaction. The reaction mixture was stirred for 12-24 hours

at room temperature. Ammonium acetate (770 mg, 10.0 mmol) was added and the reaction mixture was heated at 100 °C for 12-24 hours. Upon completion, the reaction mixture was coated on silica and purified by column chromatography.

1-benzyl-*N*-(tert-butyl)-2,4-dimethyl-1*H*-imidazole-5-carboxamide (6a) was synthesized

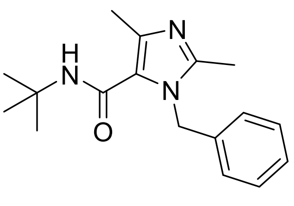

by following the general procedure to afford **6a** (166 mg, 58 %) as a brown oil; **^{1}H NMR** (500 MHz, $CDCl_3$) *δ* 7.34 – 7.19 (m, 3H), 7.09 – 7.02 (m, 2H), 5.44 (s, 1H), 5.40 (s, 2H), 2.37 (s, 3H), 2.33 (s, 3H), 1.36 (s, 9H) ppm. **^{13}C NMR** (126 MHz, $CDCl_3$) *δ* 161.1, 146.6, 137.3, 137.0, 128.7, 127.5, 126.8, 123.7, 51.7, 47.9, 28.9, 14.5, 13.2 ppm. **HRMS** (ESI), m/z calcd for $C_{17}H_{24}N_3O$ $[M+H]^+$ 286.1914, found 286.1913.

1-benzyl-2-(bicyclo[2.2.1]heptan-2-ylmethyl)-*N*-(tert-butyl)-4-methyl-1*H*-imidazole-5-carboxamide (6b) was synthesized by following the general procedure to afford **6b** (125 mg,

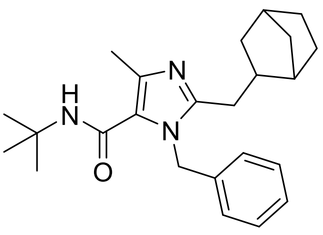

33 %) as an off-white solid; **mp** 93-95 °C**; ^{1}H NMR** (500 MHz, $CDCl_3$) *δ* 7.31 – 7.20 (m, 3H), 7.04 – 7.00 (m, 2H), 5.47 – 5.35 (m, 3H), 2.55 (dd, *J* = 14.8, 8.5 Hz, 1H), 2.43 (dd, *J* = 14.8, 7.5 Hz, 1H), 2.38 (s, 3H), 2.18 (s, 1H), 1.97 (s, 1H), 1.84 (m, 1H), 1.48 – 1.36 (m, 4H), 1.32 (s, 9H), 1.15 – 1.01 (m, 4H) ppm. **^{13}C NMR** (126 MHz, $CDCl_3$) δ 161.3, 149.7, 137.7, 137.6, 128.6, 127.4, 126.7, 123.5, 51.6, 47.7, 40.9, 40.6, 37.8, 36.8, 35.2, 33.7, 29.7, 28.9, 28.6, 14.9 ppm. **HRMS** (ESI), m/z calcd for $C_{24}H_{34}N_3O$ $[M+H]^+$ 380.2696, found 380.2696.

1-benzyl-*N*-(tert-butyl)-4-methyl-2-phenyl-1*H*-imidazole-5-carboxamide (6c) was

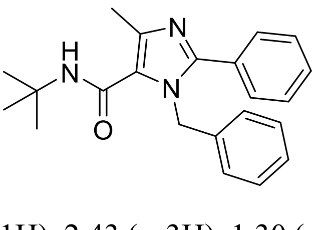

synthesized by following the general procedure to afford **6c** (120 mg, 35 %) as an off-white solid; **mp** 129-131 °C; **^{1}H NMR** (500 MHz, $CDCl_3$) *δ* 7.56 – 7.50 (m, 2H), 7.42 (dt, *J* = 4.9, 1.6 Hz, 3H), 7.28 – 7.18 (m, 3H), 6.96 (dd, *J* = 8.0, 1.6 Hz, 2H), 5.50 (s, 2H), 5.39 (s, 1H), 2.43 (s, 3H), 1.30 (s, 9H) ppm. **^{13}C NMR** (126 MHz, $CDCl_3$) *δ* 161.1, 149.6, 138.9, 137.8, 130.1, 129.4, 129.3, 128.7, 128.5, 127.4, 126.8, 124.7, 51.6, 48.8, 28.8, 14.8 ppm. **HRMS** (ESI), m/z calcd for $C_{22}H_{26}N_3O$ $[M+H]^+$ 348.2070, found 348.2069.

1,2-dibenzyl-*N*-(tert-butyl)-4-methyl-1*H*-imidazole-5-carboxamide (6d) was synthesized

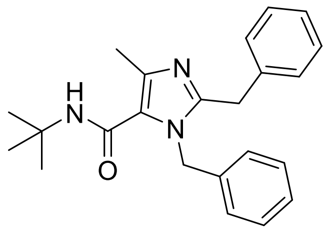

by following the general procedure to afford **6d** (129 mg, 36 %) as an off-white solid; **mp** 137-139 °C; **^{1}H NMR** (500 MHz, $CDCl_3$) δ 7.34 – 7.17 (m, 6H), 7.16 – 7.10 (m, 2H), 6.97 – 6.90 (m, 2H), 5.43 (s, 1H), 5.27 (s, 2H), 4.01 (s, 2H), 2.42 (s, 3H), 1.33 (s, 9H) ppm. **^{13}C NMR** (126 MHz, $CDCl_3$) δ 161.1, 148.3, 137.8, 137.1, 136.6, 128.7, 128.6, 128.3, 127.4, 126.8, 126.7, 124.2, 51.7, 47.8, 33.7, 28.9, 14.9 ppm. **HRMS** (ESI), m/z calcd for $C_{23}H_{28}N_3O$ $[M+H]^+$ 362.2227, found 362.2225.

1-benzyl-2-(4-bromophenyl)-*N*-(tert-butyl)-4-methyl-1*H*-imidazole-5-carboxamide (6e)

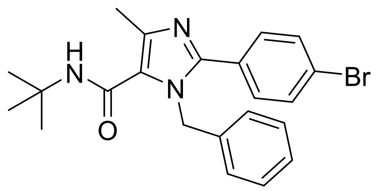

was synthesized by following the general procedure to afford **6e** (83 mg, 19 %) as a pale orange solid; **mp** 152-154 °C; **^{1}H NMR** (500 MHz, $CDCl_3$) δ 7.58 – 7.53 (m, 2H), 7.40 (d, J = 8.5 Hz, 2H), 7.25 (d, J = 11.0 Hz, 3H), 6.95 (dt, J = 7.7, 1.0 Hz, 2H), 5.47 (s, 2H), 5.40 (s, 1H), 2.43 (s, 3H), 1.30 (s, 9H) ppm. **^{13}C NMR** (126 MHz, $CDCl_3$) δ 160.9, 148.4, 138.9, 137.5, 131.9, 130.8, 128.9, 128.7, 127.6, 126.6, 125.1, 123.9, 51.8, 48.9, 28.8, 14.7 ppm. **HRMS** (ESI), m/z calcd for $C_{22}H_{25}BrN_3O$ $[M+H]^+$ 426.1176, found 426.1173.

1-benzyl-*N*-(tert-butyl)-2-(4-methoxyphenyl)-4-methyl-1*H*-imidazole-5-carboxamide (6f)

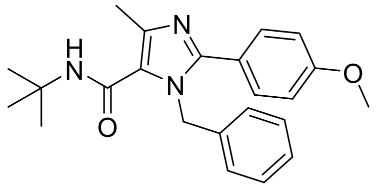

was synthesized by following the general procedure to afford **6f** (94 mg, 25 %) as a yellow solid; **mp** 134-136 °C; **^{1}H NMR** (500 MHz, $CDCl_3$) δ 7.46 (d, J = 8.6 Hz, 2H), 7.30 – 7.17 (m, 3H), 7.00 – 6.89 (m, 4H), 5.47 (s, 2H), 5.38 (s, 1H), 3.83 (s, 3H), 2.42 (s, 3H), 1.30 (s, 9H) ppm. **^{13}C NMR** (126 MHz, $CDCl_3$) δ 161.2, 160.4, 149.6, 138.8, 137.9, 130.7, 128.6, 127.4, 126.6, 124.5, 122.5, 114.1, 55.3, 51.6, 48.8, 28.8, 14.8 ppm. **HRMS** (ESI), m/z calcd for $C_{23}H_{28}N_3O_2$ $[M+H]^+$ 378.2176, found 378.2175.

***N*-(tert-butyl)-2,4-dimethyl-1-propyl-1*H*-imidazole-5-carboxamide (6g)** was synthesized

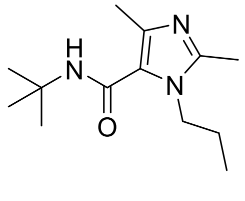

by following the general procedure to afford **6g** (173 mg, 73 %) as a brown solid; **mp** 106-108 °C; **^{1}H NMR** (500 MHz, $CDCl_3$) δ 5.49 (s, 1H), 4.10 (t, J = 7.3, 1.2 Hz, 2H), 2.34 (d, J = 5.8, 1.2 Hz, 6H), 1.74 – 1.62 (m, 3H), 1.45 (s, 9H), 0.89 (t, J = 7.4, 1.2 Hz, 3H) ppm. **^{13}C NMR** (126 MHz,

$CDCl_3$) δ 161.3, 146.1, 137.4, 123.3, 51.6, 46.3, 29.1, 24.1, 15.0, 13.2, 11.1 ppm. **HRMS** (ESI), m/z calcd for $C_{13}H_{24}N_3O$ $[M+H]^+$ 238.1914, found 238.1912.

***N*-(tert-butyl)-1-isobutyl-2,4-dimethyl-1*H*-imidazole-5-carboxamide (6h)** was synthesized

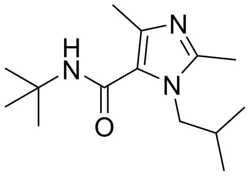

by following the general procedure to afford **6h** (164 mg, 65 %) as a brown solid; **mp** 125-127 °C; **^{1}H NMR** (500 MHz, $CDCl_3$) δ 5.49 (s, 1H), 4.00 (d, *J* = 7.4 Hz, 2H), 2.34 (d, *J* = 8.1, 1.0 Hz, 6H), 1.98 – 1.85 (m, 1H), 1.44 (s, 9H), 0.86 (d, 6H) ppm. **^{13}C NMR** (126 MHz, $CDCl_3$) δ 161.5, 146.4, 137.4, 123.5, 51.6, 51.5, 30.0, 29.0, 19.9, 14.9, 13.5 ppm. **HRMS** (ESI), m/z calcd for $C_{14}H_{26}N_3O$ $[M+H]^+$ 252.2070, found 252.2068.

***N*-(tert-butyl)-2,4-dimethyl-1-phenyl-1*H*-imidazole-5-carboxamide (6i)** was synthesized by

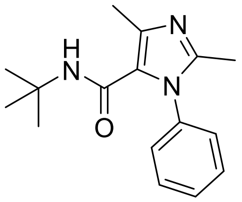

following the general procedure to afford **6i** (110 mg, 40 %) as an off white solid; **mp** 154-156 °C; ^{1}H NMR (500 MHz, $CDCl_3$) δ 7.61 – 7.43 (m, 3H), 7.32 – 7.20 (m, 2H), 4.93 (s, 1H), 2.48 (s, 3H), 2.19 (s, 3H), 1.16 (s, 9H). **^{13}C NMR** (126 MHz, $CDCl_3$) δ 159.8, 146.2, 141.6, 137.1, 129.8, 129.3, 127.3, 124.7, 51.3, 28.7, 14.6, 13.7 ppm. ^{13}C NMR (126 MHz, $CDCl_3$) δ 159.8, 146.2, 141.6, 137.2, 129.8, 129.2, 127.3, 124.7, 51.3, 28.7, 14.6, 13.7 ppm. **HRMS** (ESI), m/z calcd for $C_{16}H_{22}N_3O$ $[M+H]^+$ 272.1757, found 272.1756.

***N*-(tert-butyl)-1-(4-methoxyphenyl)-2,4-dimethyl-1*H*-imidazole-5-carboxamide (6j)** was

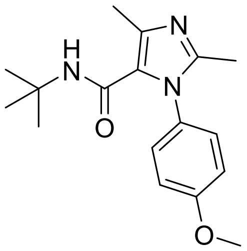

synthesized by following the general procedure to afford **6j** (146 mg, 32 %) as a brown solid; **mp** 88-90 °C; **^{1}H NMR** (500 MHz, $CDCl_3$) δ 7.22 – 7.15 (m, 2H), 7.06 – 6.98 (m, 2H), 5.01 (s, 1H), 3.87 (s, 3H), 2.48 (s, 3H), 2.17 (s, 3H), 1.18 (s, 9H) ppm. **^{13}C NMR** (126 MHz, $CDCl_3$) δ 160.1, 159.8, 146.6, 141.4, 129.5, 128.4, 124.7, 114.9, 55.6, 51.3, 28.7, 14.5, 13.5 ppm. **HRMS** (ESI), m/z calcd for $C_{17}H_{24}N_3O_2$ $[M+H]^+$ 302.1863, found 302.1858.

1-(4-bromophenyl)-*N*-(tert-butyl)-2,4-dimethyl-1*H*-imidazole-5-carboxamide (6k) was synthesized by following the general procedure to afford **6k** (129 mg, 37 %) as a pale orange solid; **mp** 150-152 °C; **^{1}H NMR** (500 MHz, $CDCl_3$) δ 7.68 – 7.54 (m, 2H), 7.17 – 7.02 (m, 2H), 5.20 (s, 1H), 2.44 (s, 3H), 2.18 (s, 3H), 1.27 (s, 9H) ppm. **^{13}C NMR** (126 MHz, $CDCl_3$) δ 159.8, 146.3, 139.9, 136.3, 132.7, 128.6, 125.1, 122.9, 51.6, 28.8, 14.6, 13.7 ppm. **HRMS** (ESI), m/z calcd for $C_{16}H_{21}BrN_3O$ $[M+H]^+$ 350.0863, found 350.0863.

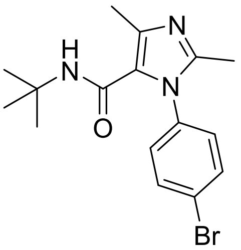

2,4-dimethyl-*N*-pentyl-1-propyl-1*H*-imidazole-5-carboxamide (6l) was synthesized by following the general procedure to afford **6l** (182 mg, 72 %) as a sticky red oil; **^{1}H NMR** (500 MHz, $CDCl_3$) δ 5.72 (t, *J* = 5.8 Hz, 1H), 4.12 (t, 2H), 3.41 (q, *J* = 7.2, 5.8 Hz, 2H), 2.38 (s, 3H), 2.35 (s, 3H), 1.75 – 1.64 (m, 2H), 1.66 – 1.56 (m, 2H), 1.42 – 1.31 (m, 4H), 0.91 (m, 6H) ppm. **^{13}C NMR** (126 MHz, $CDCl_3$) δ 161.7, 146.4, 138.0, 122.3, 46.5, 39.4, 29.5, 29.2, 24.1, 22.4, 15.1, 14.0, 13.2, 11.1 ppm. **HRMS** (ESI), m/z calcd for $C_{14}H_{26}N_3O$ $[M+H]^+$ 252.2070, found 252.2069.

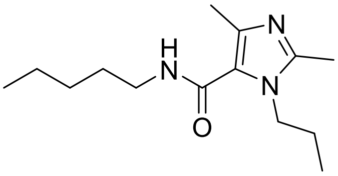

***N*-cyclohexyl-2,4-dimethyl-1-propyl-1*H*-imidazole-5-carboxamide (6m)** was synthesized by following the general procedure to afford **6m** (173 mg, 66 %) as a pale brown solid; **mp** 57-59 °C; **^{1}H NMR** (500 MHz, $CDCl_3$) δ 5.53 (d, *J* = 8.0 Hz, 1H), 4.15 – 4.07 (m, 2H), 3.99 – 3.88 (m, 1H), 2.37 (s, 3H), 2.35 (s, 3H), 2.05 – 1.98 (m, 2H), 1.81 – 1.57 (m, 4H), 1.50 – 1.35 (m, 2H), 1.31 – 1.17 (m, 4H), 0.91 (t, *J* = 7.5 Hz, 3H) ppm. **^{13}C NMR** (126 MHz, CDCl3) δ 160.8, 146.3, 137.9, 48.1, 46.6, 33.3, 29.7, 25.5, 24.8, 24.2, 15.2, 13.2, 11.1 ppm. **HRMS** (ESI), m/z calcd for $C_{15}H_{26}N_3O$ $[M+H]^+$ 264.2070, found 264.2069.

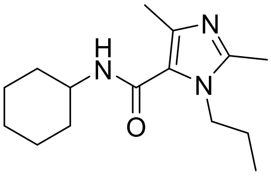

***N*-(2,6-dimethylphenyl)-2,4-dimethyl-1-propyl-1*H*-imidazole-5-carboxamide (6n)** was synthesized by following the general procedure to afford **6n** (135 mg, 47 %) as a brown solid; **mp** 77-79 °C**;** **^{1}H NMR** (500 MHz, $CDCl_3$) δ 7.16 – 7.10 (m, 3H), 6.90 (s, 1H), 4.17 (t, 2H), 2.56 (s, 3H), 2.40 (s, 3H), 2.29 (s, 6H), 1.78 – 1.67 (m, 2H), 0.92 (t, *J* = 7.4 Hz, 3H) ppm. **^{13}C NMR** (126 MHz, $CDCl_3$) δ 160.0, 147.2, 139.2, 135.4, 133.5, 128.4, 127.5, 46.7, 24.3, 18.8,

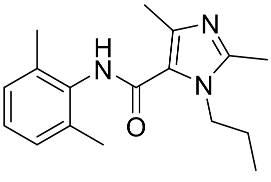

15.8, 13.3, 11.1 ppm. **HRMS** (ESI), m/z calcd for $C_{17}H_{24}N_3O$ [M+H]$^+$ 286.1914, found 286.1913.

***N*-(4-methoxyphenyl)-2,4-dimethyl-1-propyl-1*H*-imidazole-5-carboxamide (6o)** was

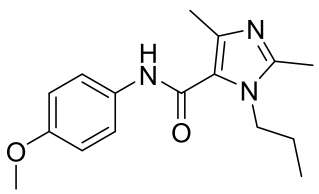

synthesized by following the general procedure to afford **6o** (185 mg, 64 %) as a brown solid; **mp** 79-81 °C**;** **^{1}H NMR** (500 MHz, $CDCl_3$) δ 7.50 – 7.42 (m, 2H), 7.30 (s, 1H), 6.94 – 6.87 (m, 2H), 4.18 – 4.12 (m, 2H), 3.81 (s, 3H), 2.49 (s, 3H), 2.39 (s, 3H), 1.80 – 1.68 (m, 2H), 0.92 (t, *J* = 7.4 Hz, 3H) ppm. **^{13}C NMR** (126 MHz, $CDCl_3$) δ 159.6, 156.7, 147.2, 138.9, 130.6, 122.3, 122.1, 114.3, 55.6, 46.7, 24.2, 15.5, 13.3, 11.1 ppm. **HRMS** (ESI), m/z calcd for $C_{16}H_{22}N_3O_2$ [M+H]$^+$ 288.1707, found 288.1703.

***N*-(4-fluorophenyl)-2,4-dimethyl-1-propyl-1*H*-imidazole-5-carboxamide (6p)** was

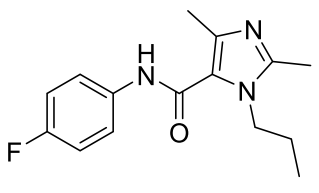

synthesized by following the general procedure to afford **6p** (171 mg, 62 %) as a brown solid; **mp** 45-47 °C**;** **^{1}H NMR** (500 MHz, $CDCl_3$) δ 7.54 – 7.48 (m, 2H), 7.46 – 7.41 (m, 1H), 7.10 – 7.01 (m, 2H), 4.15 (t, 2H), 2.49 (s, 3H), 2.39 (s, 3H), 1.80 – 1.67 (m, 2H), 0.92 (t, *J* = 7.4 Hz, 3H) ppm. **^{13}C NMR** (126 MHz, $CDCl_3$) δ 160.5, 159.6, 158.6, 147.4, 139.3, 133.6, 133.6, 122.0, 121.2, 115.9, 115.7, 46.7, 24.2, 15.5, 13.3, 11.1 ppm. **HRMS** (ESI), m/z calcd for $C_{15}H_{19}FN_3O$ [M+H]$^+$ 276.1507, found 276.1506.

2,4-dimethyl-*N*-phenethyl-1-propyl-1*H*-imidazole-5-carboxamide (6q) was synthesized by

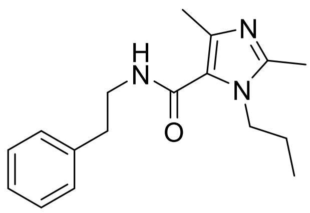

following the general procedure to afford **6q** (193 mg, 67 %) as a brown solid; **mp** 55-56 °C**;** **^{1}H NMR** (500 MHz, $CDCl_3$) δ 7.37 – 7.29 (m, 2H), 7.28 – 7.20 (m, 3H), 5.61 (s, 1H), 4.11 (t, 2H), 3.71 (q, *J* = 6.8, 5.8 Hz, 2H), 2.93 (t, *J* = 6.8 Hz, 2H), 2.34 (s, 3H), 2.14 (s, 3H), 1.74 – 1.62 (m, 2H), 0.90 (t, *J* = 7.4 Hz, 3H) ppm. **^{13}C NMR** (126 MHz, $CDCl_3$) δ 161.6, 146.5, 138.6, 138.4, 128.8, 126.7, 122.0, 46.6, 40.4, 35.6, 24.1, 15.1, 13.2, 11.1 ppm. **HRMS** (ESI), m/z calcd for $C_{17}H_{24}N_3O$ [M+H]$^+$ 286.1914, found 286.1911.

N,4-di-tert-butyl-2-methyl-1-propyl-1H-imidazole-5-carboxamide (6r) was synthesized by following the general procedure to afford **6r** (151 mg, 54 %) as a white solid; **mp** 157-159 °C**;** **^{1}H NMR** (500 MHz, $CDCl_3$) δ 5.64 (s, 1H), 3.77 (t, 2H), 2.33 (s, 3H), 1.77 – 1.64 (m, 2H), 1.45 (s, 9H), 1.34 (s, 9H), 0.92 (t, J = 7.4 Hz, 3H) ppm. **^{13}C NMR** (126 MHz, $CDCl_3$) δ 163.6, 146.1, 142.8, 123.6, 52.0, 46.2, 32.7, 30.7, 28.5, 24.2, 13.2, 11.3 ppm. **HRMS** (ESI), m/z calcd for $C_{16}H_{30}N_3O$ $[M+H]^+$ 280.2383, found 280.2381.

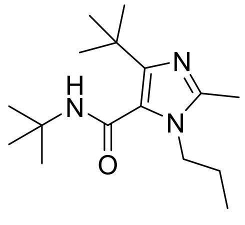

N-(tert-butyl)-4-isopropyl-2-methyl-1-propyl-1H-imidazole-5-carboxamide (6s) was synthesized by following the general procedure to afford **6s** (125 mg, 47 %) as a yellow solid; **mp** 53-55 °C; ^{1}H NMR (500 MHz, $CDCl_3$) δ 5.56 (s, 1H), 4.03 (t, 2H), 3.13 – 3.05 (m, 1H), 2.37 (s, 3H), 1.73 – 1.62 (m, 2H), 1.45 (s, 9H), 1.29 (d, J = 6.8 Hz, 6H), 0.90 (t, J = 7.4 Hz, 3H) ppm. **^{13}C NMR** (126 MHz, $CDCl_3$) δ 161.4, 146.4, 145.9, 122.3, 51.7, 46.0, 28.9, 27.0, 24.0, 22.7, 12.9, 11.2 ppm. **HRMS** (ESI), m/z calcd for $C_{15}H_{28}N_3O$ $[M+H]^+$ 266.2227, found 266.2226.

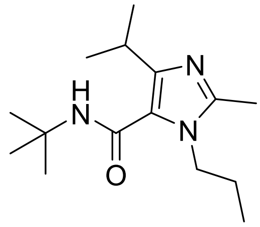

N-(tert-butyl)-2-methyl-4-neopentyl-1-propyl-1H-imidazole-5-carboxamide (6t) was synthesized by following the general procedure to afford **6t** (143 mg, 49 %) as a pale yellow solid; **mp** 109-111 °C; **^{1}H NMR** (500 MHz, $CDCl_3$) δ 5.64 (s, 1H), 4.06 (t, 2H), 2.55 (s, 2H), 2.34 (s, 3H), 1.70 – 1.60 (m, 2H), 1.44 (s, 9H), 0.96 (s, 9H), 0.88 (t, J = 7.5 Hz, 3H) ppm. **^{13}C NMR** (126 MHz, $CDCl_3$) δ 161.8, 145.3, 139.0, 125.2, 51.6, 46.0, 41.9, 32.4, 29.8, 28.9, 24.1, 13.3, 11.1 ppm. **HRMS** (ESI), m/z calcd for $C_{17}H_{32}N_3O$ $[M+H]^+$ 294.2540, found 294.2537.

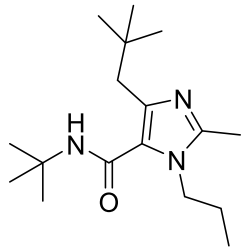

N-(tert-butyl)-2-methyl-1,4-dipropyl-1H-imidazole-5-carboxamide (6u) was synthesized by following the general procedure to afford **6u** (102 mg, 39 %) as a pale orange oil; **^{1}H NMR** (500 MHz, $CDCl_3$) δ 5.60 (s, 1H), 4.08 (t, 2H), 2.63 (t, 2H), 2.36 (s, 3H), 1.68 (hd, J = 7.4, 5.8 Hz, 4H), 1.45 (s, 9H), 0.97 (t, J = 7.4 Hz, 3H), 0.89 (t, J = 7.4 Hz, 3H) ppm. **^{13}C NMR** (126 MHz, $CDCl_3$)

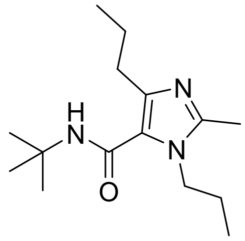

δ 161.2, 146.0, 141.3, 123.4, 51.6, 46.1, 30.6, 28.9, 24.0, 23.4, 14.1, 12.9, 11.1 ppm. **HRMS** (ESI), m/z calcd for $C_{15}H_{28}N_3O$ [M+H]$^+$ 266.2227, found 266.2226.

***N*-(tert-butyl)-4-cyclopropyl-2-methyl-1-propyl-1*H*-imidazole-5-carboxamide (6v)** was

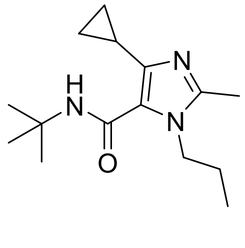

synthesized by following the general procedure to afford **6v** (147 mg, 56 %) as a yellow oil; **^{1}H NMR** (500 MHz, $CDCl_3$) δ 6.28 (s, 1H), 4.14 (t, 2H), 2.32 (s, 3H), 1.94 – 1.85 (m, 1H), 1.74 – 1.64 (m, 2H), 1.46 (s, 9H), 1.01 – 0.86 (m, 7H) ppm. **^{13}C NMR** (126 MHz, $CDCl_3$) δ 161.1, 145.8, 142.4, 123.6, 51.4, 46.4, 29.1, 24.1, 13.3, 11.2, 9.6, 7.0 ppm. **HRMS** (ESI), m/z calcd for $C_{15}H_{26}N_3O$ [M+H]$^+$ 264.2070, found 264.2069.

***N*-(tert-butyl)-4-cyclobutyl-2-methyl-1-propyl-1*H*-imidazole-5-carboxamide (6w)** was

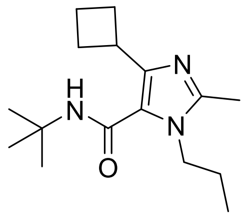

synthesized by following the general procedure to afford **6w** (141 mg, 51 %) as a yellow oil; **^{1}H NMR** (500 MHz, $CDCl_3$) δ 5.48 (s, 1H), 4.07 (t, 2H), 3.67 – 3.56 (m, 1H), 2.51 – 2.40 (m, 2H), 2.38 (s, 3H), 2.29 – 2.19 (m, 2H), 2.07 – 1.87 (m, 2H), 1.73 – 1.63 (m, 2H), 1.45 (s, 9H), 0.90 (t, *J* = 7.5 Hz, 3H) ppm. **^{13}C NMR** (126 MHz, $CDCl_3$) δ 161.2, 146.0, 144.4, 122.3, 51.6, 46.2, 33.6, 28.9, 28.3, 24.1, 18.5, 13.2, 11.2 ppm. **HRMS** (ESI), m/z calcd for $C_{16}H_{28}N_3O$ [M+H]$^+$ 278.2227, found 278.2226.

1-benzyl-2-(bicyclo[2.2.1]heptan-2-yl)-*N*-(tert-butyl)-4-cyclopropyl-1*H*-imidazole-5-

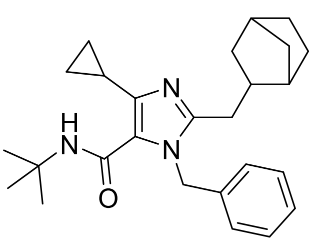

carboxamide (6x) was synthesized by following the general procedure to afford **6x** (222 mg, 55 %) as a pale yellow solid; **mp** 107-109 °C; **^{1}H NMR** (500 MHz, $CDCl_3$) δ 7.31 – 7.23 (m, 2H), 7.25 – 7.18 (m, 1H), 7.01 (d, 2H), 6.13 (s, 1H), 5.48 (q, *J* = 15.9 Hz, 2H), 2.45 (dd, 2H), 2.18 – 2.13 (m, 1H), 1.96 – 1.91 (m, 1H), 1.84 – 1.75 (m, 1H), 1.46 – 1.38 (m, 2H), 1.36 (s, 9H), 1.35 – 1.22 (m, 2H), 1.10 – 0.88 (m, 7H) ppm. **^{13}C NMR** (126 MHz, $CDCl_3$) δ 161.2, 149.7, 137.7, 128.6, 127.2, 126.6, 123.6, 51.4, 47.7, 41.0, 40.6, 37.7, 36.8, 35.2, 33.5, 29.7, 28.9, 28.6, 9.5, 7.3 ppm. **HRMS** (ESI), m/z calcd for $C_{26}H_{36}N_3O$ [M+H]$^+$ 406.2853, found 406.2848.

N-cyclohexyl-4-cyclopropyl-1-isobutyl-2-(4-methoxyphenyl)-1H-imidazole-5-

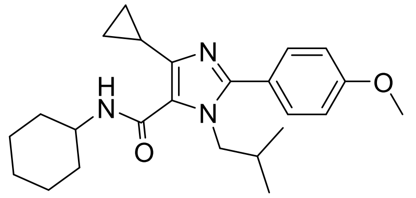

carboxamide (6y) was synthesized by following the general procedure to afford **6y** (272 mg, 69 %) as a white solid; **mp** 201-203 °C; **^{1}H NMR** (500 MHz, $CDCl_3$) δ 7.44 – 7.37 (m, 2H), 6.98 – 6.91 (m, 2H), 6.35 (d, *J* = 8.2 Hz, 1H), 4.15 (d, *J* = 7.4 Hz, 2H), 4.04 – 3.93 (m, 1H), 3.84 (s, 3H), 2.10 – 2.02 (m, 2H), 2.02 – 1.93 (m, 1H), 1.89 – 1.79 (m, 1H), 1.81 – 1.71 (m, 2H), 1.70 – 1.64 (m, 1H), 1.51 – 1.39 (m, 2H), 1.33 – 1.20 (m, 3H), 1.11 – 1.02 (m, 2H), 0.99 – 0.92 (m, 2H), 0.63 (d, *J* = 6.7 Hz, 6H) ppm. **^{13}C NMR** (126 MHz, $CDCl_3$) δ 161.0, 160.1, 149.9, 144.1, 131.0, 123.8, 123.4, 114.0, 55.3, 52.7, 48.1, 33.4, 30.1, 25.6, 24.8, 19.7, 9.7 7.3 ppm. **HRMS** (ESI), m/z calcd for $C_{24}H_{34}N_3O_2$ $[M+H]^+$ 396.2646, found 396.2641.

4-(tert-butyl)-1-cyclohexyl-2-methyl-N-phenethyl-1H-imidazole-5-carboxamide (6z) was

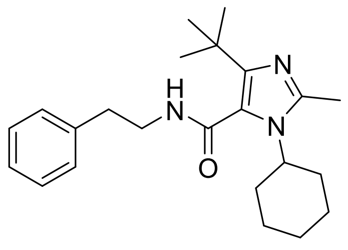

synthesized by following the general procedure to afford **6z** (192 mg, 52 %) as an off white solid; **mp** 238-240 °C; **^{1}H NMR** (500 MHz, $CDCl_3$) δ 7.35 – 7.28 (m, 2H), 7.27 – 7.19 (m, 3H), 5.76 (s, 1H), 3.84 – 3.73 (m, 1H), 3.71 (q, *J* = 7.0, 5.6 Hz, 2H), 2.93 (t, *J* = 6.9 Hz, 2H), 2.39 (s, 3H), 1.92 – 1.80 (m, 6H), 1.71 – 1.64 (m, 2H), 1.33 – 1.26 (m, 1H), 1.24 (s, 9H), 1.21 – 1.09 (m, 1H) ppm. **^{13}C NMR** (126 MHz, $CDCl_3$) δ 165.3, 146.0, 142.6, 138.6, 128.8, 128.6, 126.8, 122.4, 77.3, 77.0, 76.8, 57.5, 41.0, 35.0, 32.7, 32.1, 30.5, 26.3, 25.2, 15.2 ppm. **HRMS** (ESI), m/z calcd for $C_{23}H_{34}N_3O$ $[M+H]^+$ 368.2696, found 368.2693.

***tert*-butyl (1-(1-(3,5-dimethoxybenzyl)-5-(pentylcarbamoyl)-4-phenyl-1H-imidazol-2-yl)-**

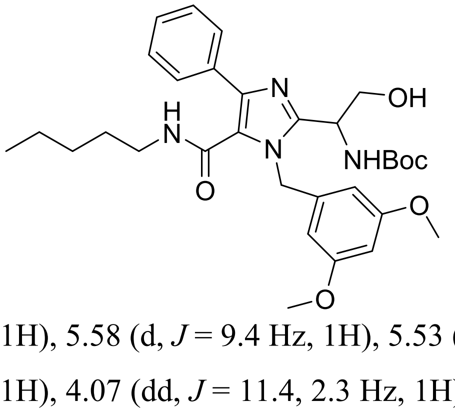

2-hydroxyethyl)carbamate (6aa) was synthesized by following the general procedure to afford **6aa** (243 mg, 43 %) as a yellow solid; **mp** 138-140 °C; **^{1}H NMR** (500 MHz, $CDCl_3$) δ 7.62 – 7.56 (m, 2H), 7.44 – 7.39 (m, 2H), 7.38 – 7.33 (m, 1H), 6.31 (t, *J* = 2.3 Hz, 1H), 6.22 (d, *J* = 2.3 Hz, 2H), 5.72 (d, *J* = 16.1 Hz, 1H), 5.63 (t, *J* = 5.9 Hz, 1H), 5.58 (d, *J* = 9.4 Hz, 1H), 5.53 (d, *J* = 16.1 Hz, 1H), 4.92 (dt, *J* = 9.5, 3.0 Hz, 1H), 4.76 (s, 1H), 4.07 (dd, *J* = 11.4, 2.3 Hz, 1H), 3.83 (dd, *J* = 11.6, 3.6 Hz, 1H), 3.74 (s, 6H), 3.21 – 3.12

(m, 2H), 1.38 (s, 9H), 1.31 – 1.14 (m, 4H), 1.08 – 0.98 (m, 2H), 0.81 (t, J = 7.3 Hz, 3H) ppm. **^{13}C NMR** (126 MHz, $CDCl_3$) δ 161.0, 155.1, 149.7, 140.6, 139.5, 133.0, 128.7, 128.5, 128.4, 123.0, 104.6, 99.1, 80.1, 65.0, 55.3, 47.9, 46.4, 39.6, 28.9, 28.6, 28.2, 22.3, 13.9 ppm. **HRMS** (ESI), m/z calcd for $C_{31}H_{43}N_4O_6$ $[M+H]^+$ 567.3177, found 567.3175.

***tert*-butyl-3-(5-((2,6-dimethylphenyl)carbamoyl)-4-phenyl-2-propyl-1*H*-imidazol-1-yl)azetidine-1-carboxylate (6ab)** was synthesized by following the general procedure to afford **6ab** (251 mg, 51 %) as a pale red solid; **mp** 151-153 °C; **^{1}H NMR** (500 MHz, $CDCl_3$) δ 7.62 – 7.55 (m, 2H), 7.48 – 7.35 (m, 3H), 7.09 – 7.03 (m, 1H), 7.04 – 6.97 (m, 3H), 5.77 – 5.67 (m, 1H), 4.45 – 4.35 (m, 4H), 2.83 (t, 2H), 2.06 (s, 6H), 1.95 – 1.84 (m, 2H), 1.42 (s, 9H), 1.06 (t, J = 7.3 Hz, 3H) ppm. **^{13}C NMR** (126 MHz, $CDCl_3$) δ 159.3, 156.3, 152.0, 143.8, 135.5, 134.4, 133.3, 132.0, 129.7, 129.1, 128.6, 128.2, 127.5, 127.4, 122.6, 80.2, 56.6, 44.8, 30.4, 28.3, 22.0, 18.7, 14.1 ppm. **HRMS** (ESI), m/z calcd for $C_{29}H_{37}N_4O_3$ $[M+H]^+$ 489.2858, found 489.2859.

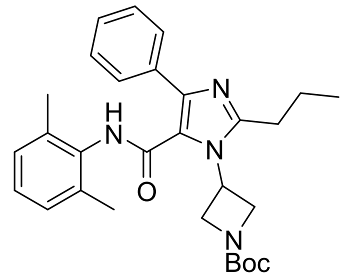

1-butyl-*N*-((3-methylthiophen-2-yl)methyl)-2-phenyl-4-propyl-1*H*-imidazole-5-carboxamide (6ac) was synthesized by following the general procedure to afford **6ac** (120 mg, 30 %) as an orange solid; **mp** 97-99 °C; **^{1}H NMR** (500 MHz, MeOD) δ 7.52 (s, 5H), 7.21 (d, J = 5.1 Hz, 1H), 6.83 (d, J = 5.1 Hz, 1H), 4.67 (s, 2H), 4.17 (t, J = 7.4 Hz, 2H), 2.67 (t, J = 7.7 Hz, 2H), 2.30 (s, 3H), 1.66 – 1.53 (m, 2H), 1.52 – 1.41 (m, 2H), 1.08 – 0.96 (m, 2H), 0.87 (t, J = 7.7 Hz, 3H), 0.71 (t, J = 7.4 Hz, 3H) ppm. **^{13}C NMR** (126 MHz, MeOD) δ 163.7, 150.6, 144.7, 136.0, 135.3, 131.3, 130.9, 130.8, 130.6, 129.9, 125.3, 124.3, 46.3, 36.9, 34.0, 30.6, 24.3, 20.5, 14.1, 13.7 ppm. **HRMS** (ESI), m/z calcd for $C_{23}H_{30}N_3OS$ $[M+H]^+$ 396.2104, found 396.2098.

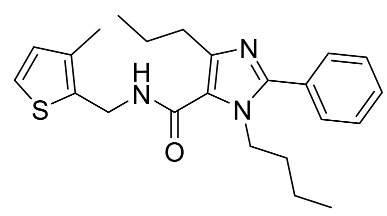

***N*-(4-(benzyloxy)phenyl)-2-(4-bromobenzyl)-4-methyl-1-neopentyl-1*H*-imidazole-5-carboxamide (6ad)** was synthesized by following the general procedure to afford **6ad** (134 mg, 48 %) as an off white solid; **mp** 143-145 °C; **^{1}H NMR** (500 MHz, MeOD) δ 7.47 –

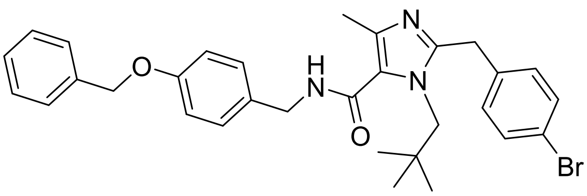

7.40 (m, 4H), 7.39 – 7.32 (m, 2H), 7.32 – 7.26 (m, 3H), 7.09 (d, *J* = 8.4 Hz, 2H), 6.99 – 6.93 (m, 2H), 5.08 (s, 2H), 4.41 (s, 2H), 4.09 (s, 2H), 2.26 (s, 3H), 0.84 (s, 9H) ppm. **^{13}C NMR** (126 MHz, $CDCl_3$) δ 162.2, 158.4, 149.0, 138.7, 136.6, 135.9, 131.8, 130.3, 130.1, 129.3, 128.6, 128.0, 127.4, 120.7, 115.2, 70.1, 54.2, 43.2, 34.2, 33.4, 27.9, 15.2 ppm. **HRMS** (ESI), m/z calcd for $C_{31}H_{35}BrN_3O_2$ $[M+H]^+$ 560.1907, found 560.1904.

2-((1*H*-indol-3-yl)methyl)-1-cyclopentyl-*N*-(4-fluorophenyl)-4-methyl-1*H*-imidazole-5-

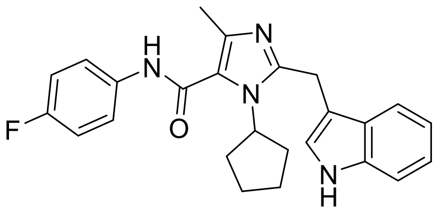

carboxamide (6ae) was synthesized by following the general procedure to afford **6ae** (172 mg, 41 %) as yellow solid; **mp** 207-210 °C; **^{1}H NMR** (500 MHz, MeOD) δ 7.64 – 7.57 (m, 2H), 7.49 (d, *J* = 8.0 Hz, 1H), 7.34 (d, *J* = 8.2 Hz, 1H), 7.09 (q, *J* = 9.0, 8.4 Hz, 3H), 7.02 – 6.95 (m, 2H), 4.81 – 4.71 (m, 1H), 4.27 (s, 2H), 2.34 (s, 3H), 2.06 – 1.98 (m, 3H), 1.77 – 1.64 (m, 3H), 1.49 – 1.39 (m, 1H). **^{13}C NMR** (126 MHz, $CDCl_3$) δ 160.6, 160.3, 158.6, 149.4, 139.1, 136.3, 133.7, 126.9, 123.6, 122.4, 122.3, 121.8, 121.8, 119.6, 118.7, 116.0, 115.8, 111.6, 111.2, 77.3, 77.0, 76.8, 58.1, 31.6, 25.1, 24.8, 14.9 ppm. **HRMS** (ESI), m/z calcd for $C_{25}H_{26}FN_4O$ $[M+H]^+$ 417.2085, found 417.2080.

3. Crystallographic data for compound 6o

X-ray diffraction data for single crystal of compound **6o** was collected using XtalLAB Synergy-S (Rigaku - Oxford Diffraction) four circle diffractometer with a mirror monochromator and a microfocus CuKα radiation source (λ = 1.5418 Å). Additionally, the diffractometer was equipped with the CryoStream cryostat system allowing low temperature experiments,

performed at 100(2) K. The obtained data was processed with CrysAlisPro software. [S1] The phase problem was solved with direct methods using SIR2014. [S2] Parameters of obtained models were refined by full-matrix least-squares on F^2 using SHELXL-2014/6 [S3]. Calculations were performed using WinGX integrated system (ver. 2014.1). [S4] Figure was prepared with Mercury 4.0 software [S5].

All non-hydrogen atoms were refined anisotropically. All hydrogen atoms attached to carbon atoms were positioned with the idealised geometry and refined using the riding model with the isotropic displacement parameter $U_{iso}[H] = 1.2\ U_{eq}[C]$ for all but methyl groups, where $U_{iso}[H] = 1.5\ U_{eq}[C]$ was applied. The difference Fourier map was inspected in order to find positions of hydrogen atoms linked to secondary amine N8, involved in the peptide bond. The structure is a monohydrate. The water molecule was positioned according to the positive peak at the Fourier difference map and refined with no restrains on its geometrical parameters. Crystal data and refinement results for the presented crystal structure are shown in Table S1. The asymmetric unit is shown in Figure S1A.

Compound **6o** crystallises as a monohydrate, in the non-centrosymmetric space group $P2_12_12_1$. The Flack parameter is 0.1(3), which shows that the absolute configuration is not reliably determined. The chirality of the crystal is based on the arrangement of the organic molecules in the proximity of a water zigzag chains propagating along [100] direction. These chains ($C_1^1(2)$ in the graph set notation [S6]) are formed by the hydrogen bonding system, with the water molecule in the dual role of acceptor and donor. Each water molecule is also a donor in O1w-H...N4 interaction formed with nitrogen atom of the 1,3-diazole ring as an acceptor (a discrete motif $D_1^1(2)$). Additionally, the crystal structure is stabilised by N8-H...O7 hydrogen bonds, forming a chain motif $C_1^1(4)$, parallel to [100] axis (Figure S1B). This interaction is observed between atoms involved in the peptide bond of the neighbouring molecules of compound **6o**.

Crystallographic data have been deposited with the Cambridge Crystallographic Data Centre as supplementary publication no. CCDC 2023038. Copies of the data can be obtained, free of charge, on application to CCDC, 12 Union Road, Cambridge CB2 1EZ, UK, (e-mail: deposit@ccdc.cam.ac.uk).

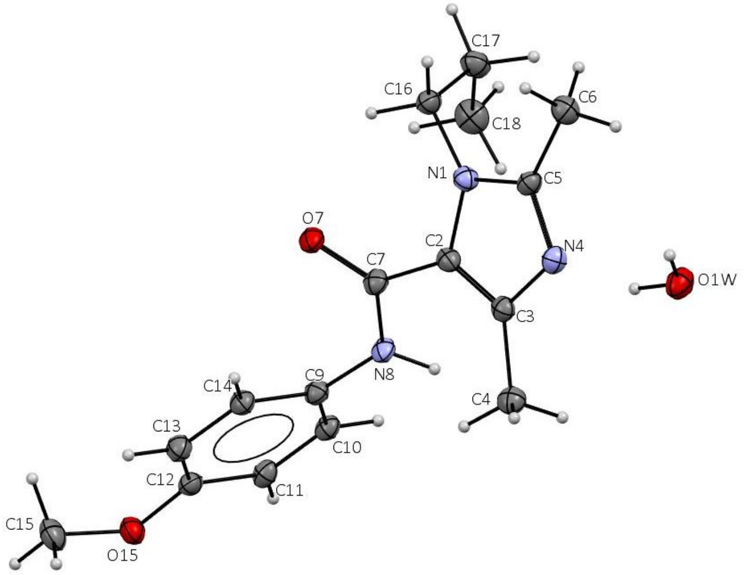

A

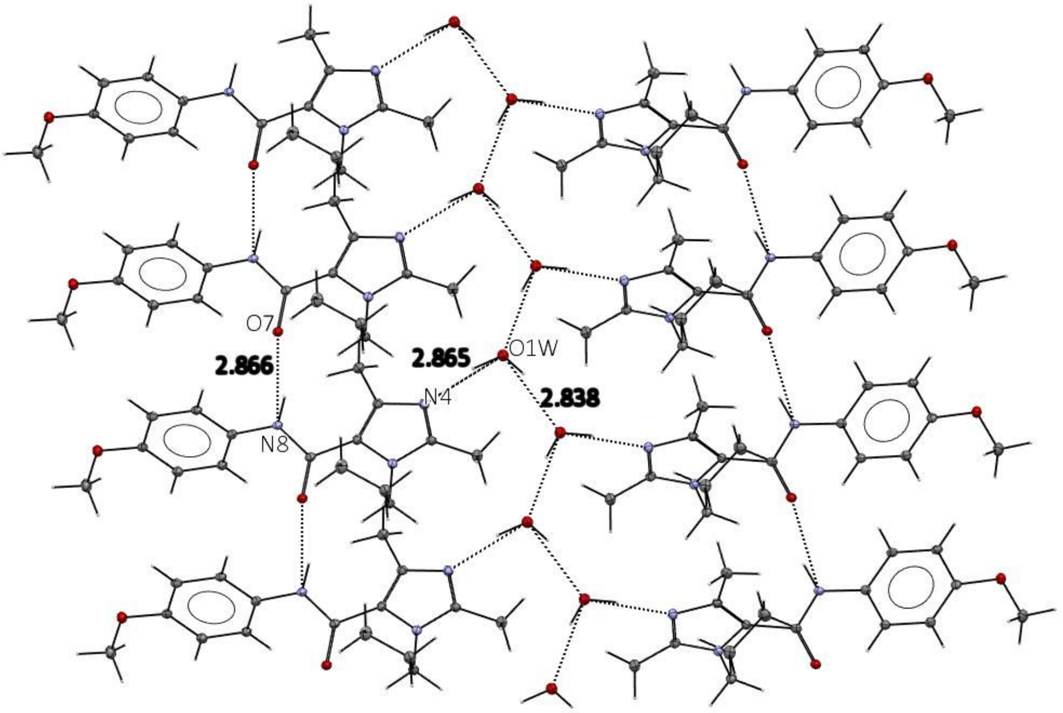

B

Figure S1. Molecular geometry observed in the crystal structure of compound **6o**: **A** – asymmetric unit with the atom labelling scheme; **B** – hydrogen bonding motifs stabilising the crystal structure. The chain motifs are propagating along [100] direction. Presented numerical values concern the donor-acceptor distances expressed in Å. Displacement ellipsoids of non-hydrogen atoms are drawn at the 30% probability level. H atoms are presented as small spheres with an arbitrary radius.

Table S1. Crystal data and structure refinement results for compounds **6o**

	**6o**
Empirical moiety formula	$C_{16} H_{21} N_3 O_2$, $H_2 O$
Formula weight [g/mol]	305.37
Crystal system	Orthorhombic
Space group	$P2_12_12_1$
Unite cell dimensions	a = 5.0027(2) Å<br>b = 8.0864(5) Å<br>c = 39.664 (3) Å<br>α= 90°<br>β= 90°<br>γ= 90°
Volume [Å^3]	1604.57(16)
Z	4
D_{calc} [Mg/m^3]	1.264
μ [mm^{-1}]	0.719
F(000)	656
Crystal size [mm^3]	0.3 x 0.1 x 0.01
Θ range	4.46° to 78.06°
Index ranges	-5 ≤ h ≤ 6,<br>-10 ≤ k ≤ 10,<br>-50 ≤ l ≤ 45
Refl. collected	16384
Independent reflections	3358<br>[R(int) = 0.1117]
Completeness [%] to<br>Θ = 67.68°	99.9
Absorption correction	Multi-scan
Tmin. and Tmax.	0. 761 and 1.000
Data/ restraints/parameters	3358 / 0 / 215
GooF on F2	1.126
Final R indices [I>2sigma(I)]	R1= 0.0720,<br>wR2= 0.1732
R indices (all data)	R1= 0.0781,<br>wR2= 0.1791
$\Delta\rho_{max}$, $\Delta\rho_{min}$ [e·Å^{-3}]	0.32 and -0.37

^{1}H and ^{13}C NMR spectra of compound 6a

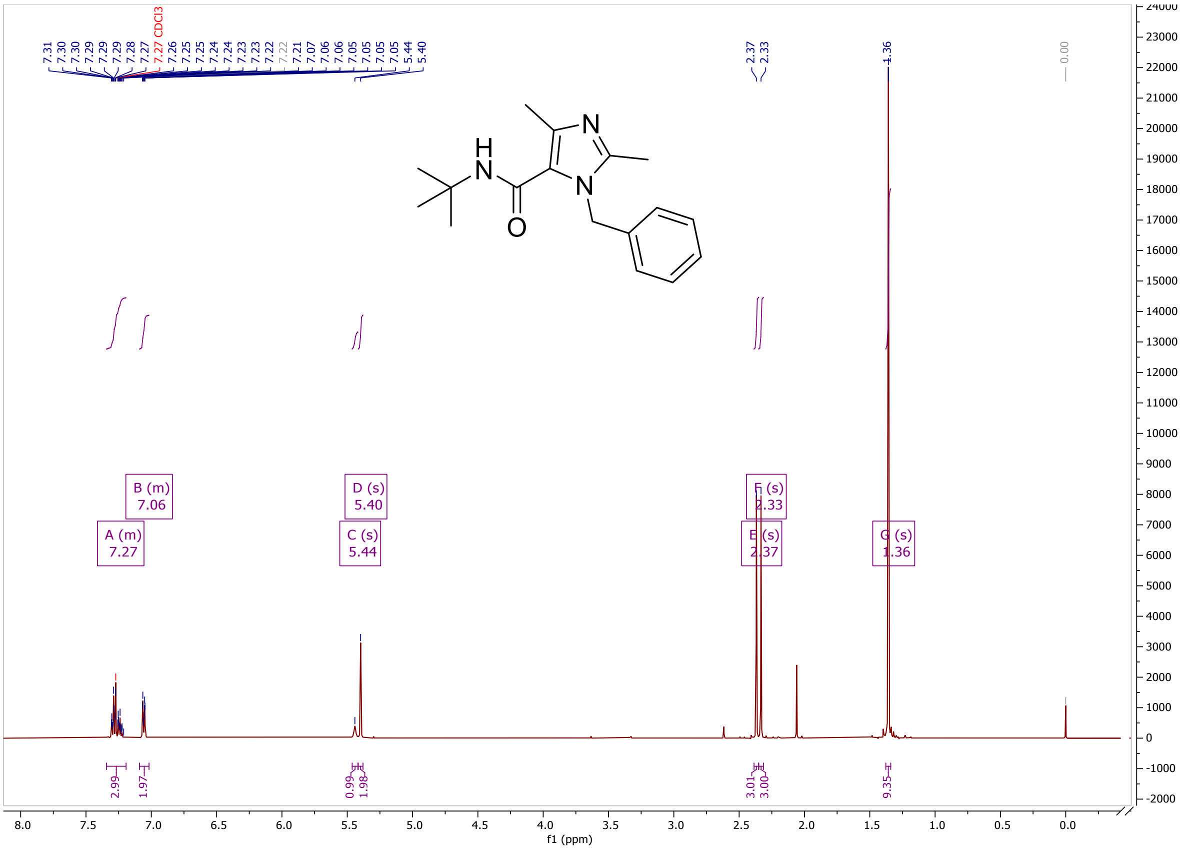

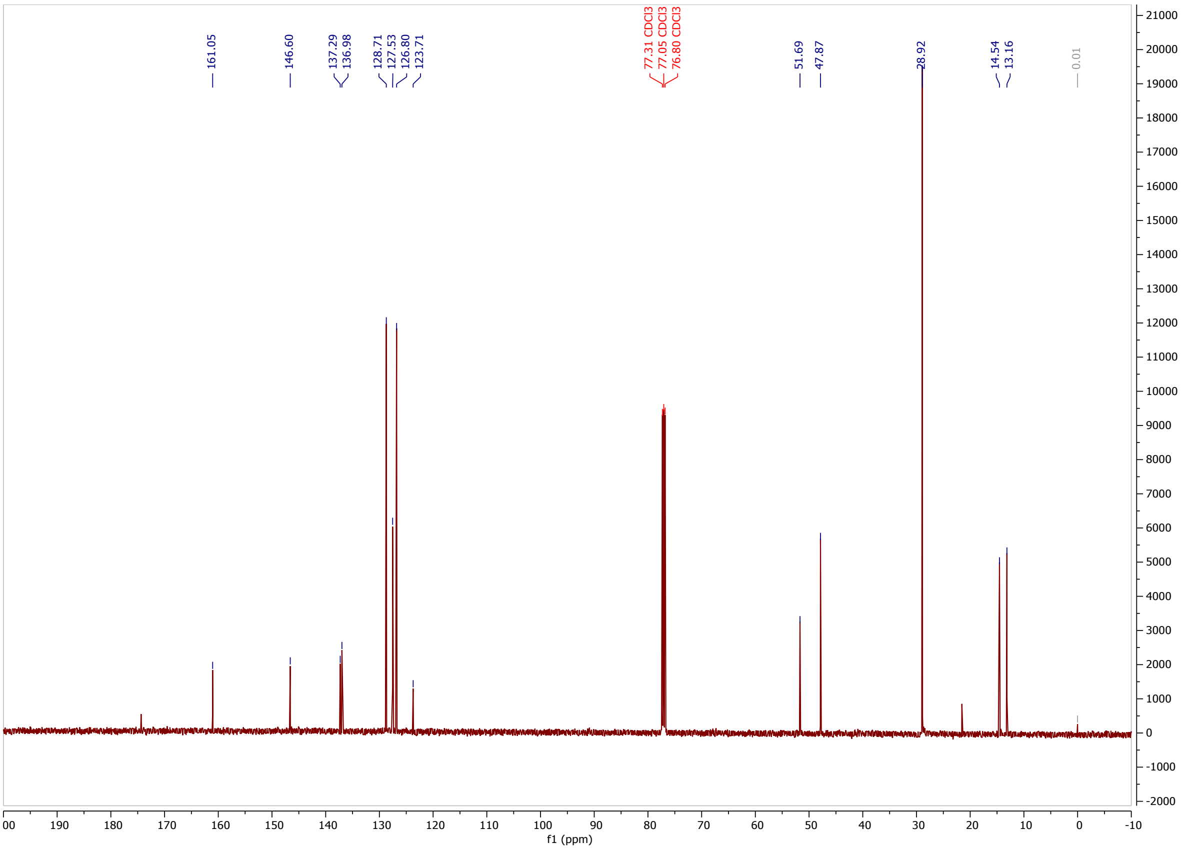

^{1}H and ^{13}C NMR spectra of compound 6b

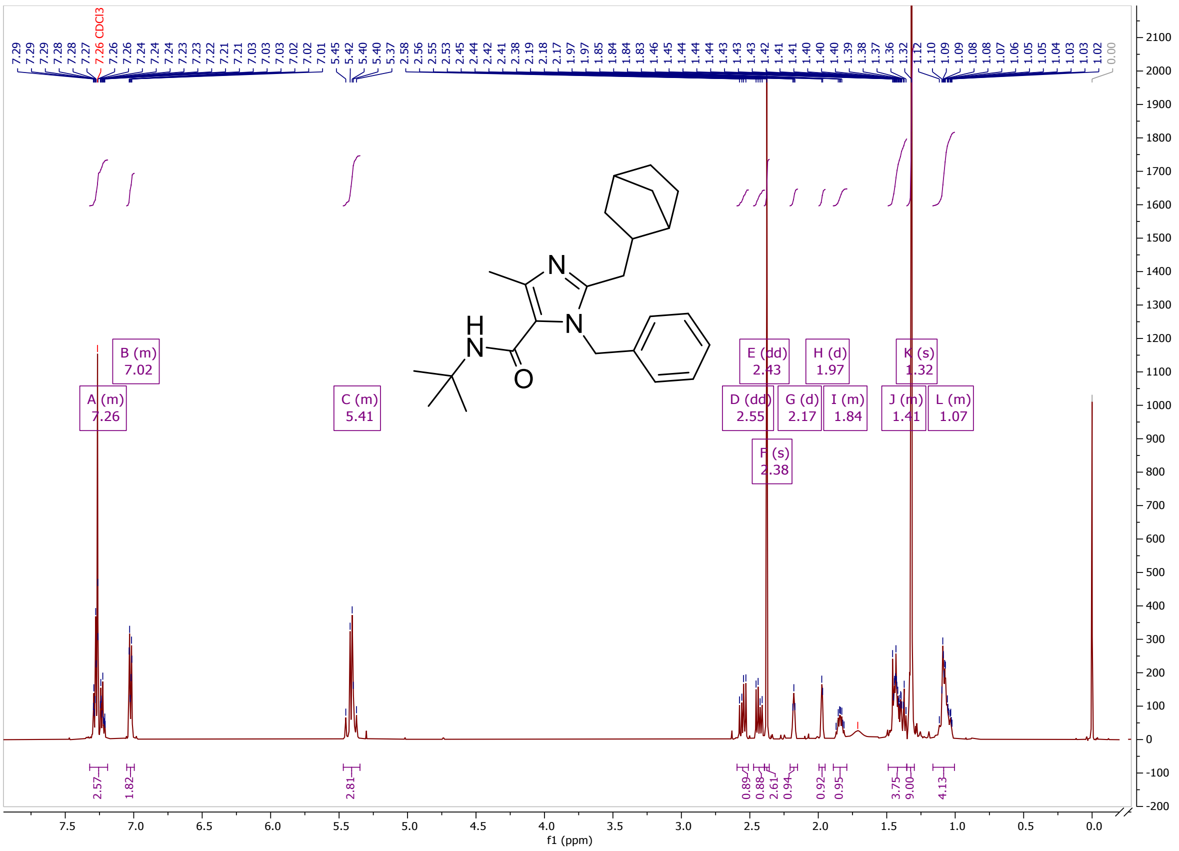

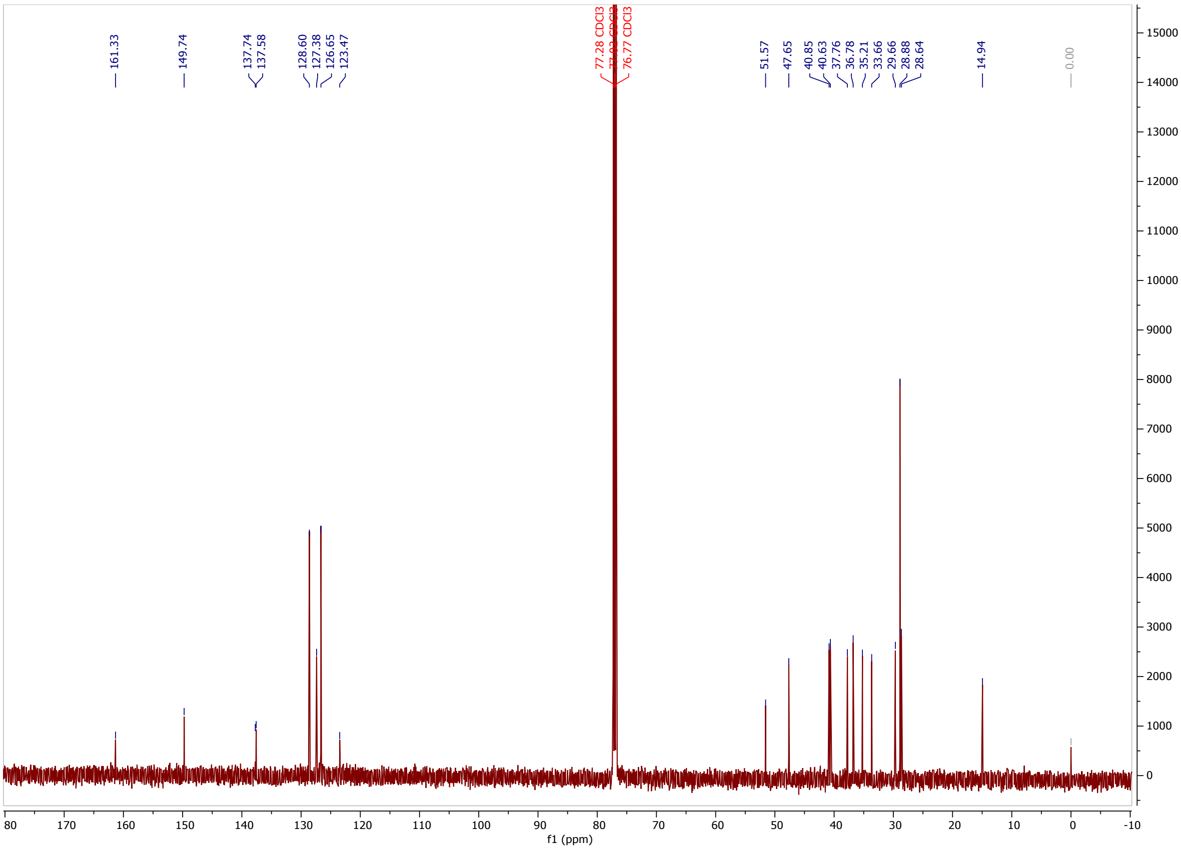

^{1}H and ^{13}C NMR spectra of compound 6c

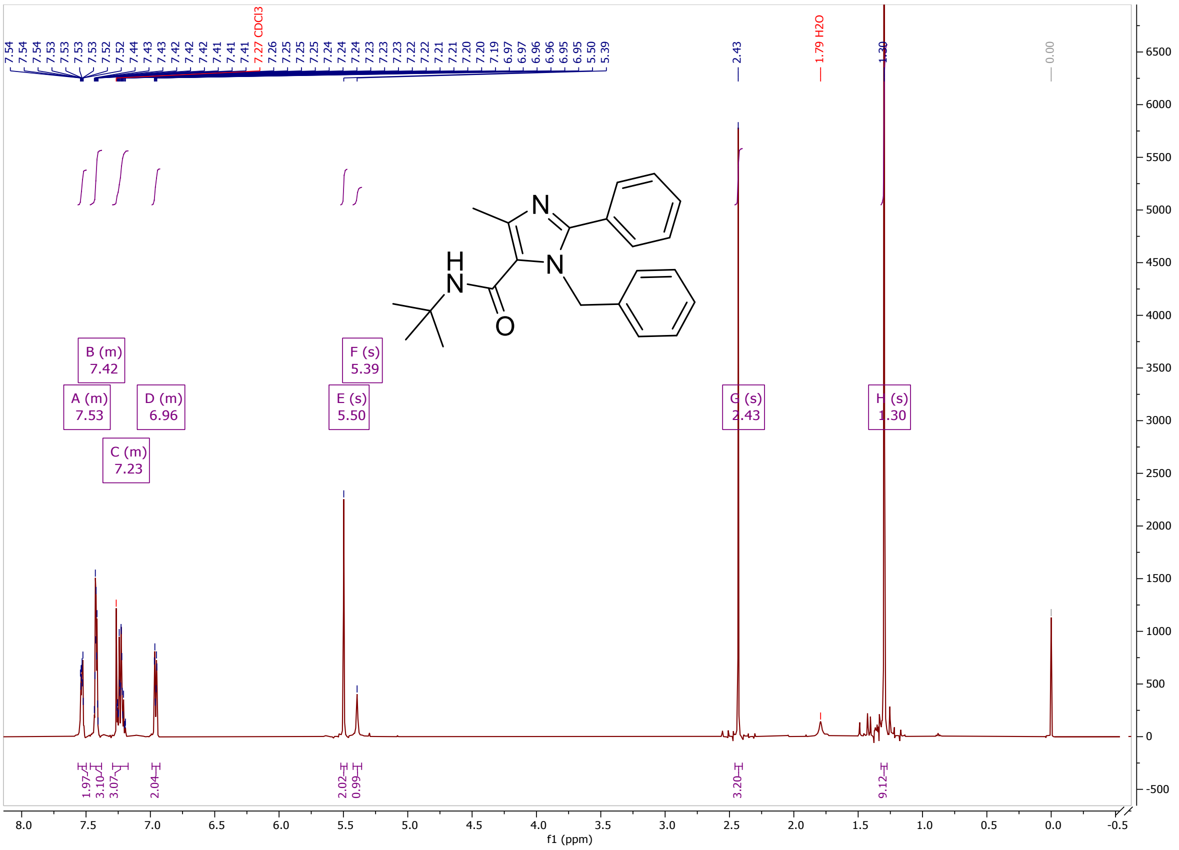

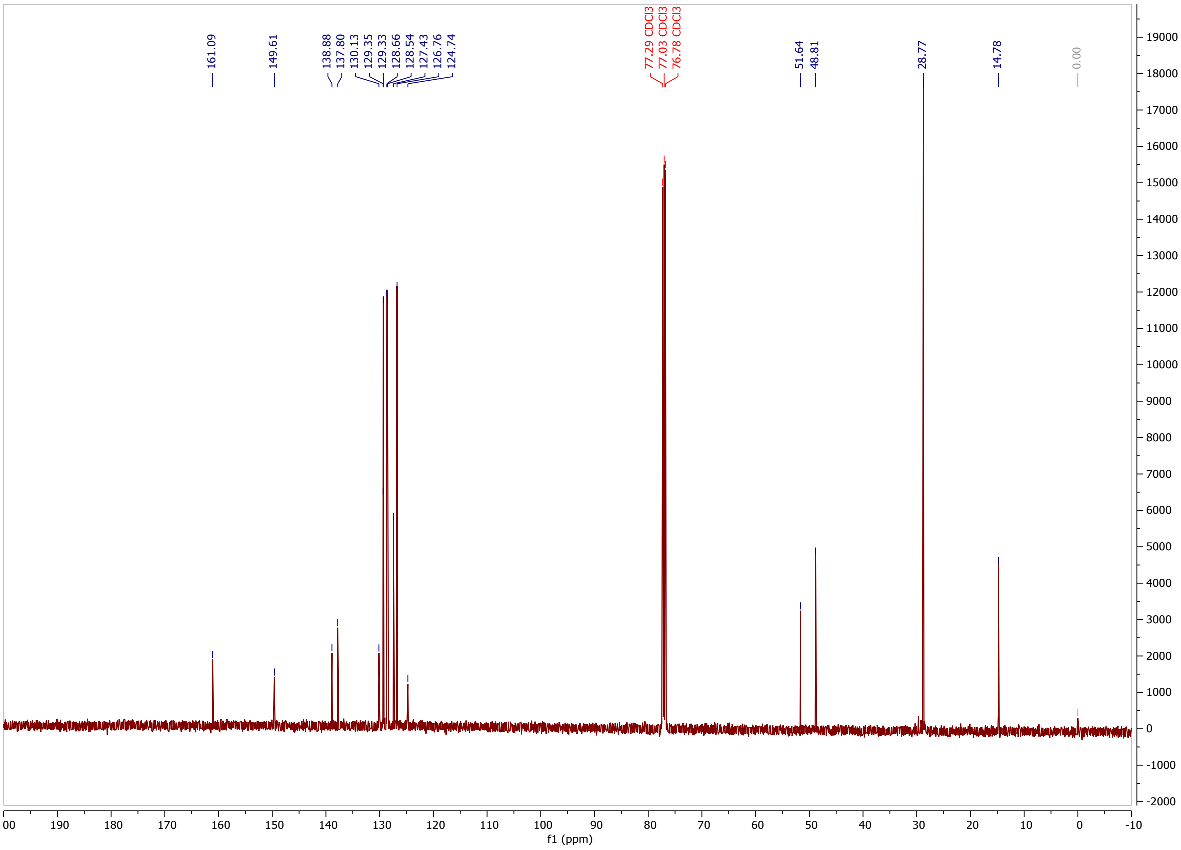

^{1}H and ^{13}C NMR spectra of compound 6d

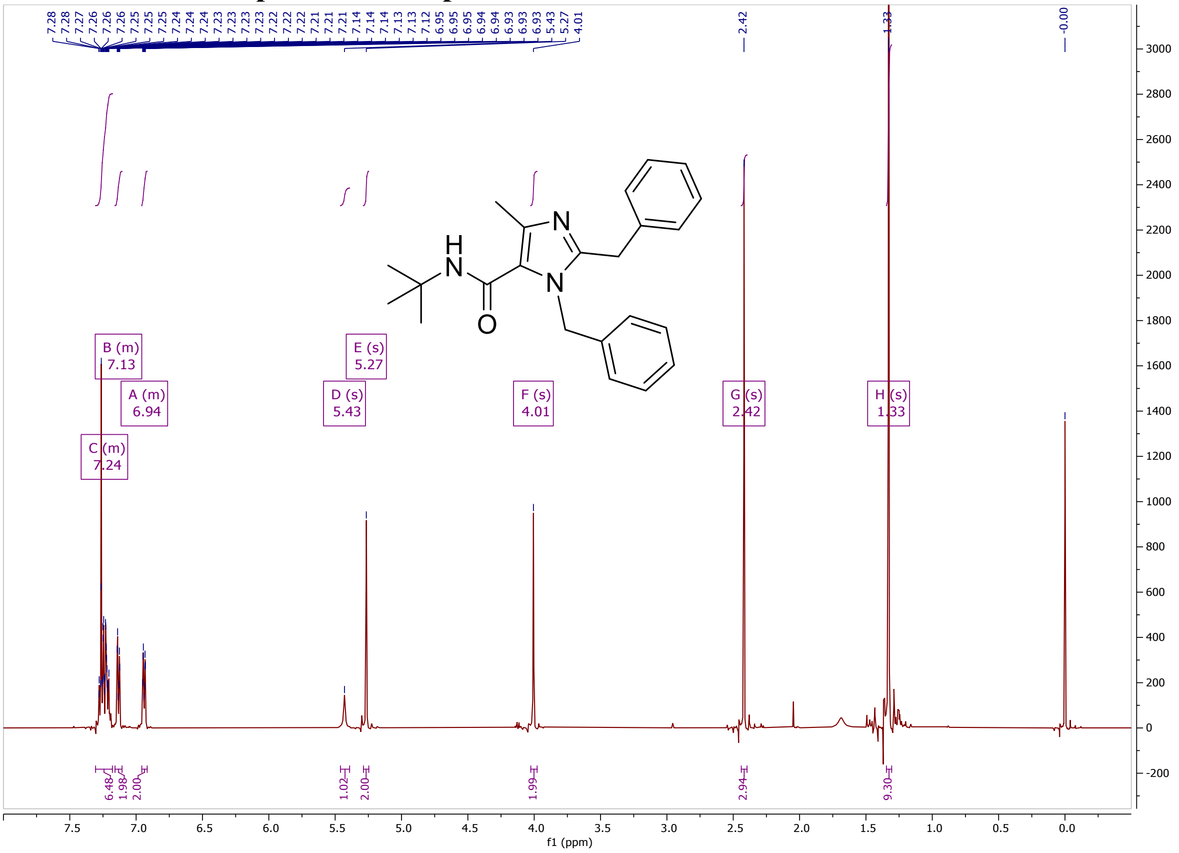

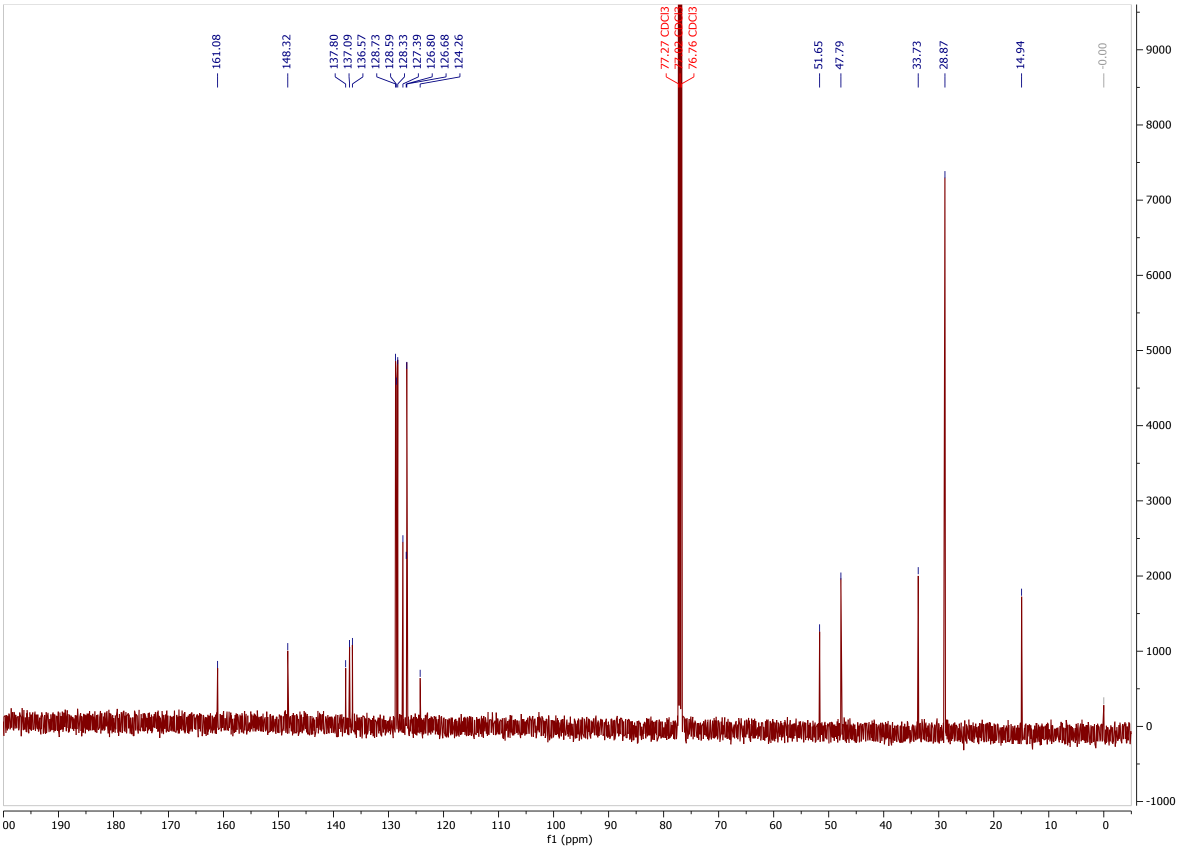

^{1}H and ^{13}C NMR spectra of compound 6e

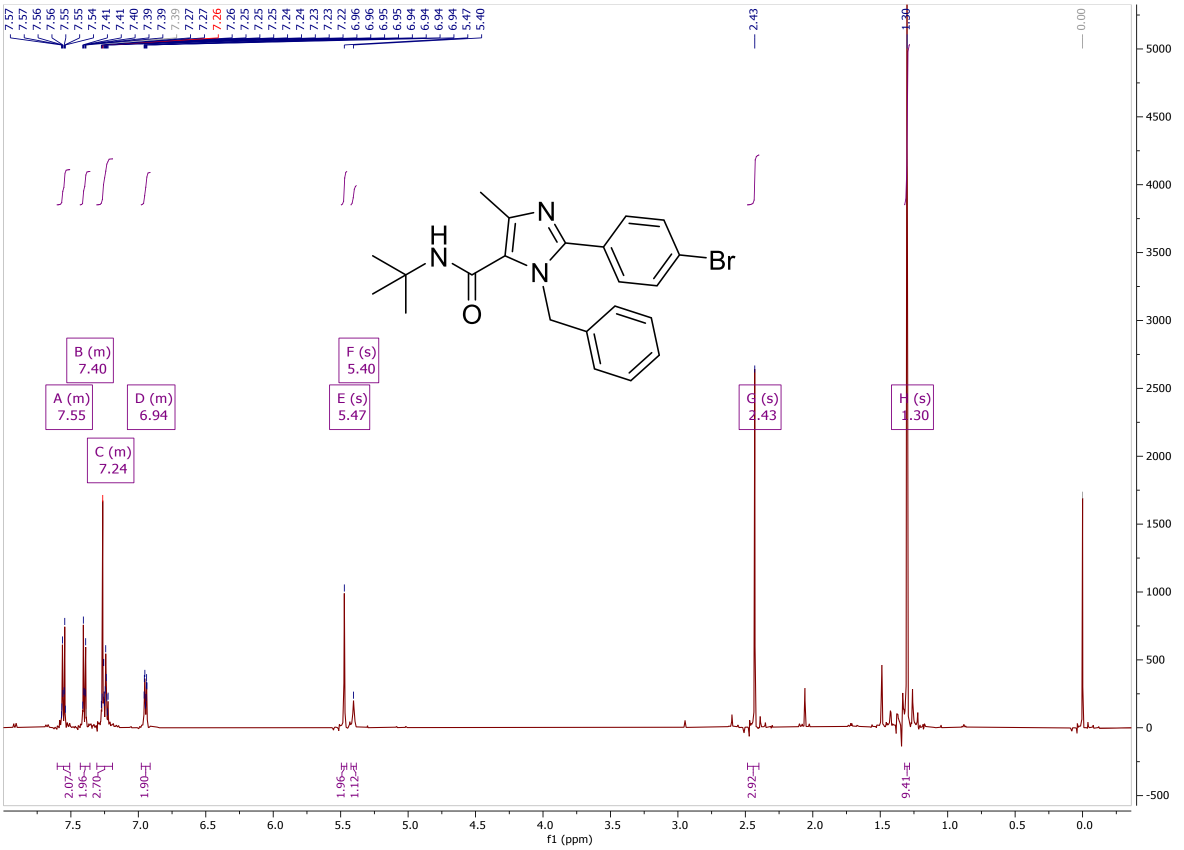

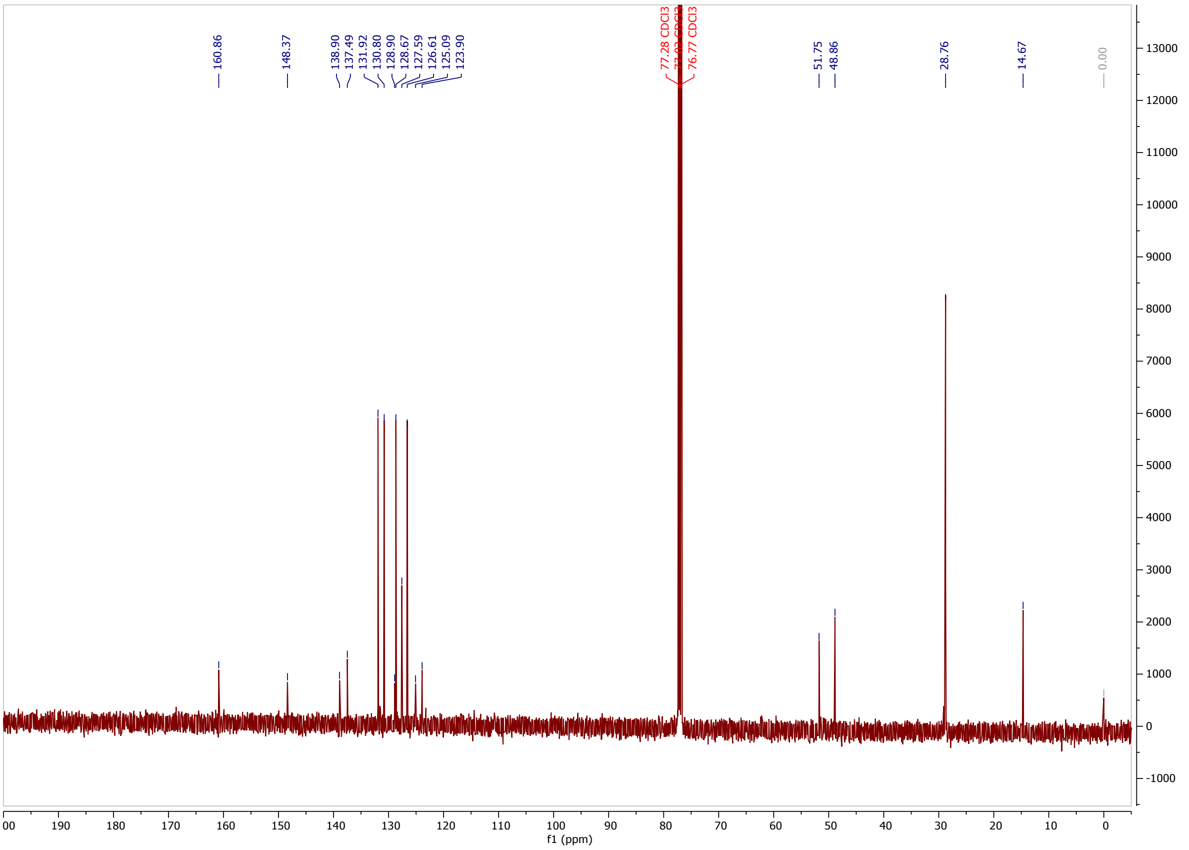

^{1}H and ^{13}C NMR spectra of compound 6f

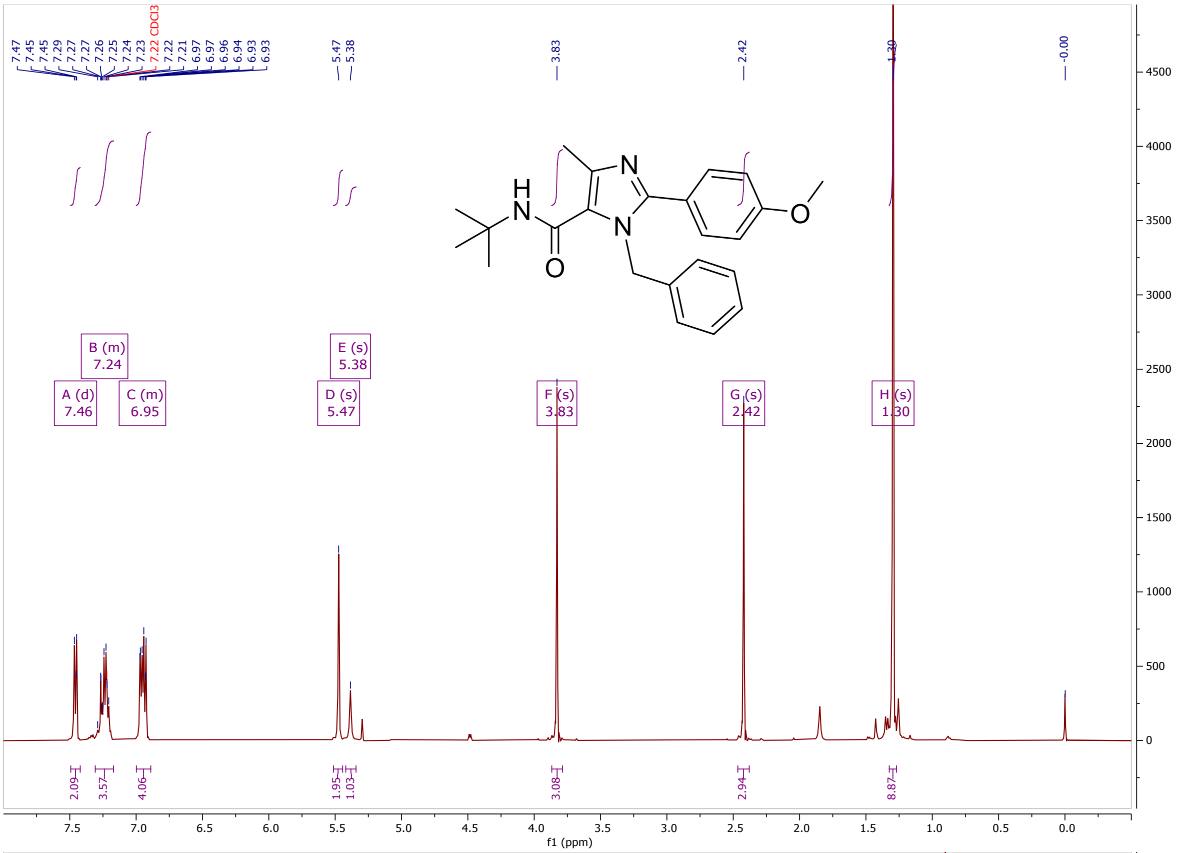

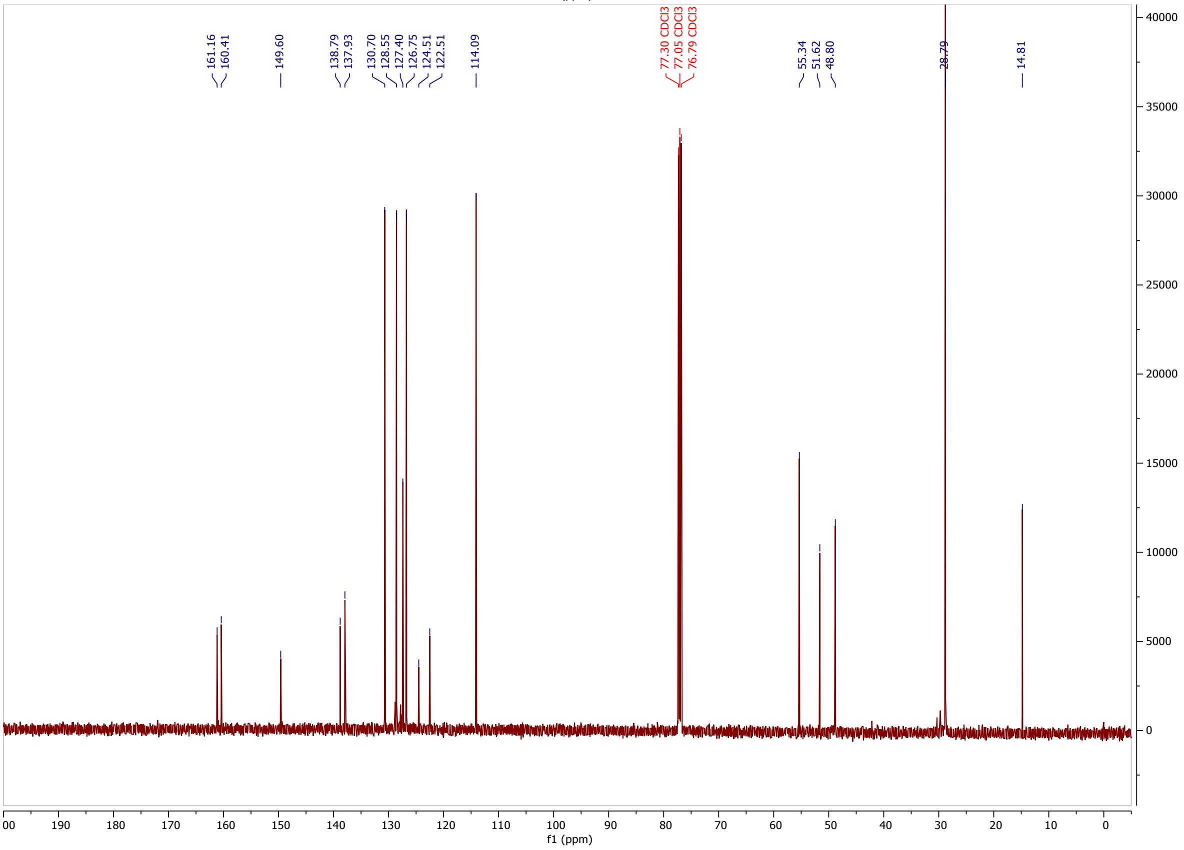

^{1}H and ^{13}C NMR spectra of compound 6g

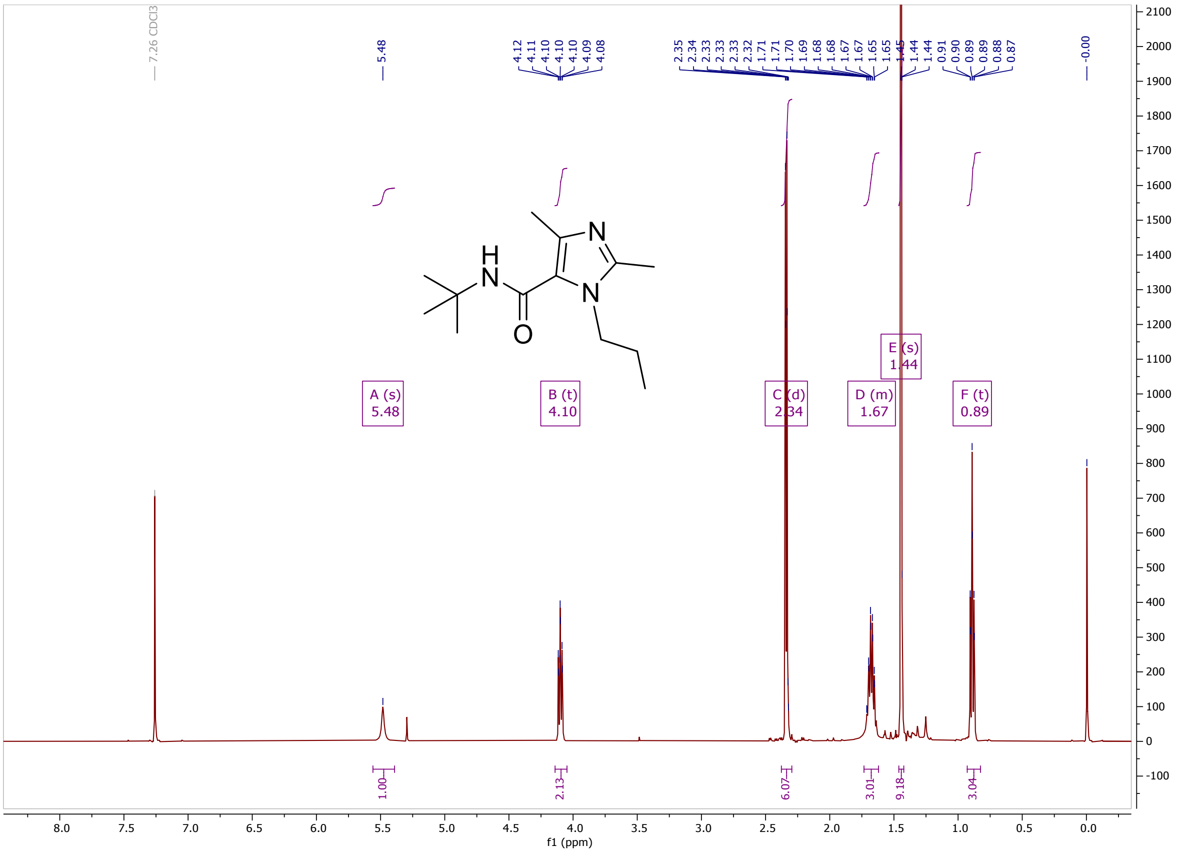

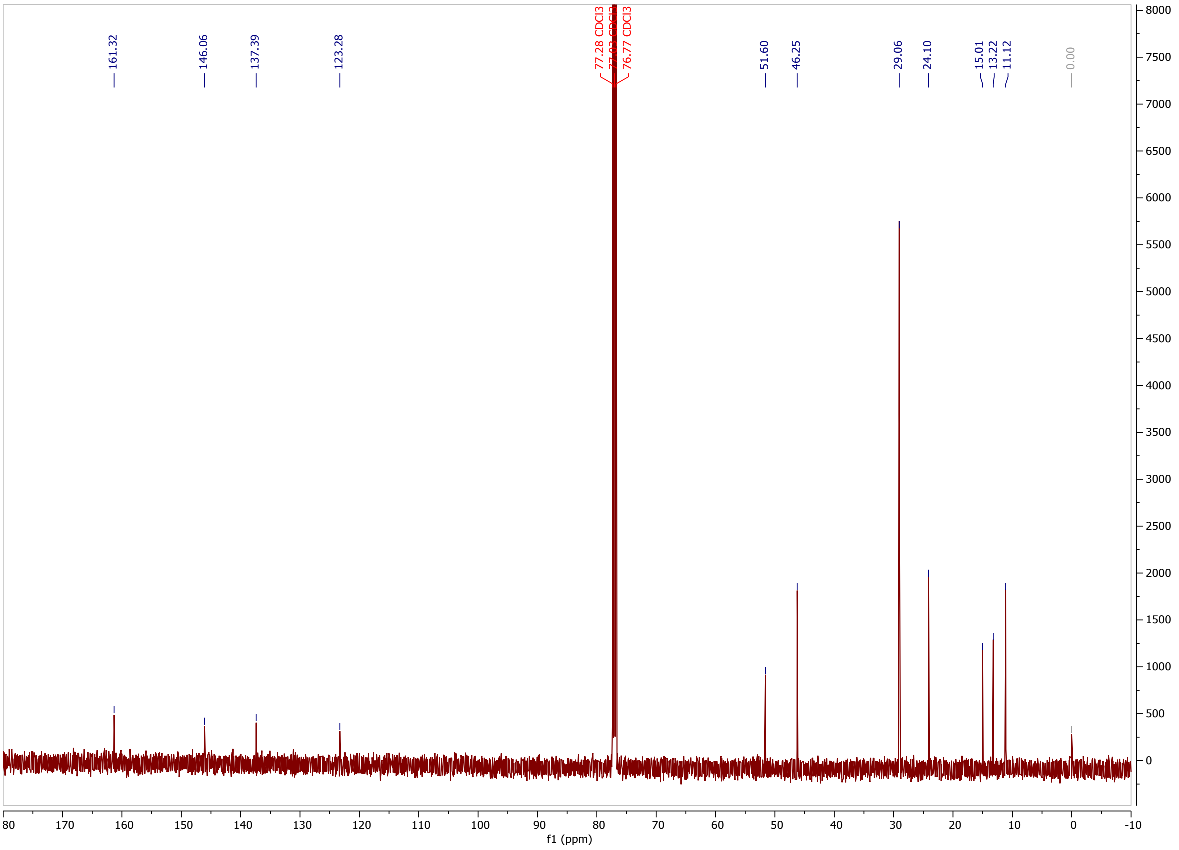

^{1}H and ^{13}C NMR spectra of compound 6h

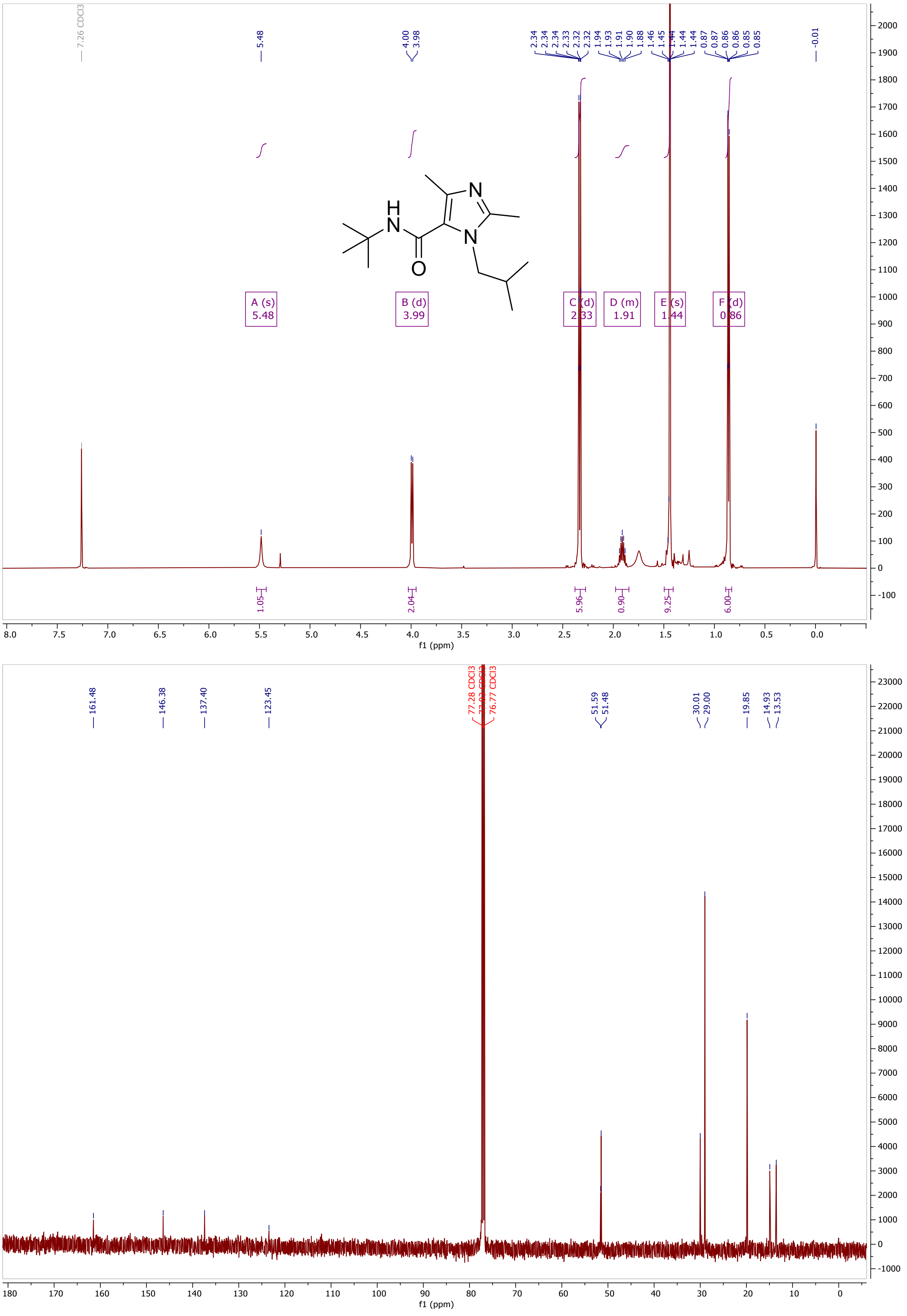

^{1}H and ^{13}C NMR spectra of compound 6i

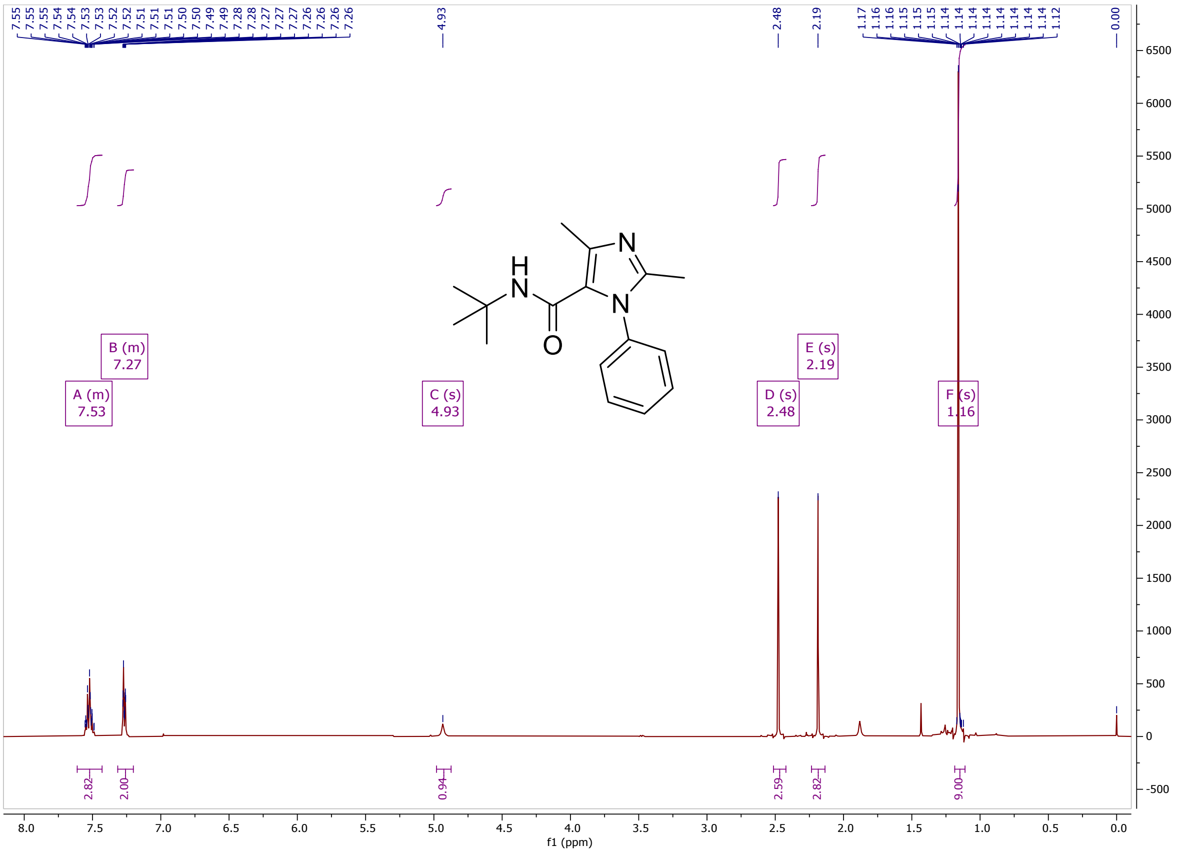

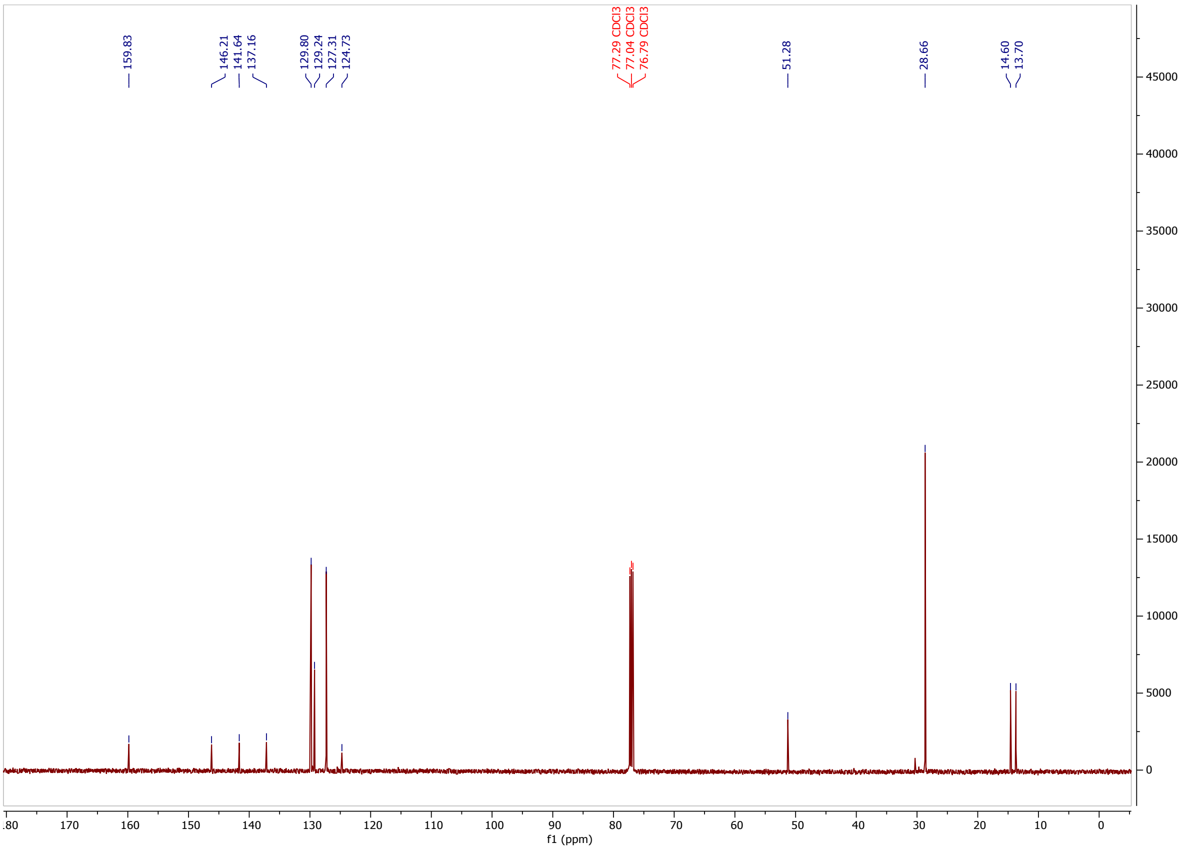

^{1}H and ^{13}C NMR spectra of compound 6j

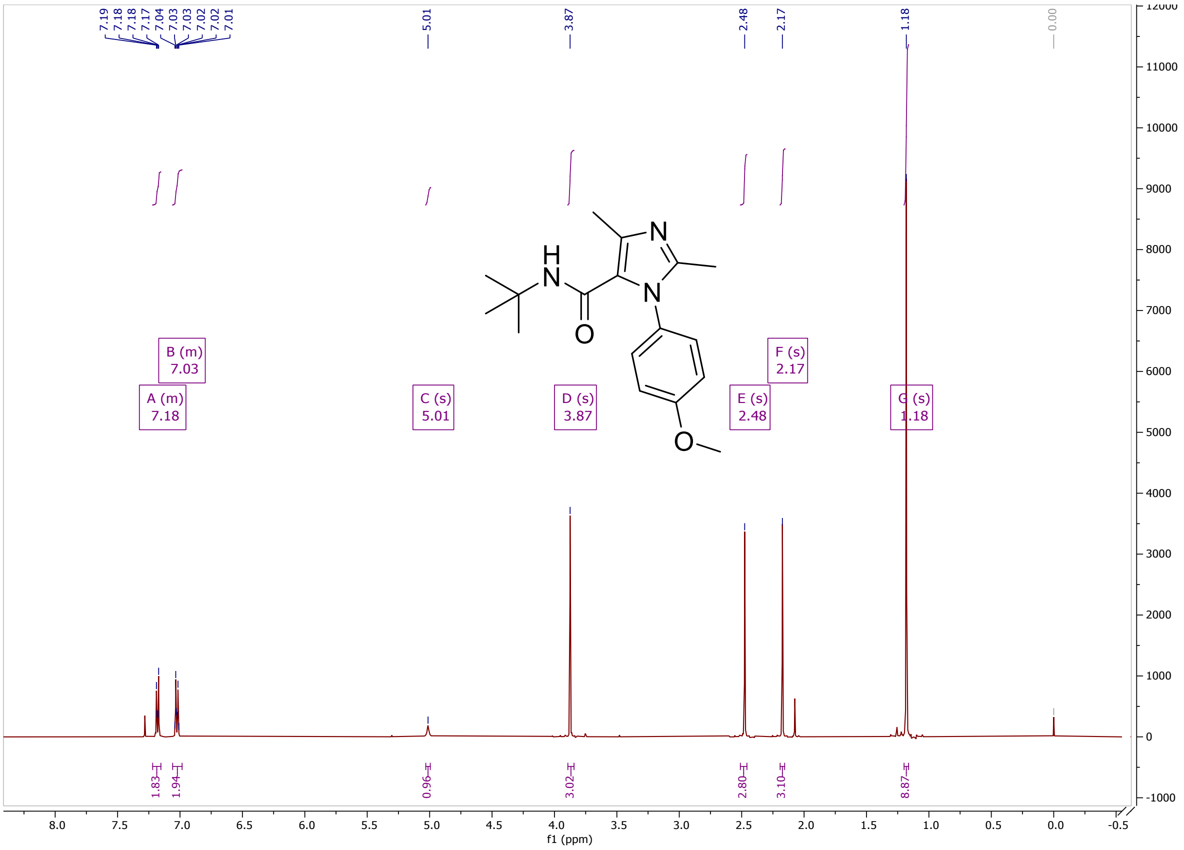

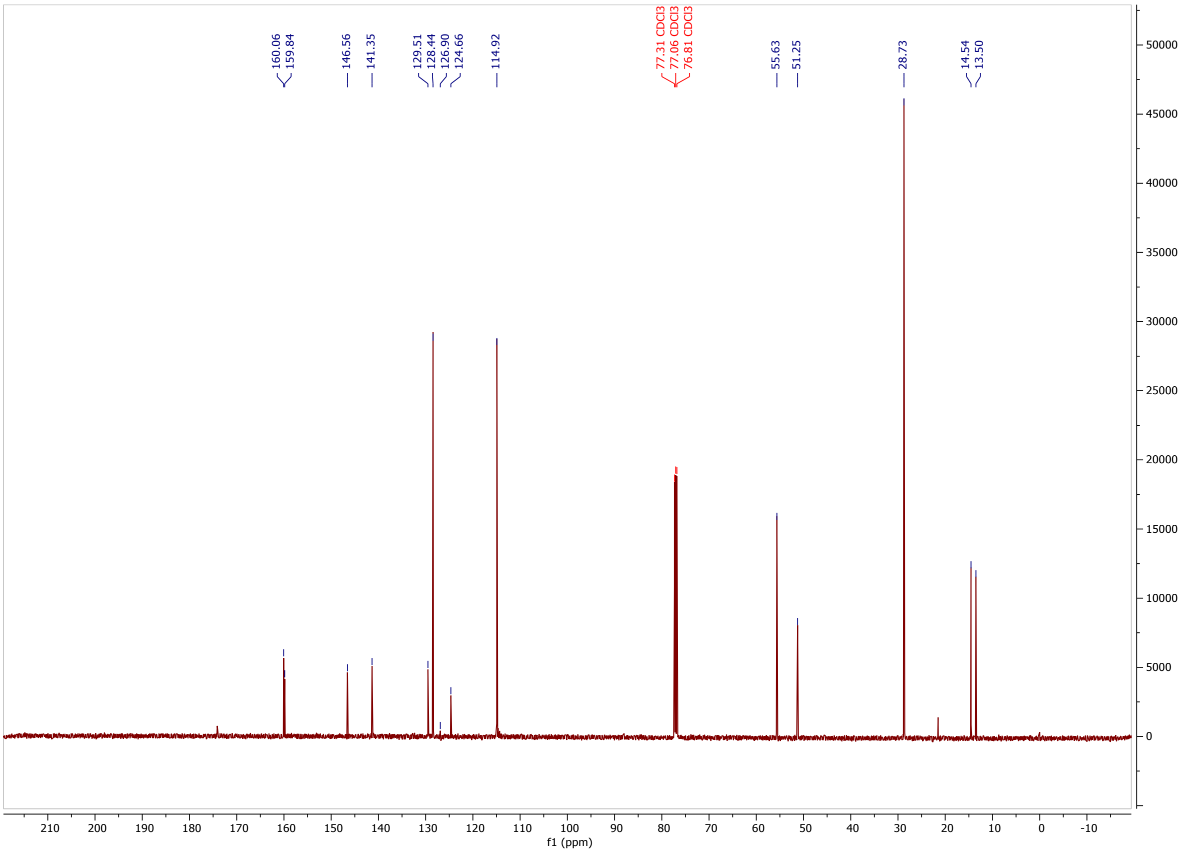

^1H and ^{13}C NMR spectra of compound 6k

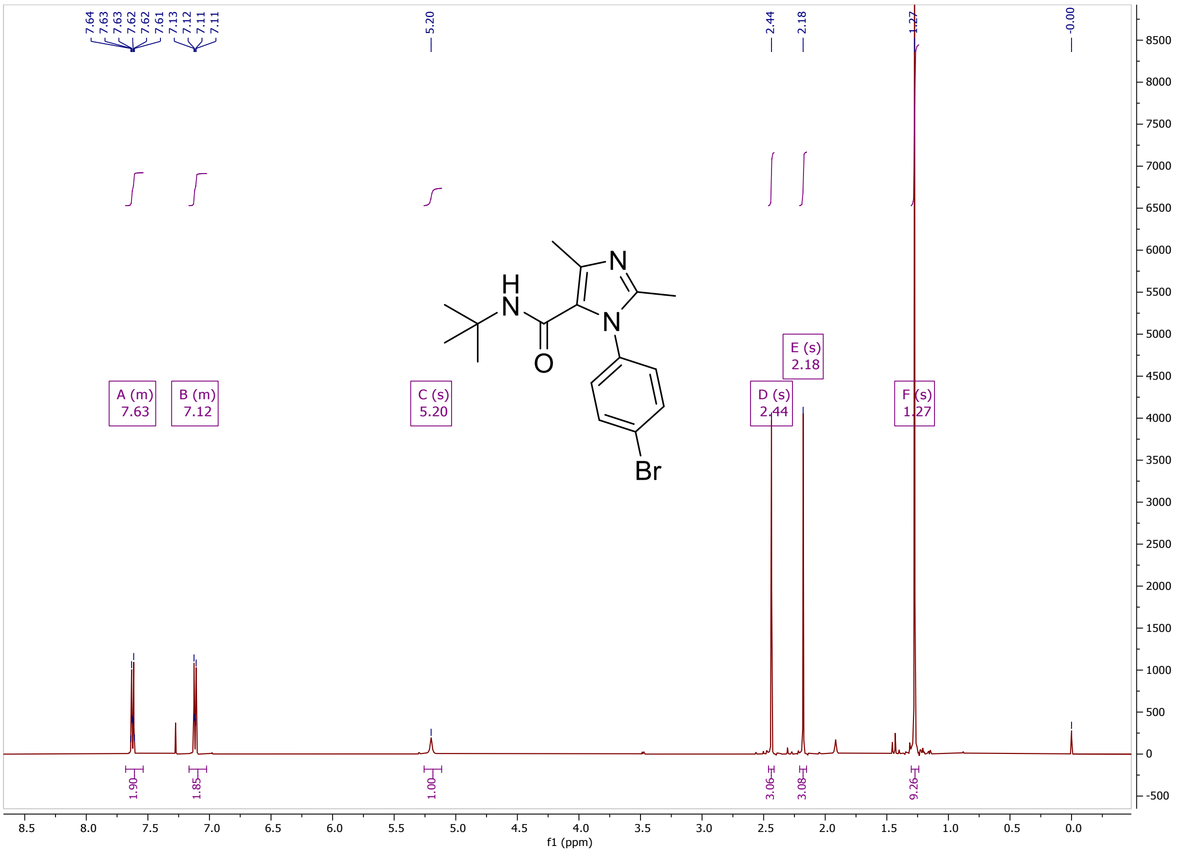

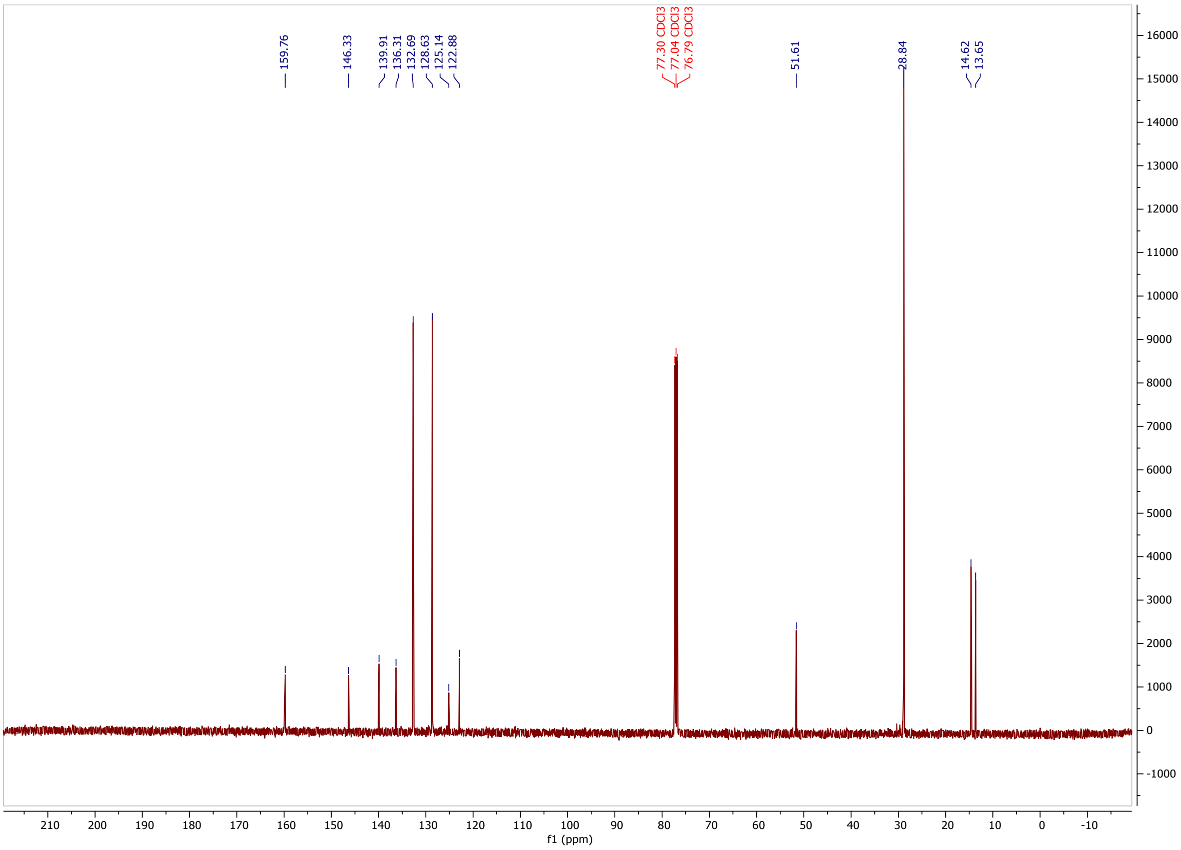

^{1}H and ^{13}C NMR spectra of compound 6l

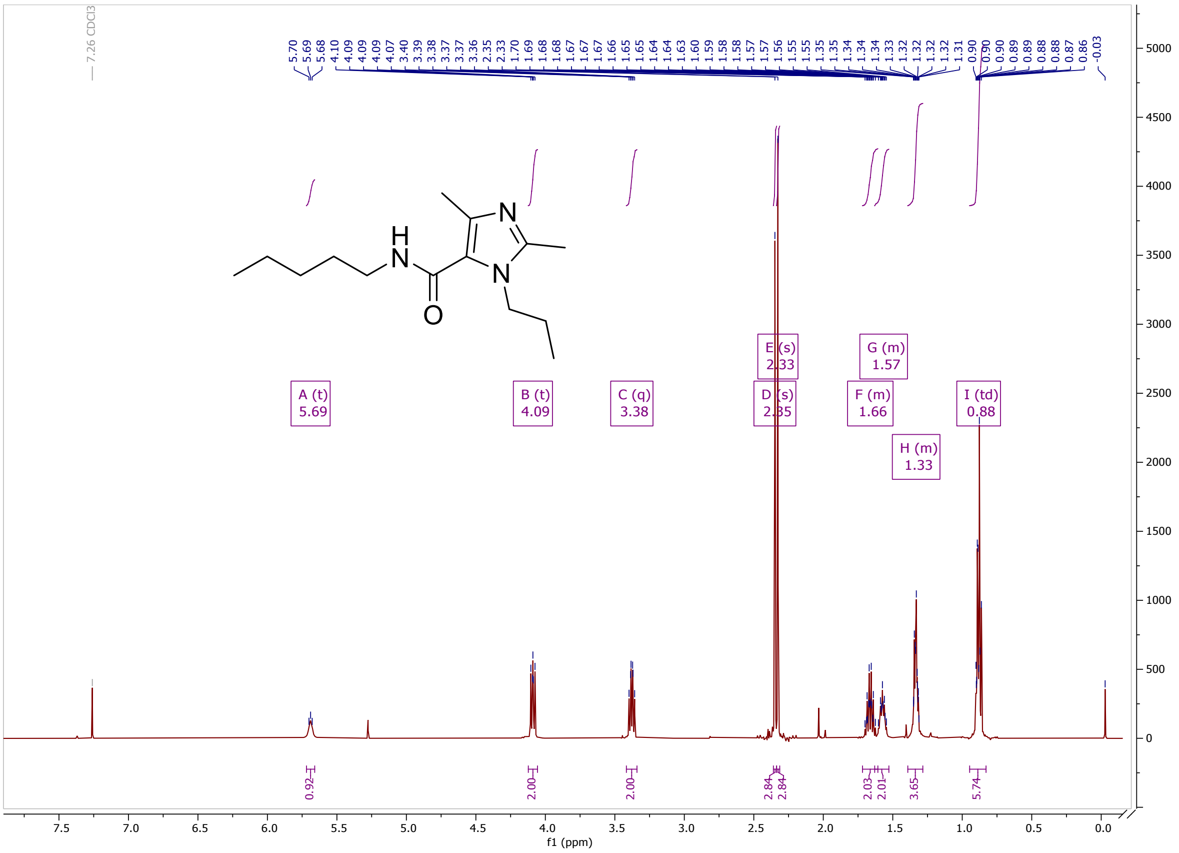

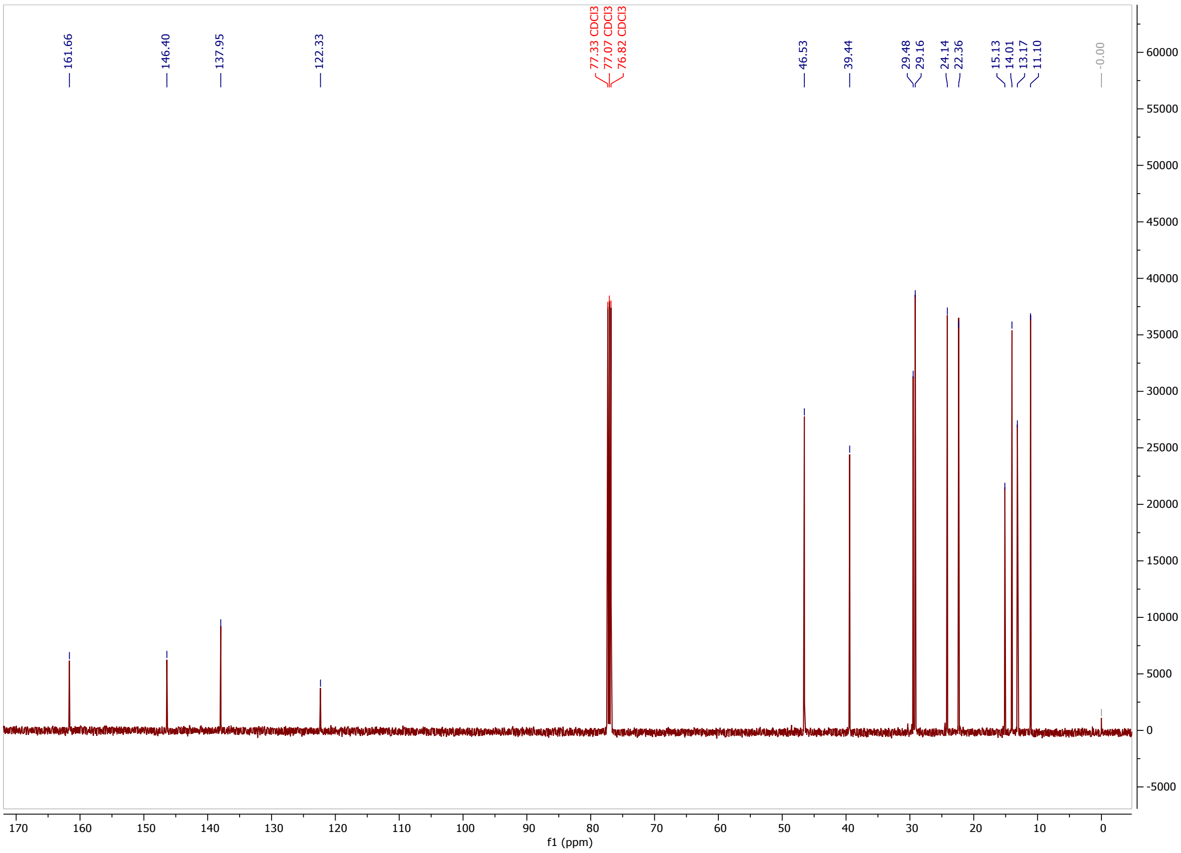

^{1}H and ^{13}C NMR spectra of compound 6m

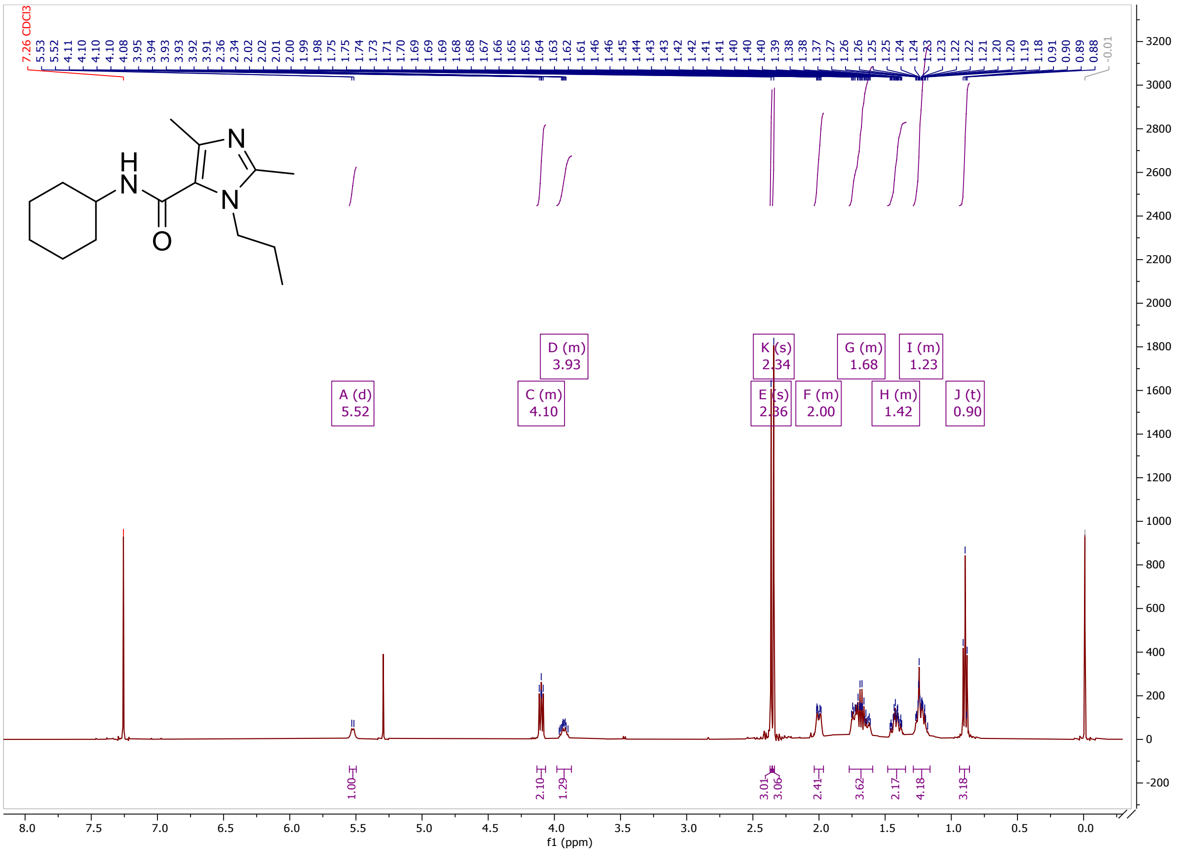

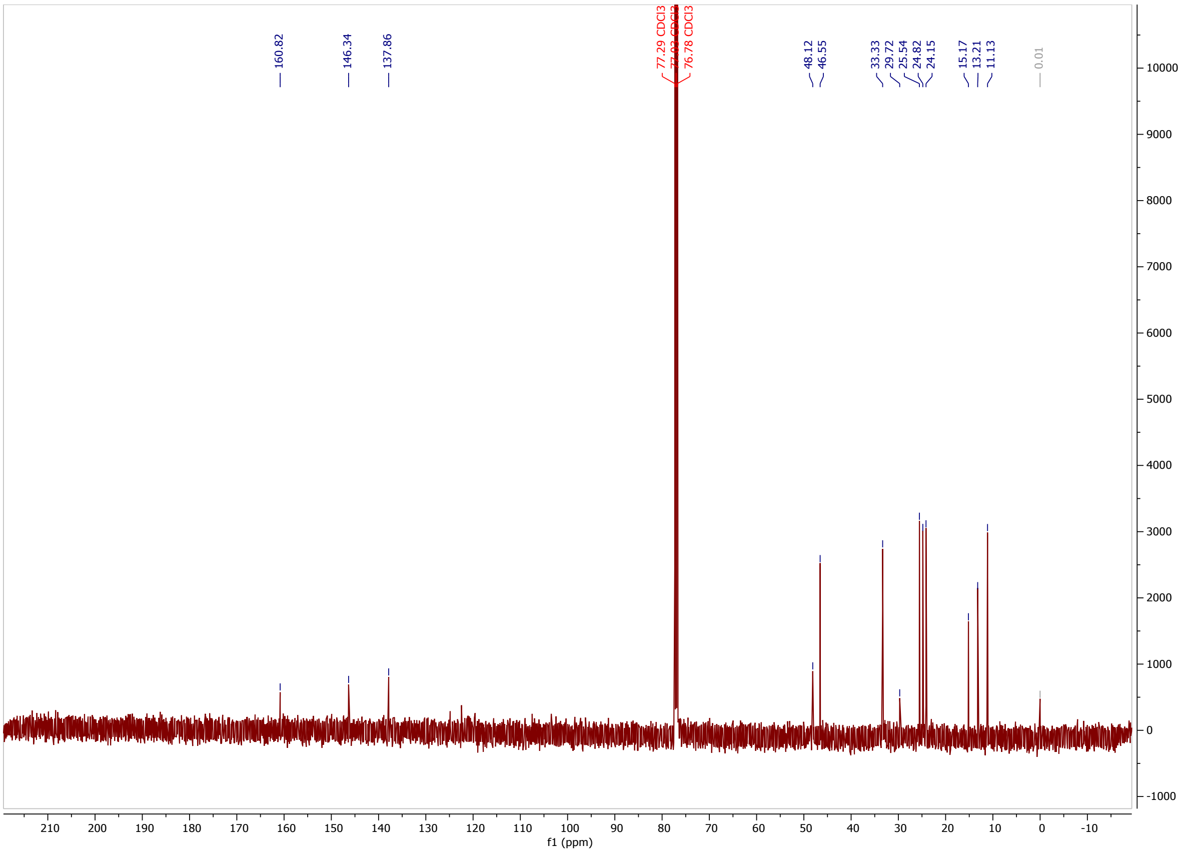

^{1}H and ^{13}C NMR spectra of compound 6n

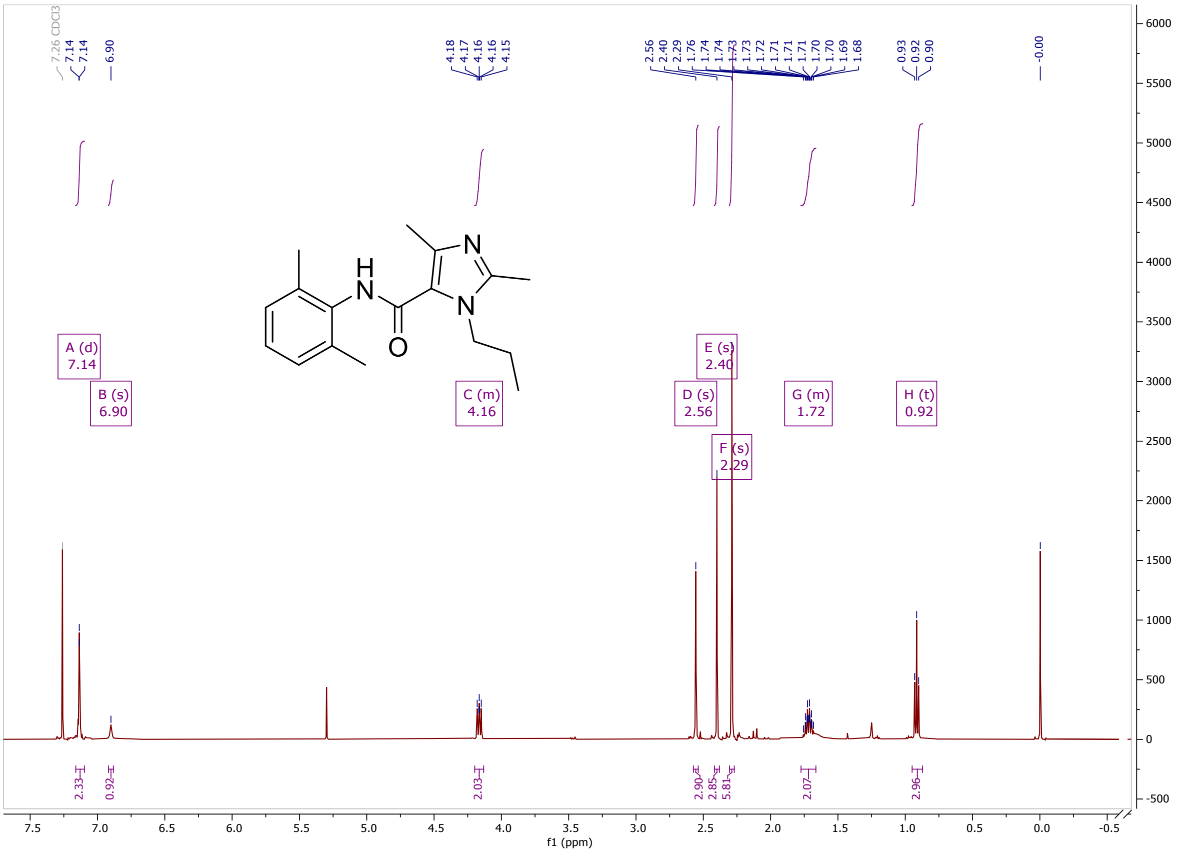

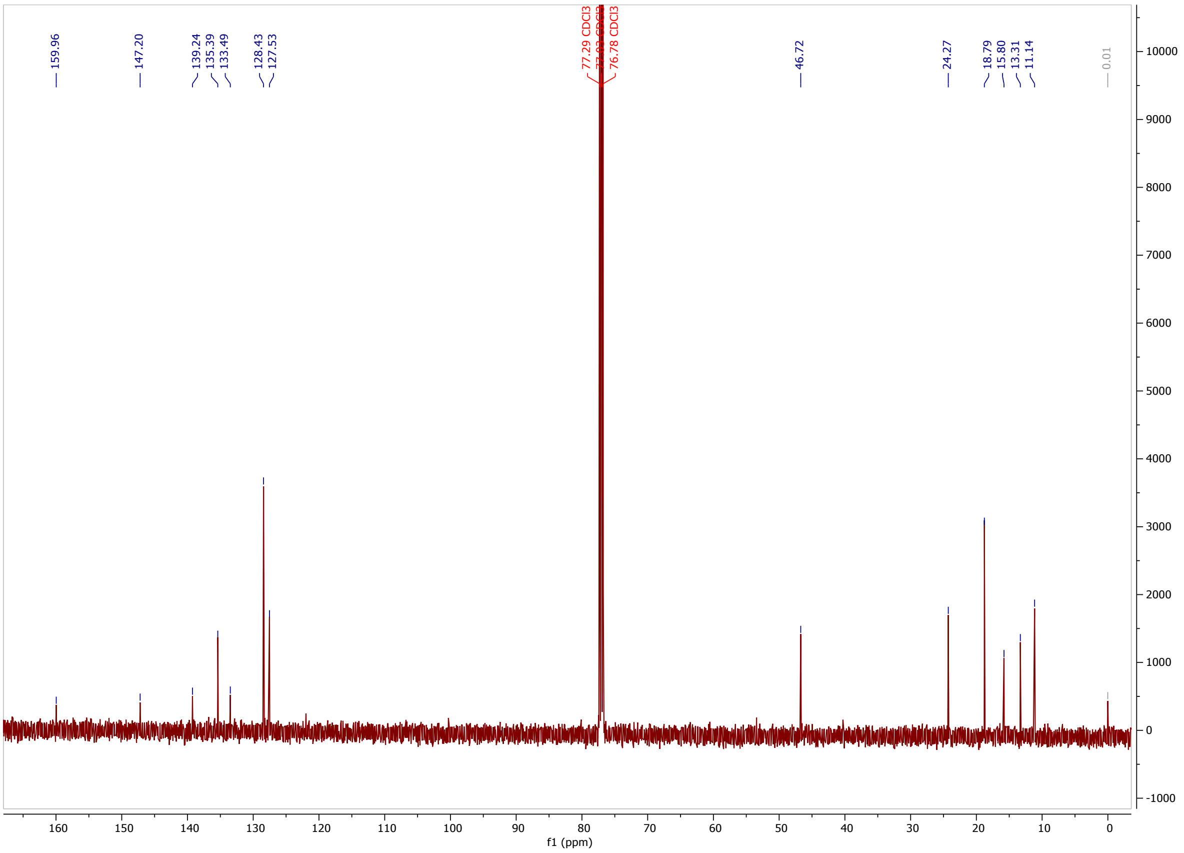

^{1}H and ^{13}C NMR spectra of compound 6o

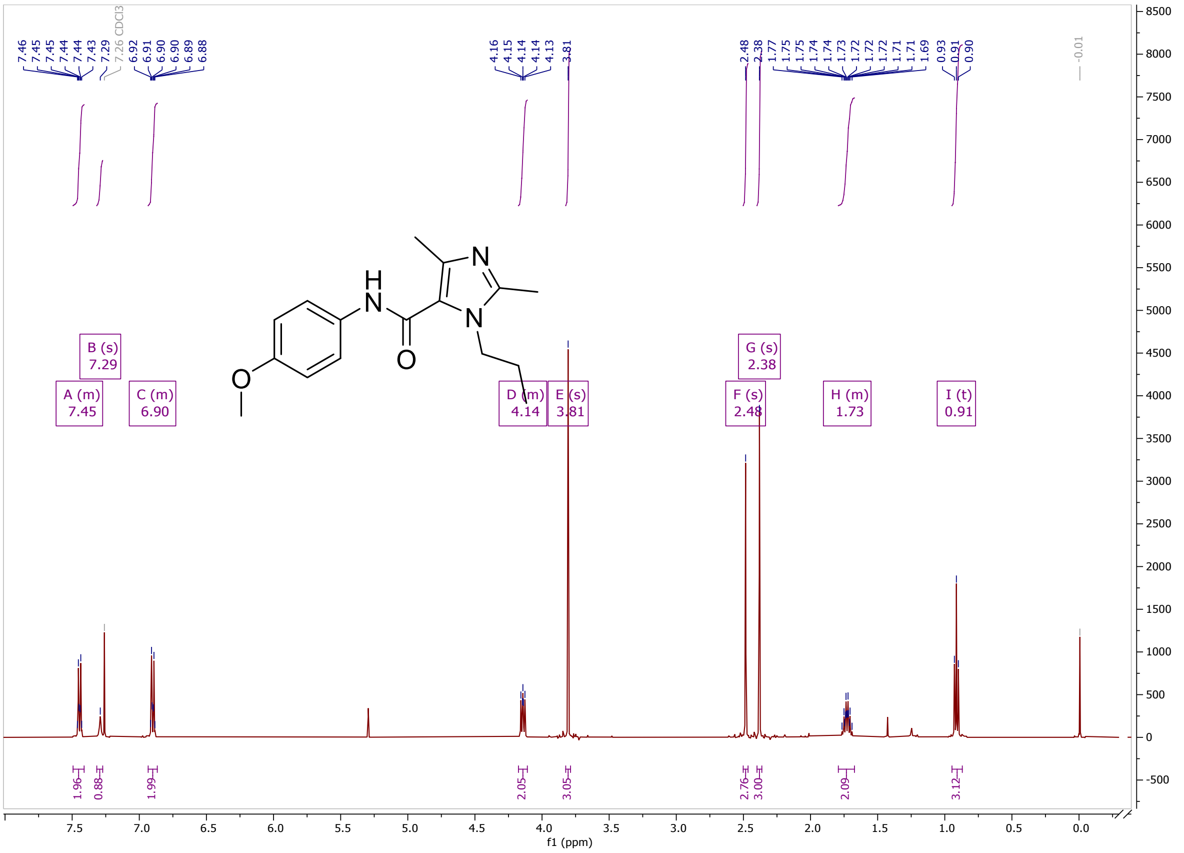

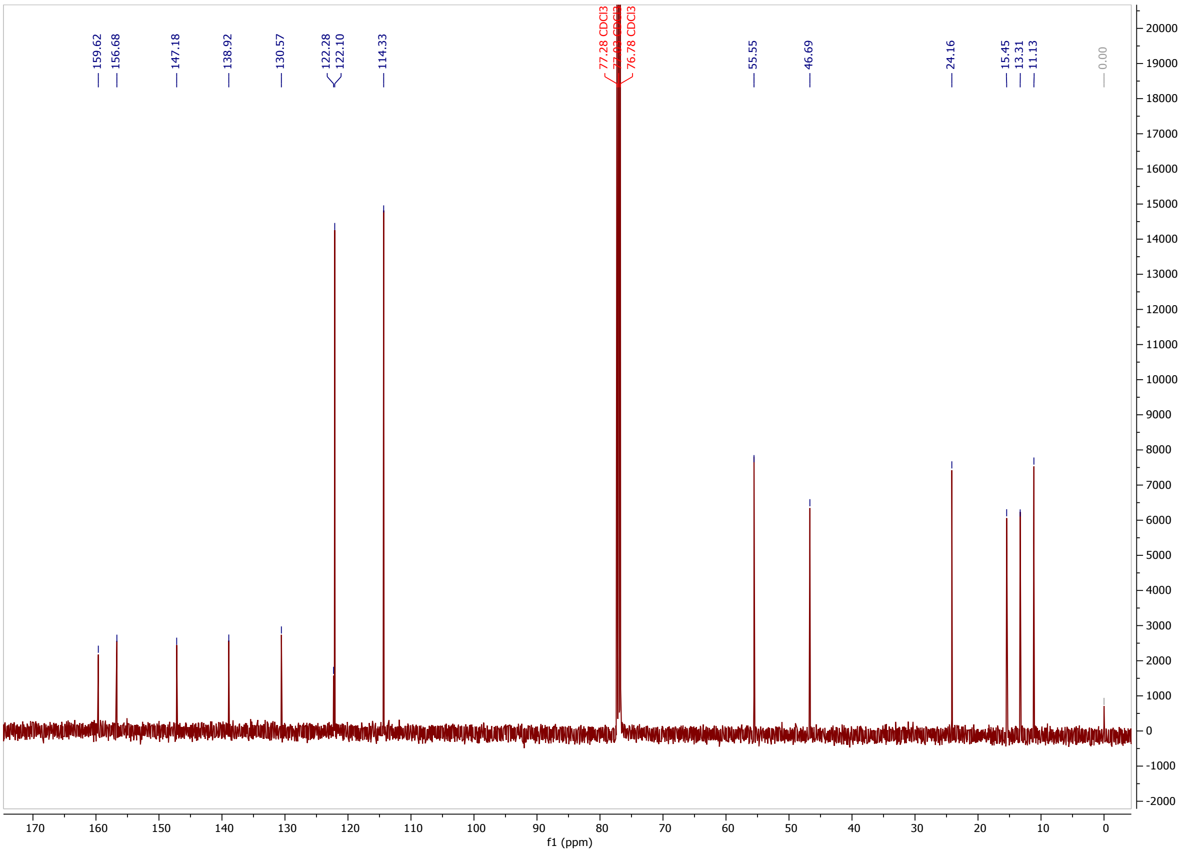

^{1}H and ^{13}C NMR spectra of compound 6p

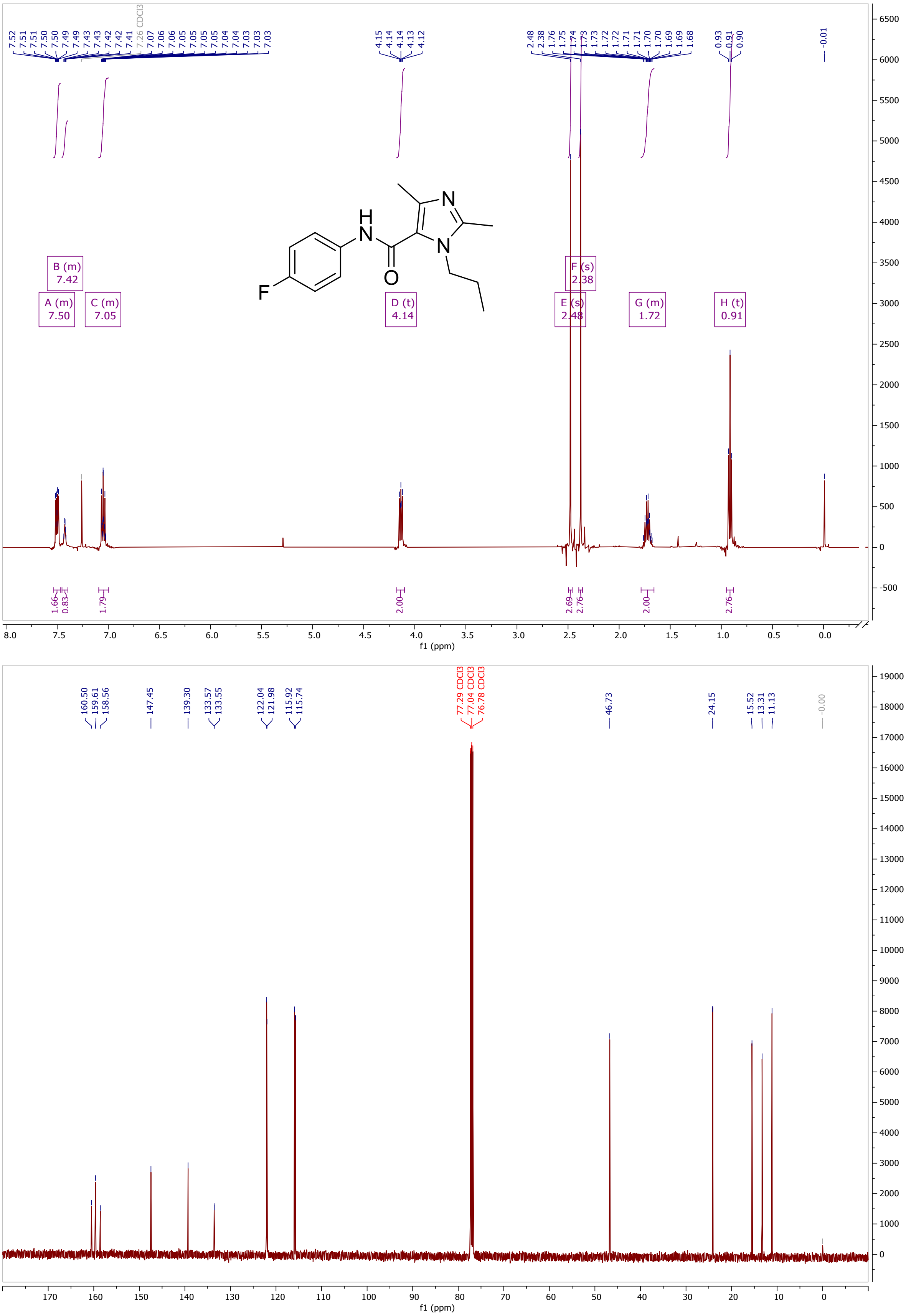

^{1}H and ^{13}C NMR spectra of compound 6q

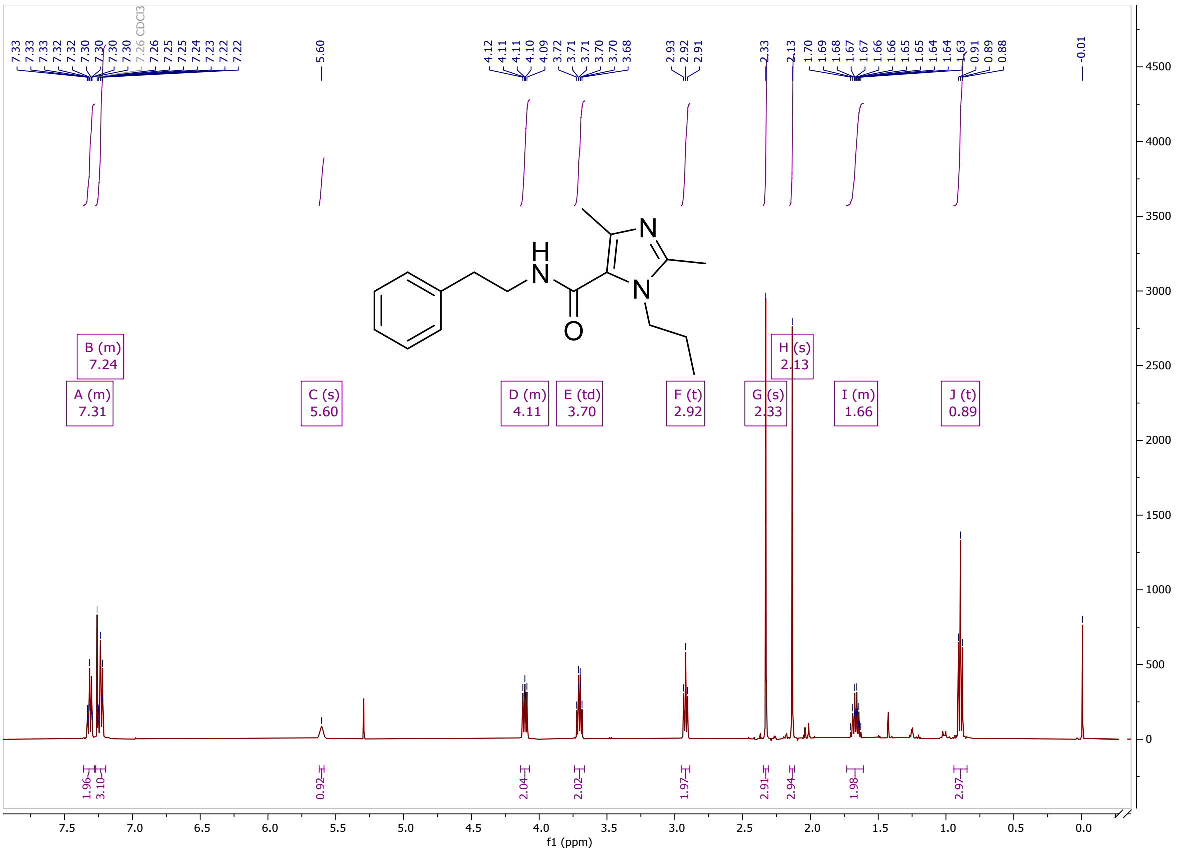

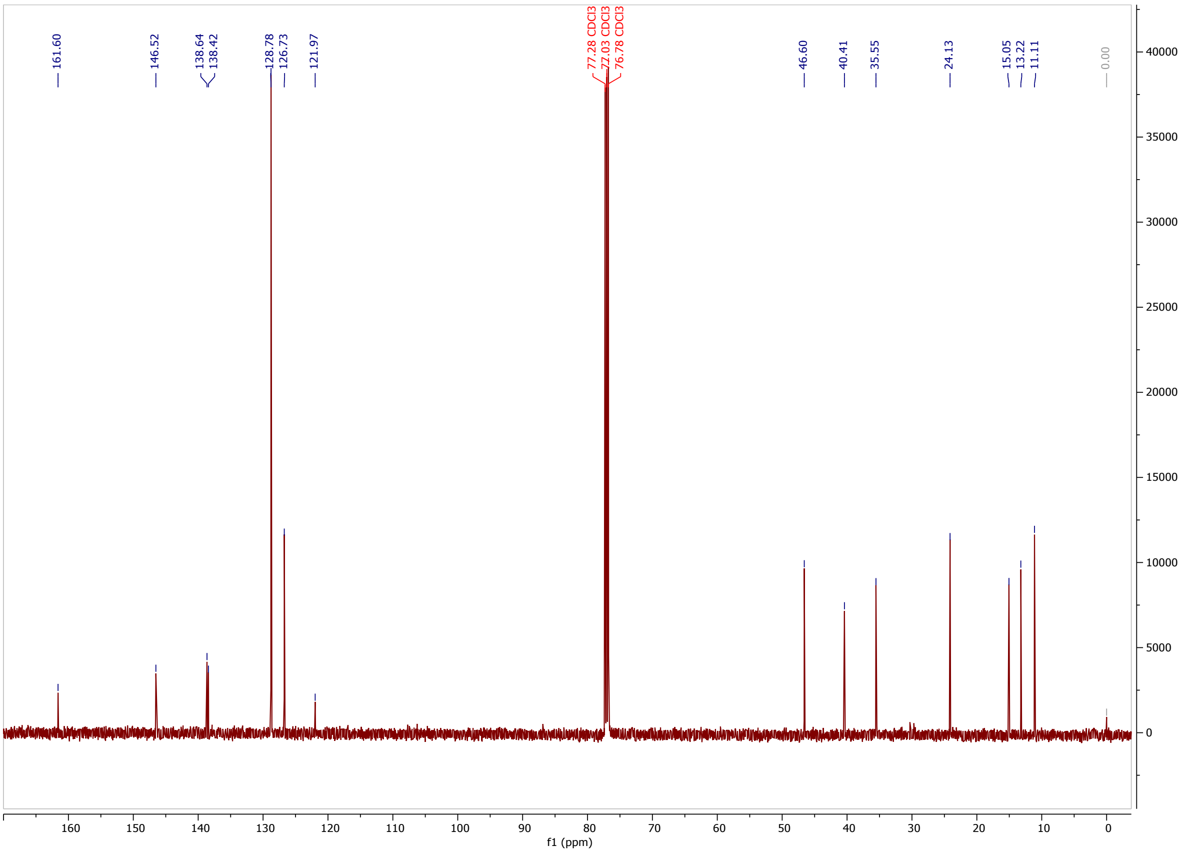

^{1}H and ^{13}C NMR spectra of compound 6r

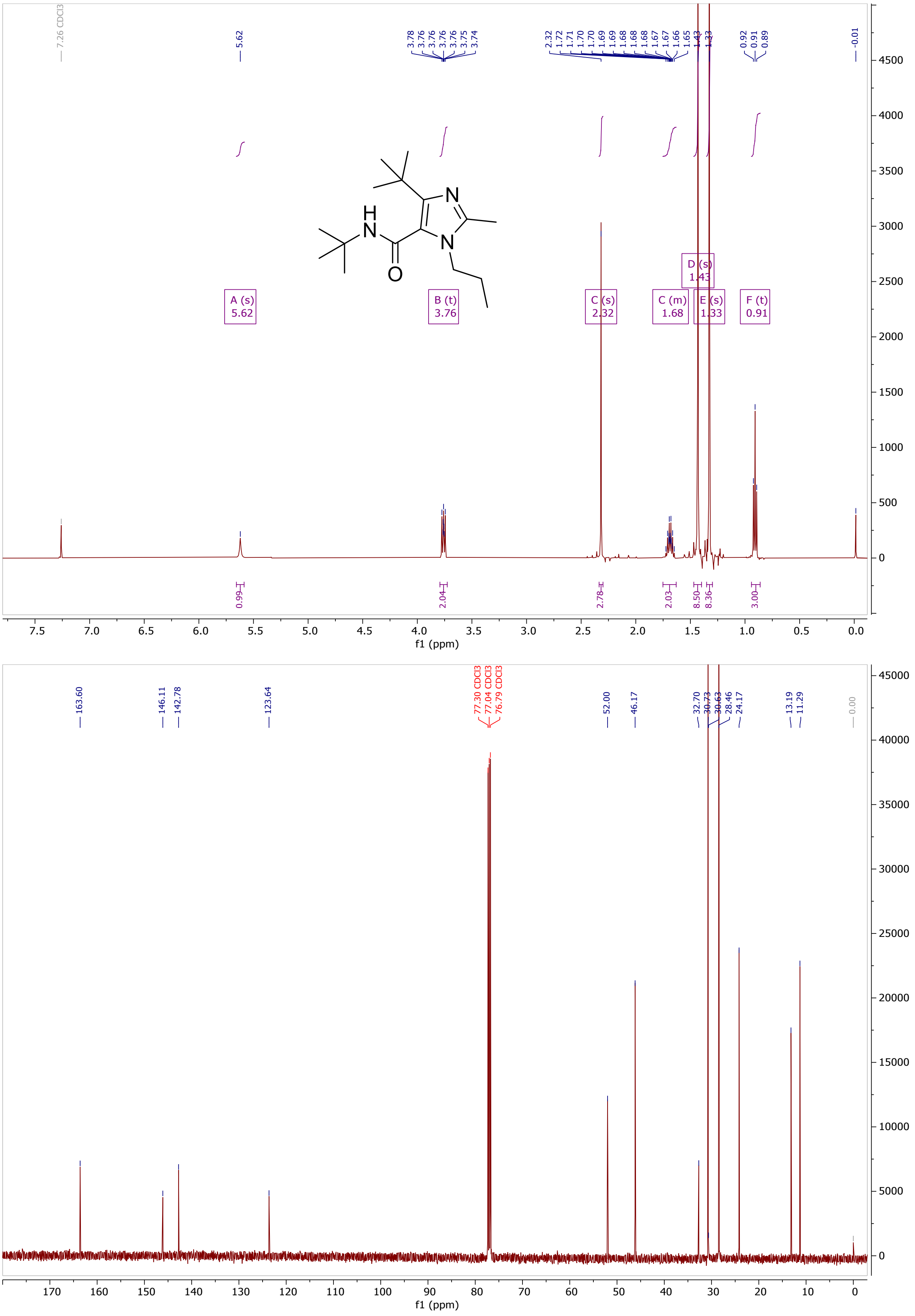

^{1}H and ^{13}C NMR spectra of compound 6s

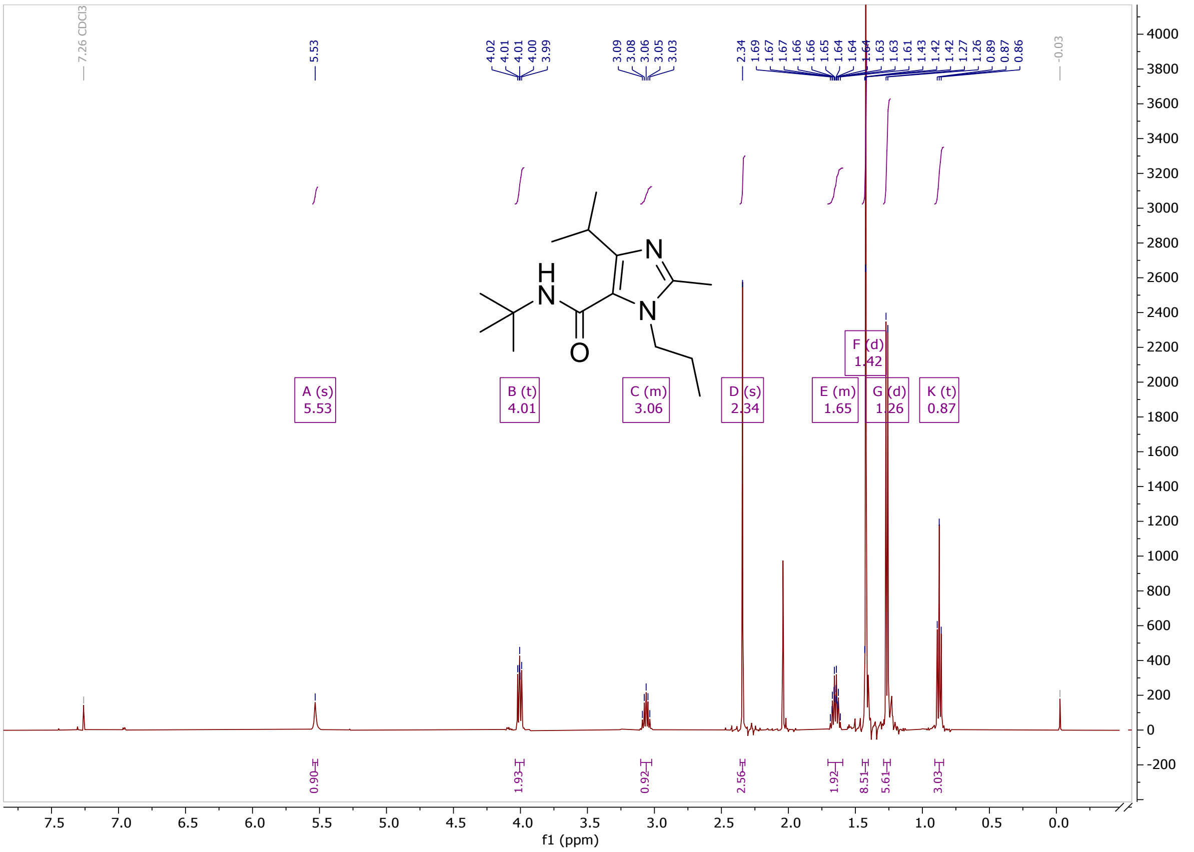

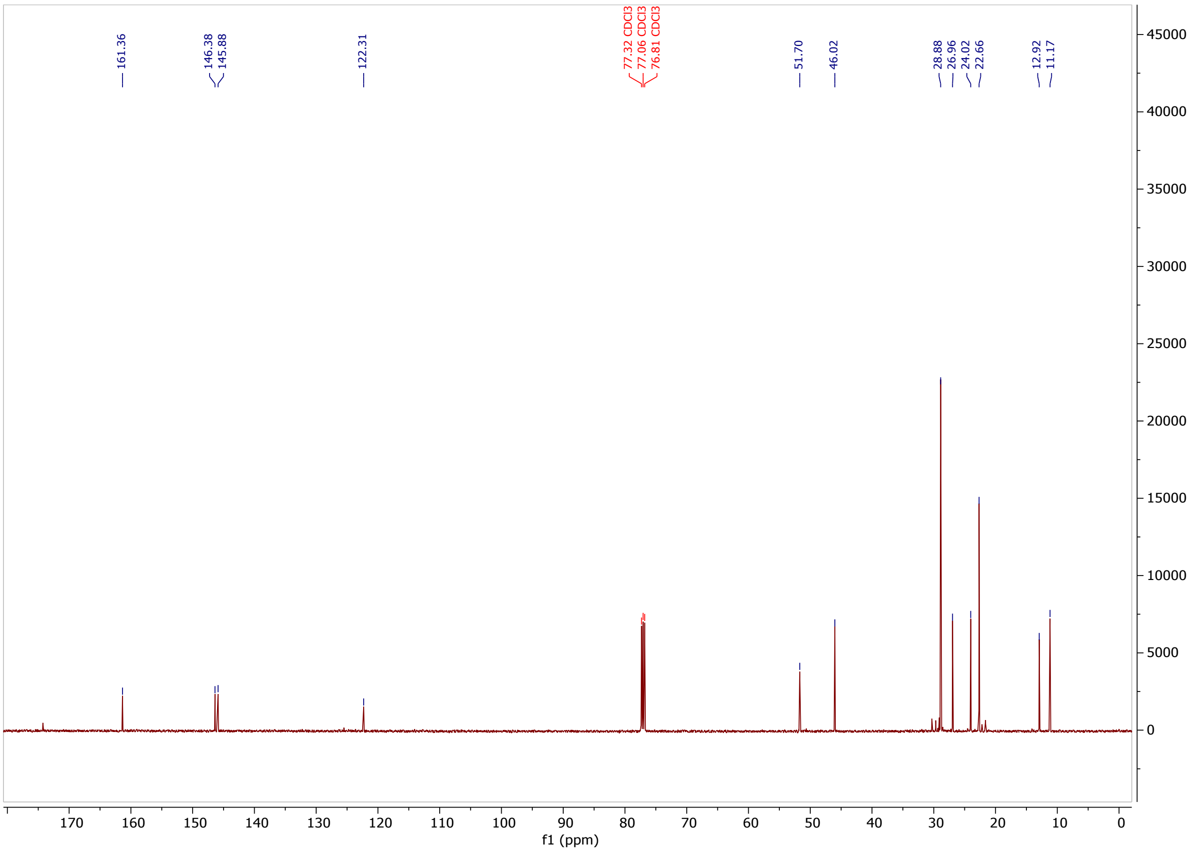

^{1}H and ^{13}C NMR spectra of compound 6t

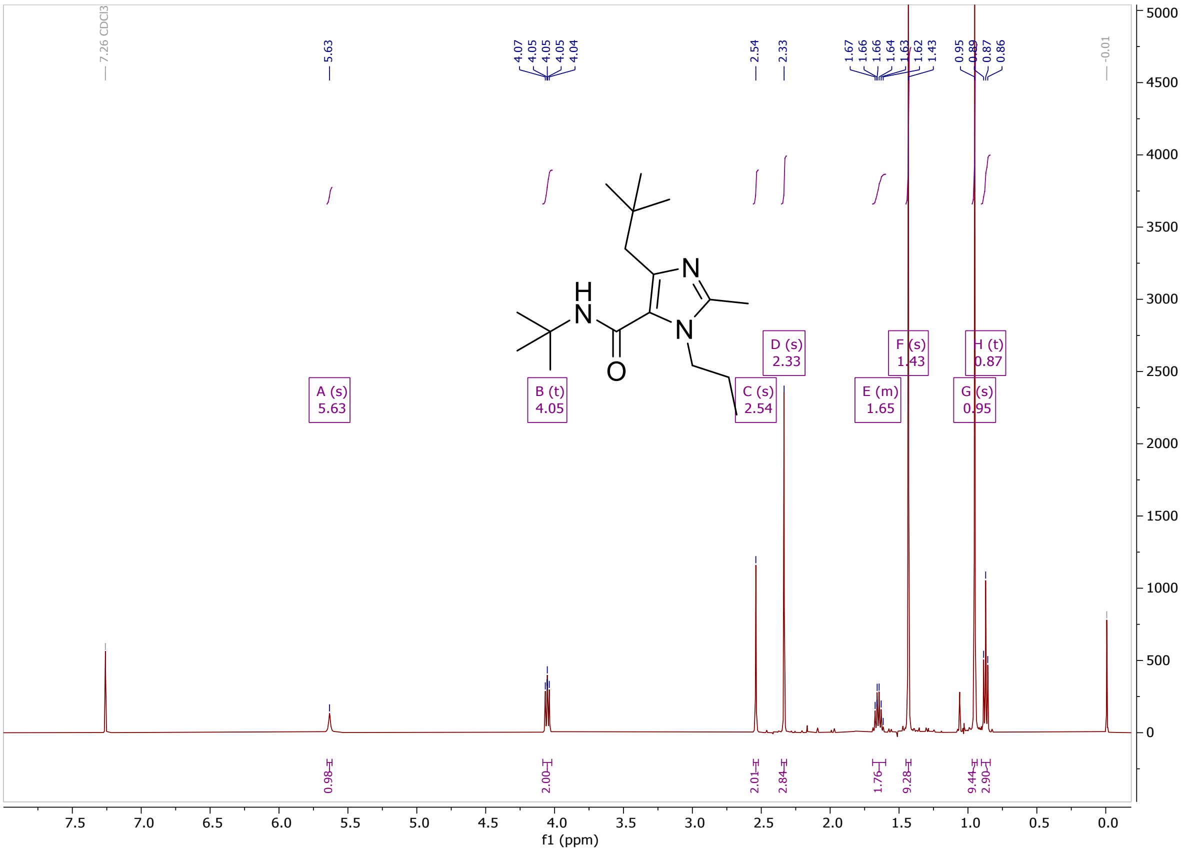

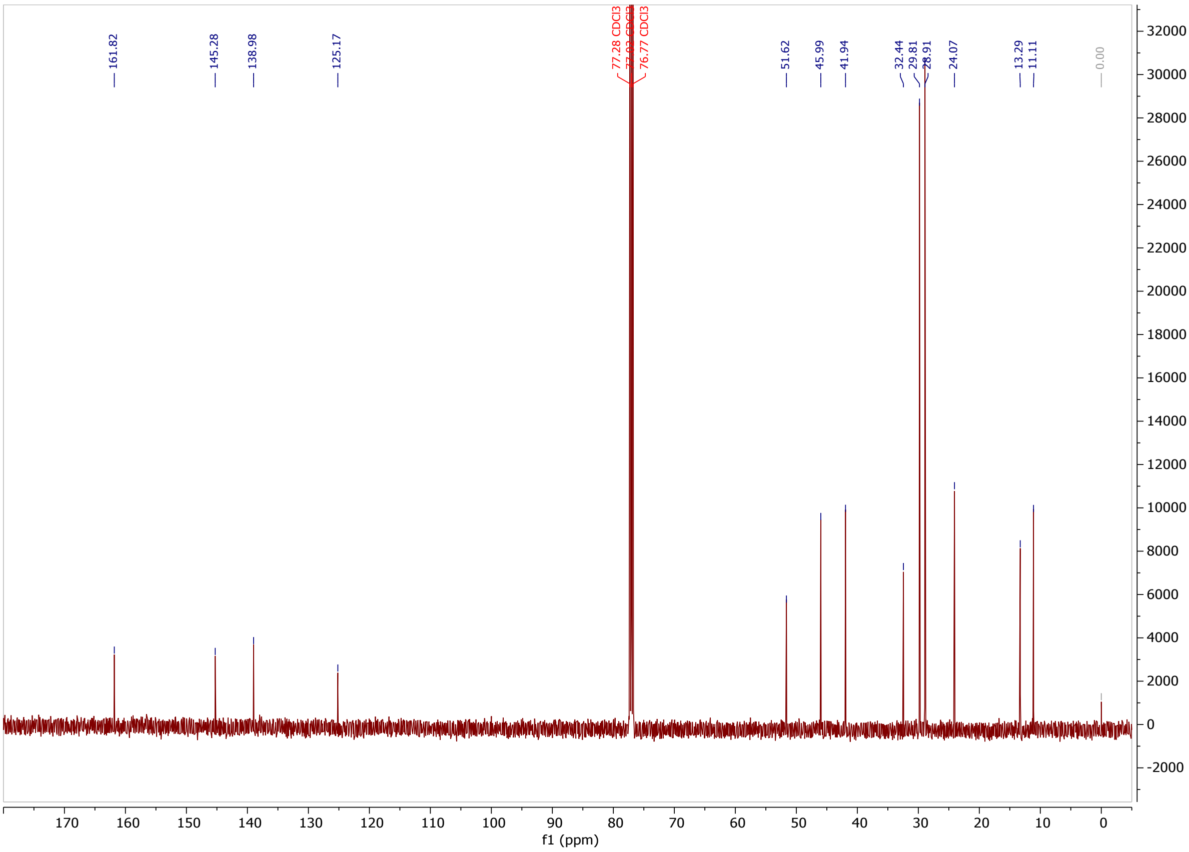

^{1}H and ^{13}C NMR spectra of compound 6u

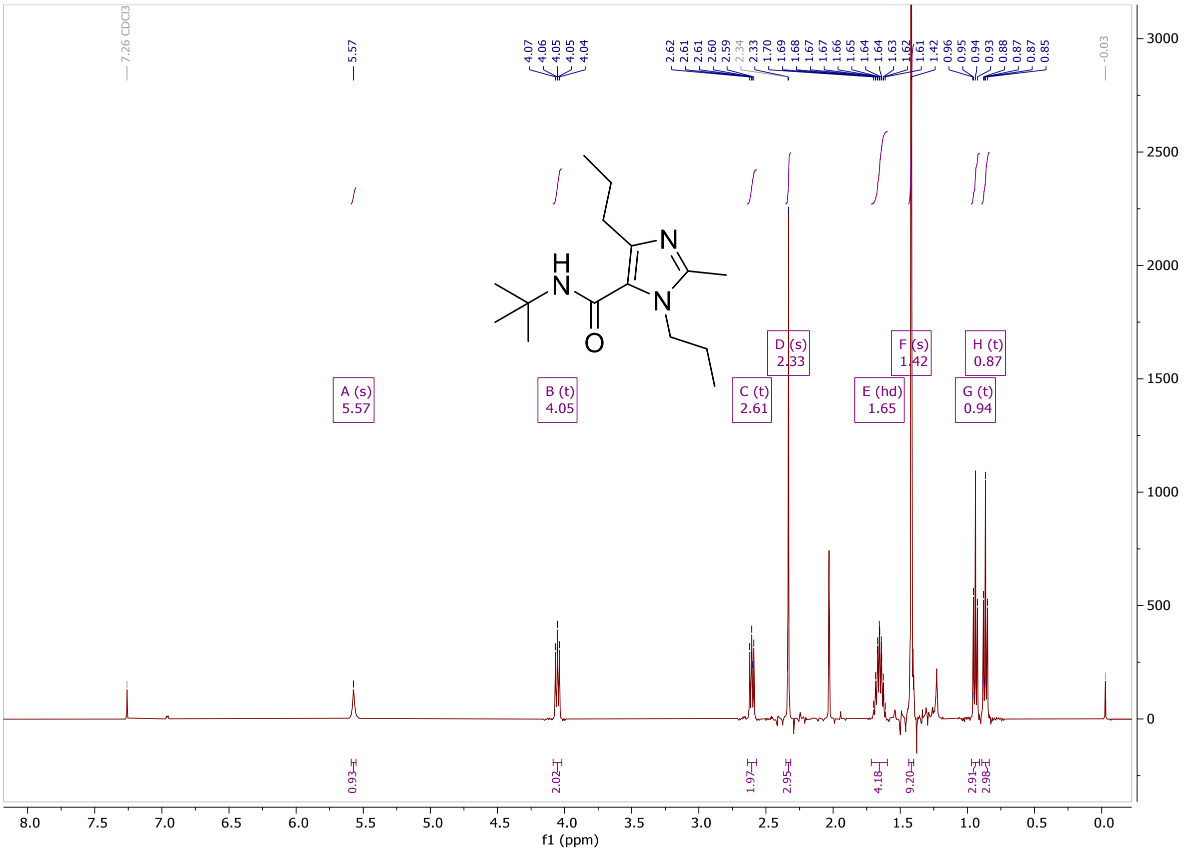

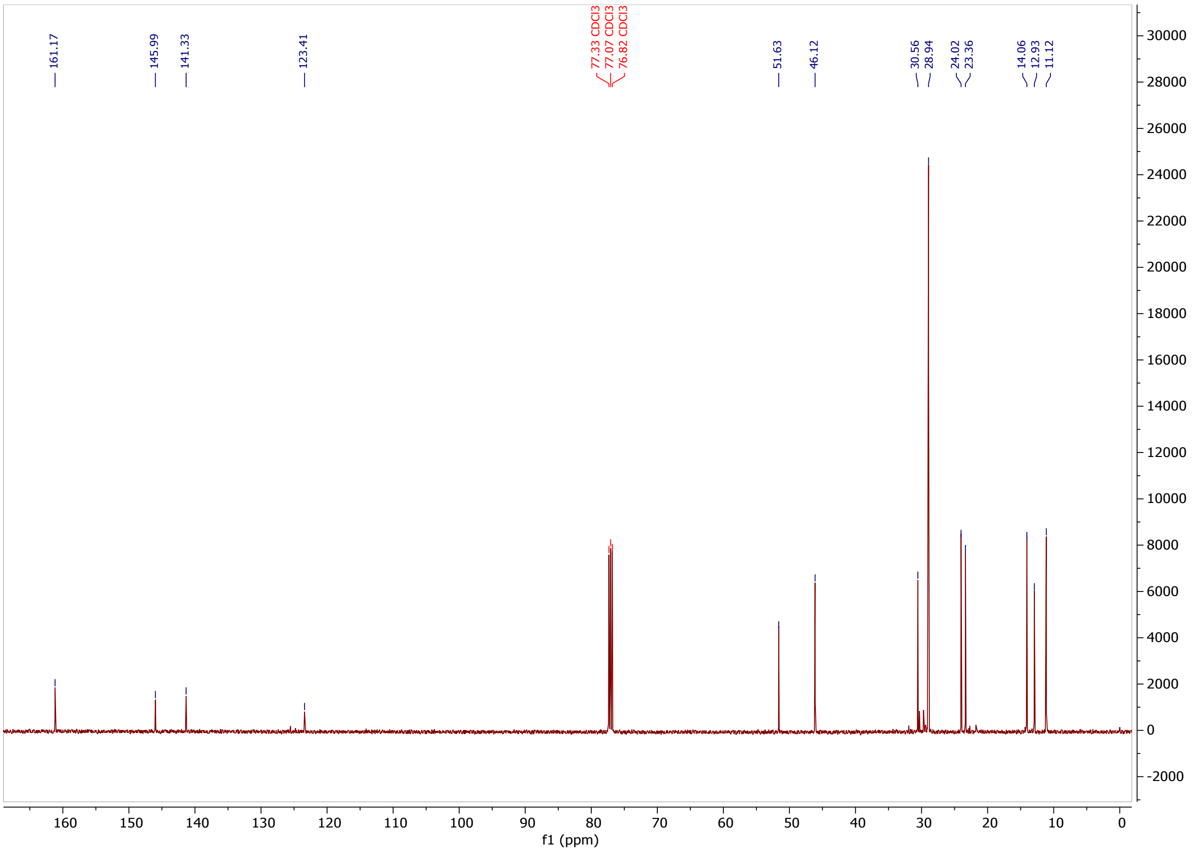

^{1}H and ^{13}C NMR spectra of compound 6v

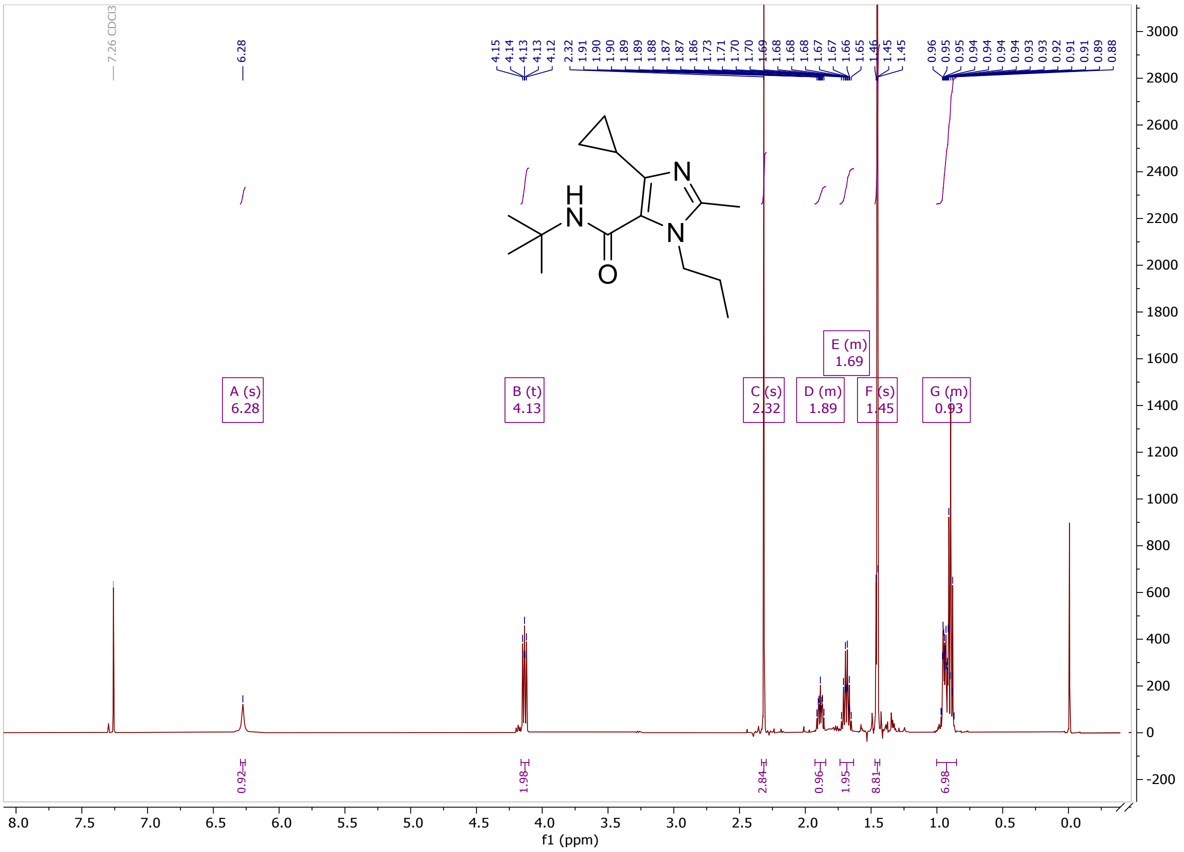

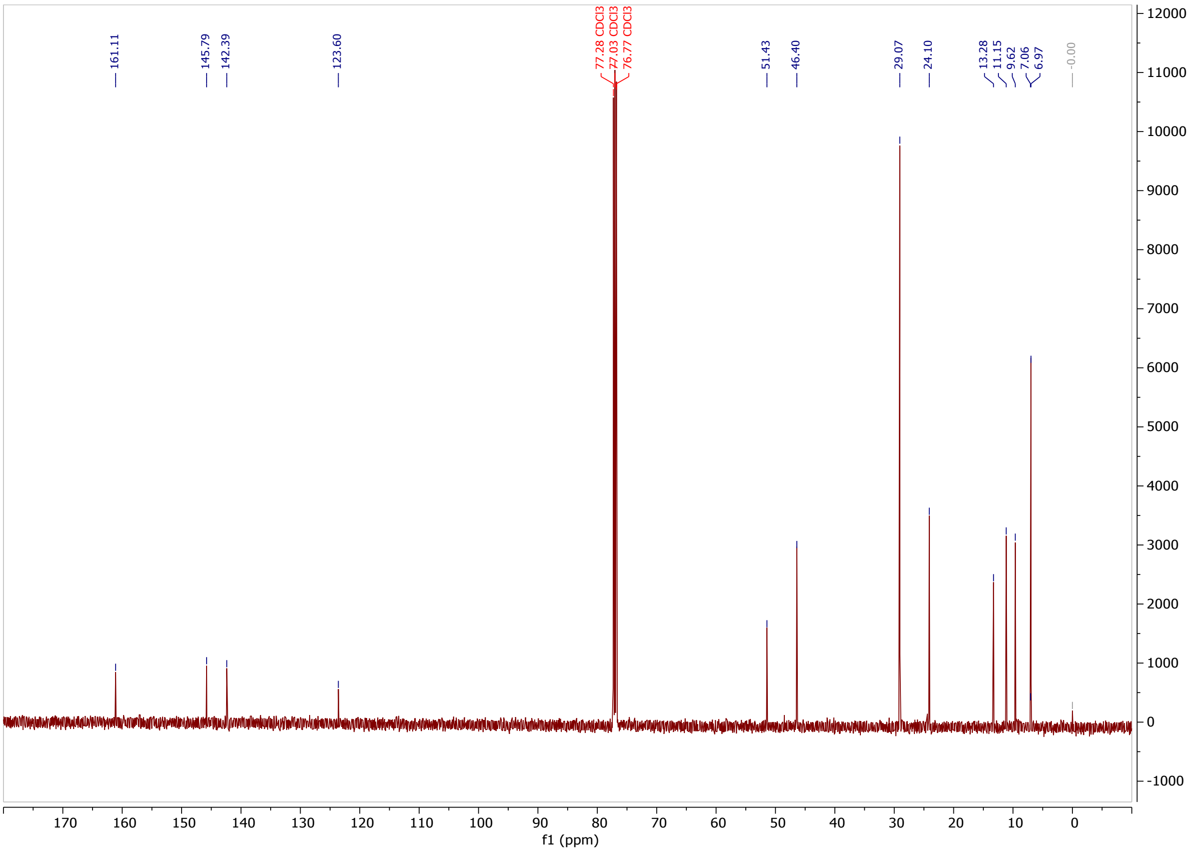

^{1}H and ^{13}C NMR spectra of compound 6w

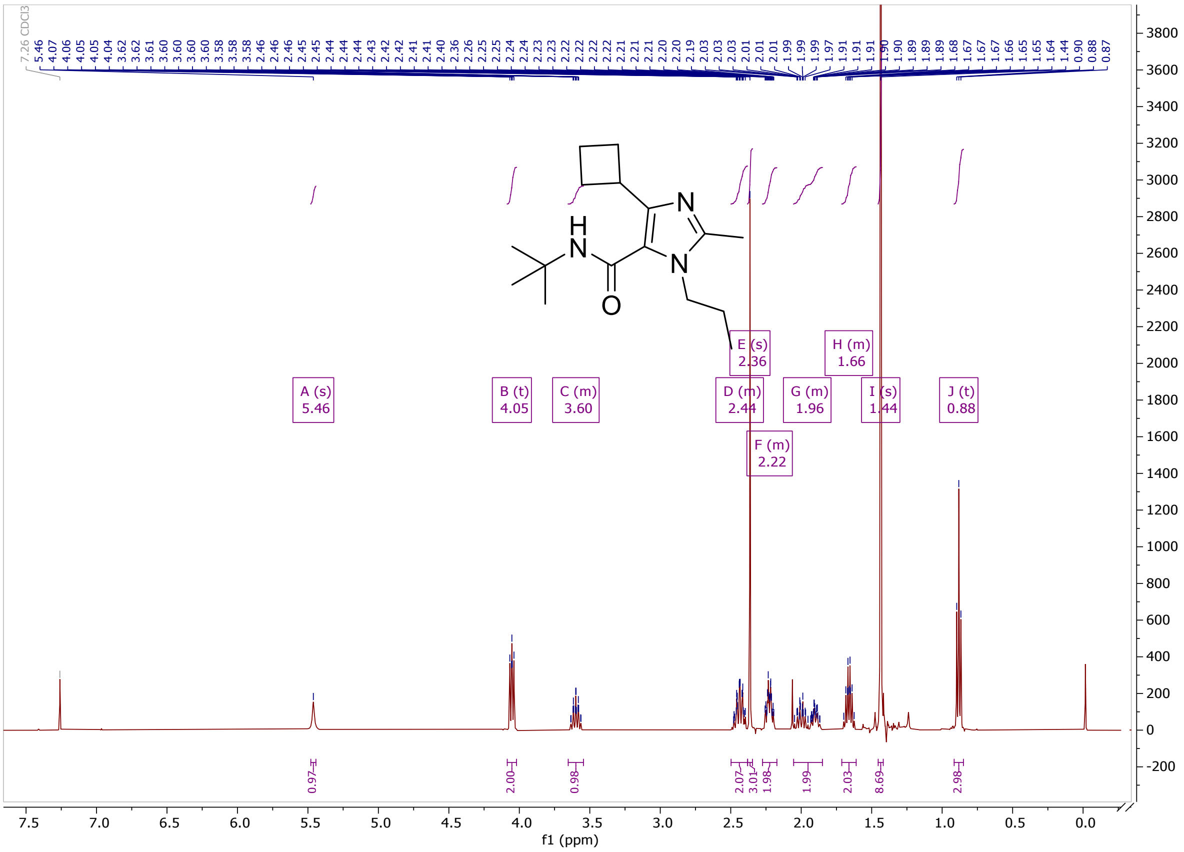

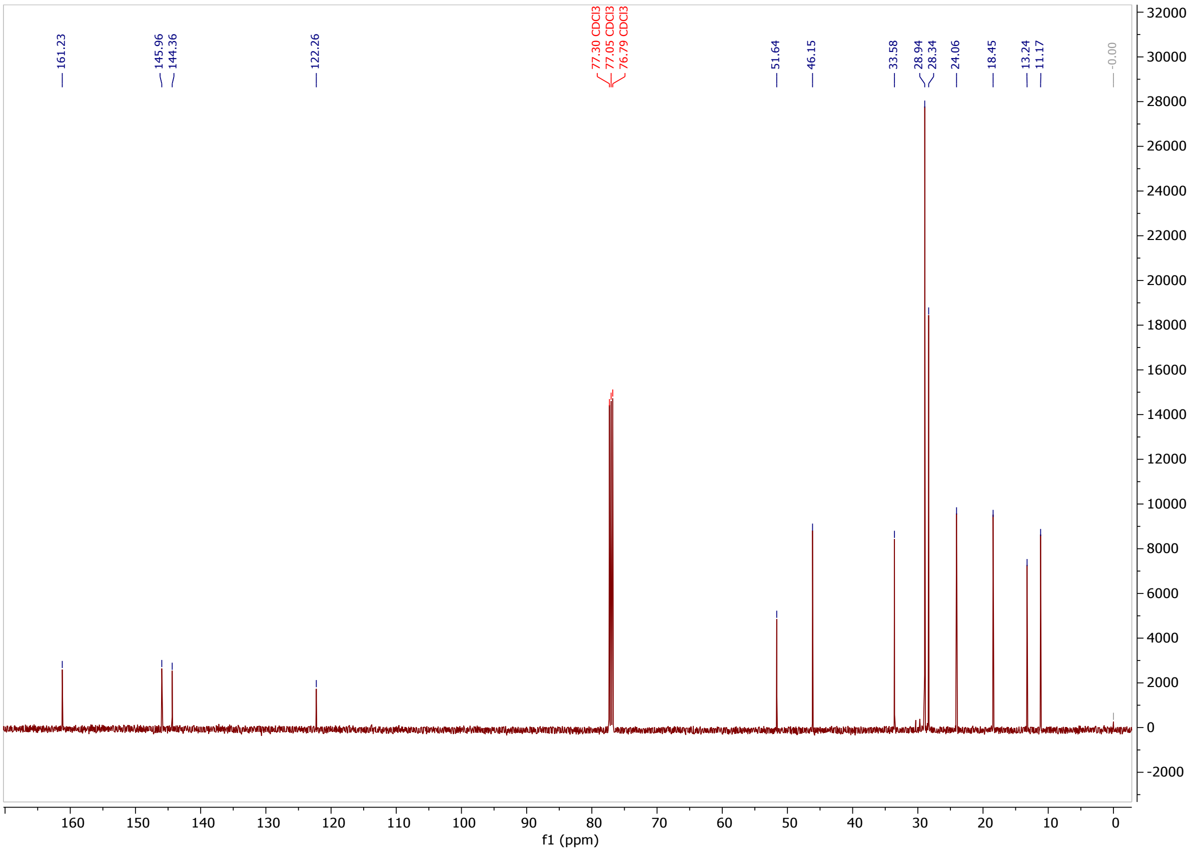

^{1}H and ^{13}C NMR spectra of compound 6x

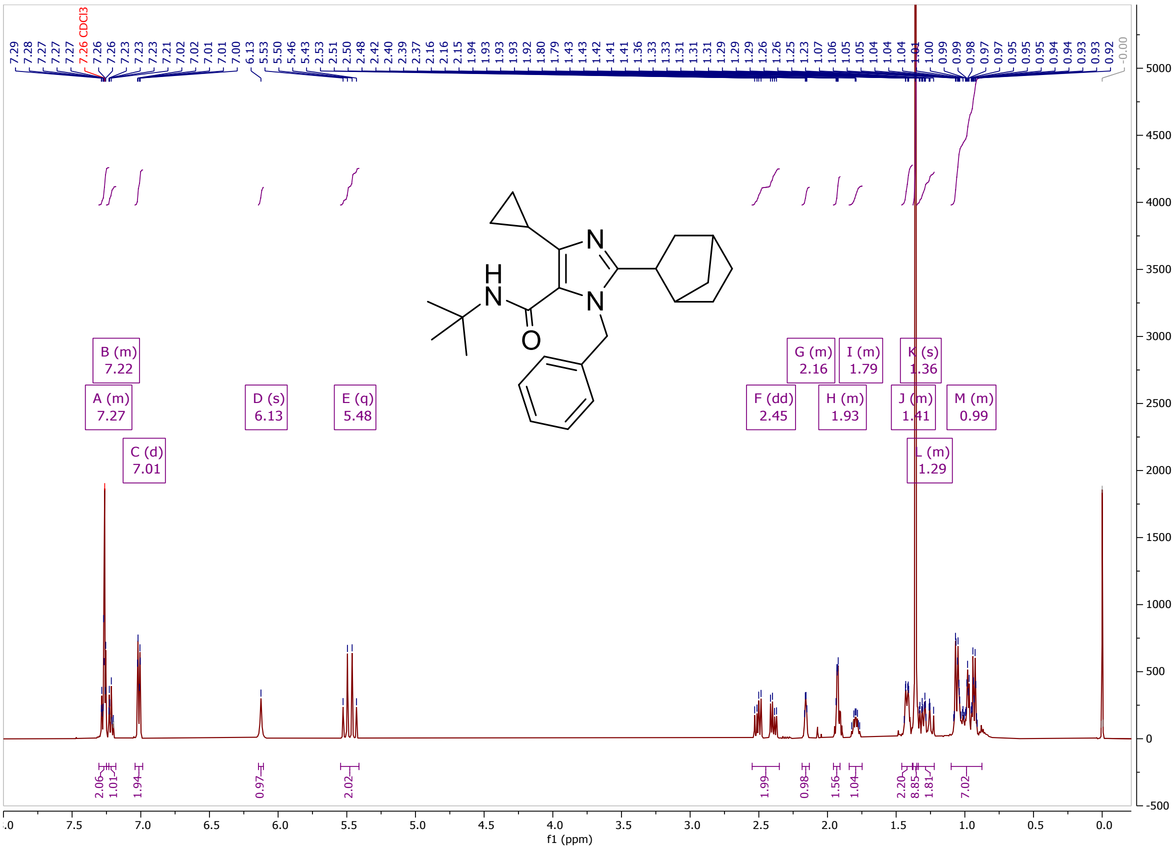

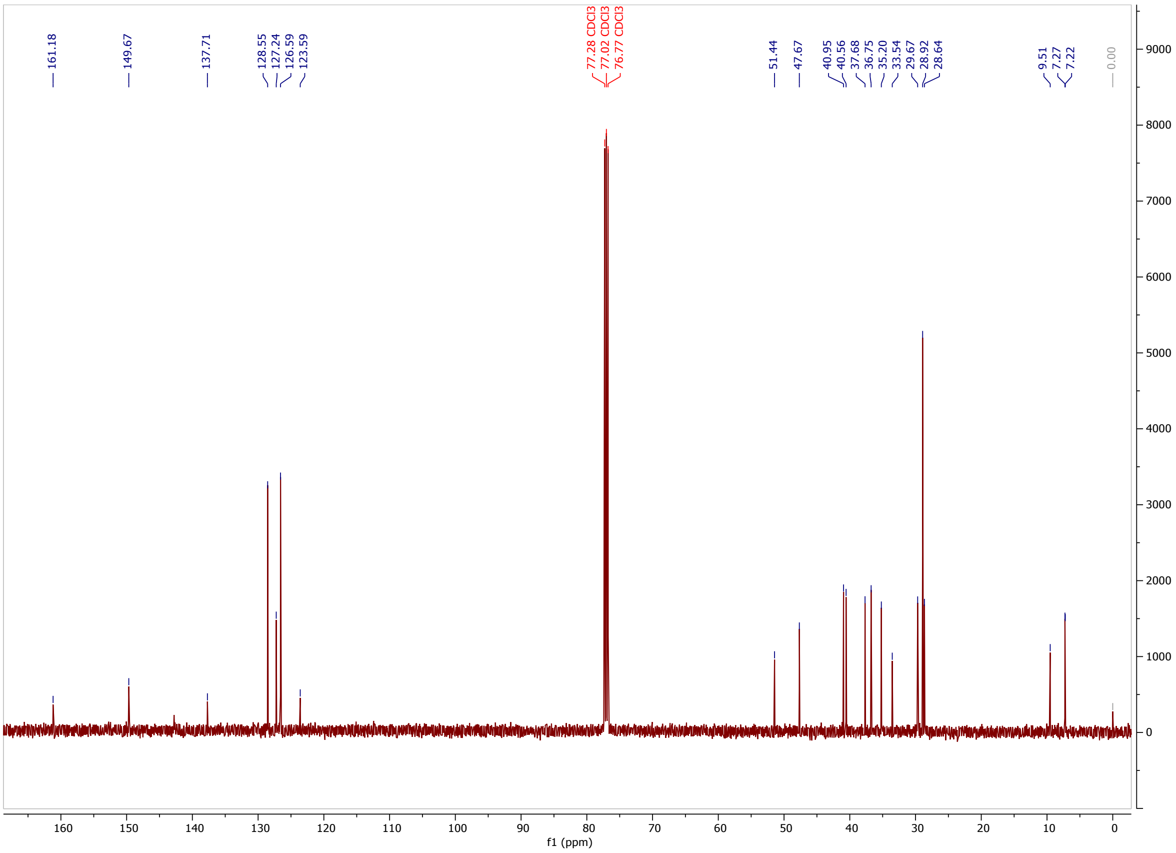

^{1}H and ^{13}C NMR spectra of compound 6y

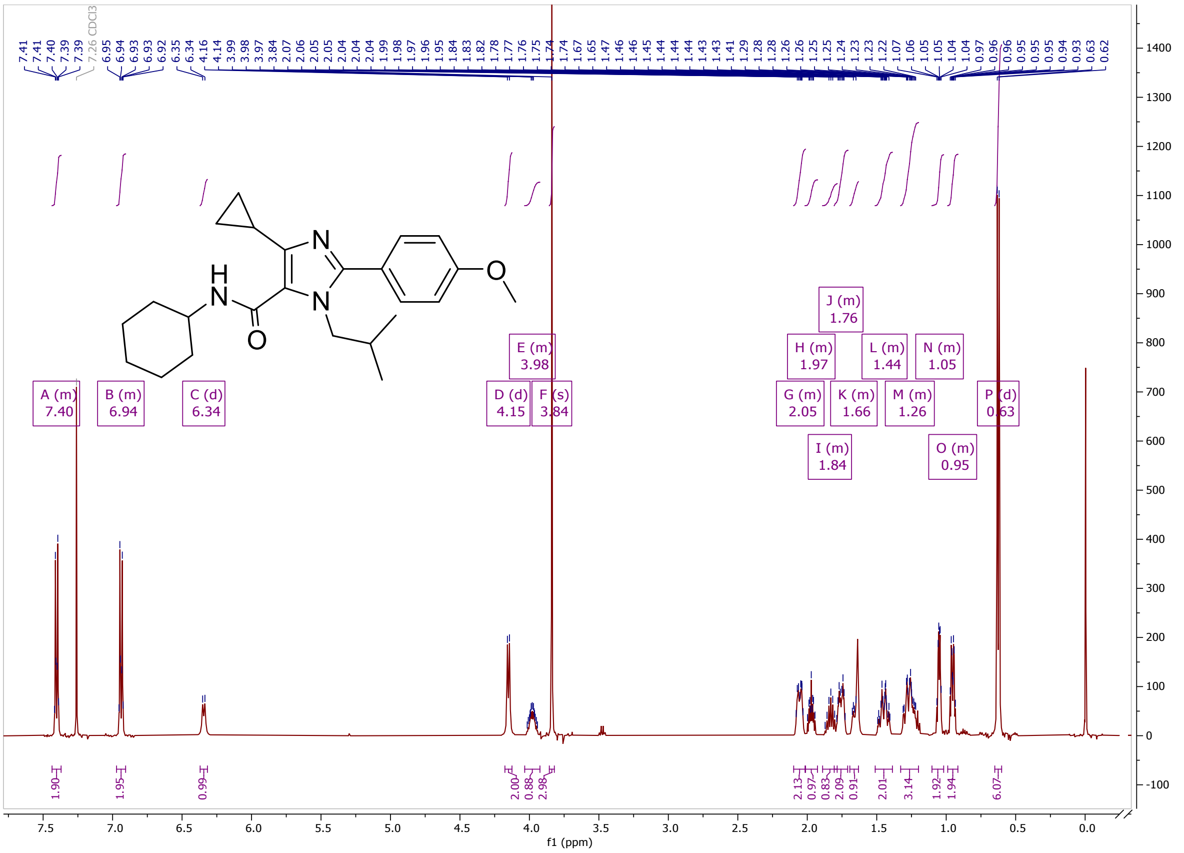

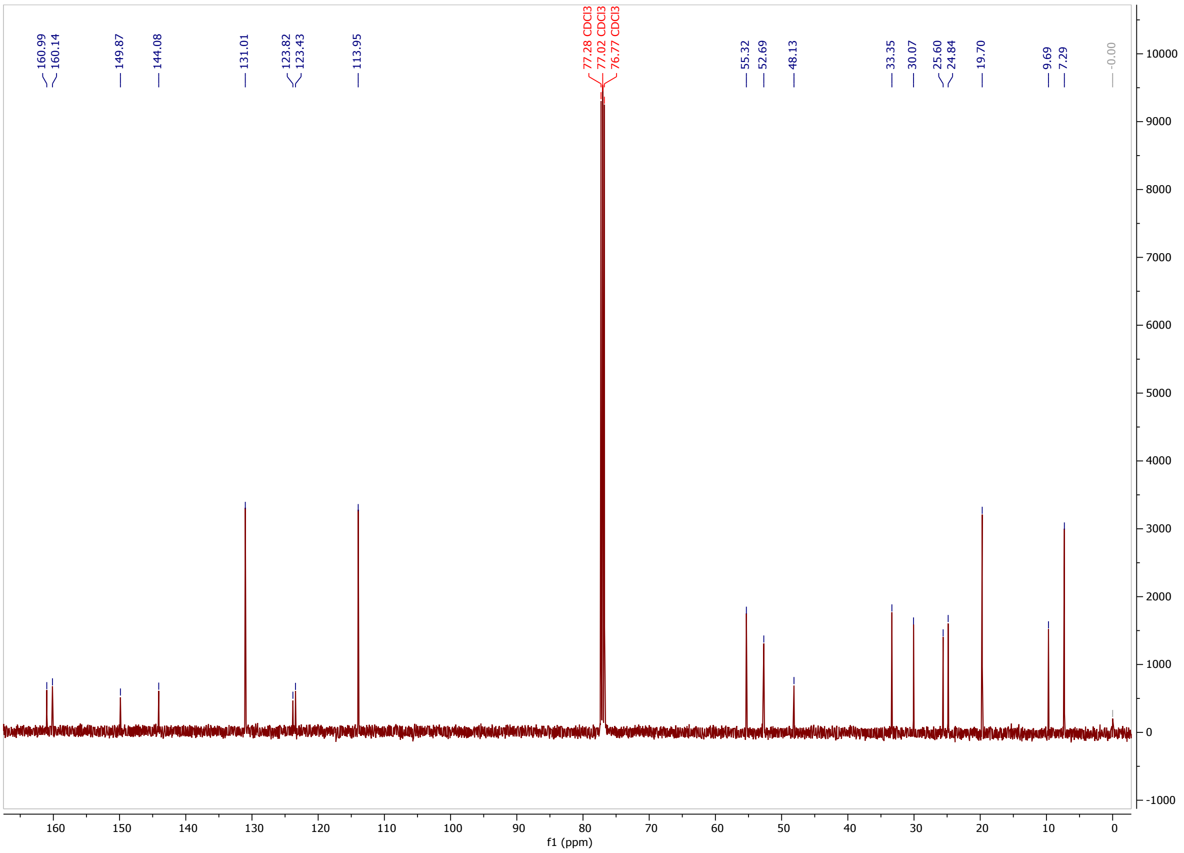

^{1}H and ^{13}C NMR spectra of compound 6z

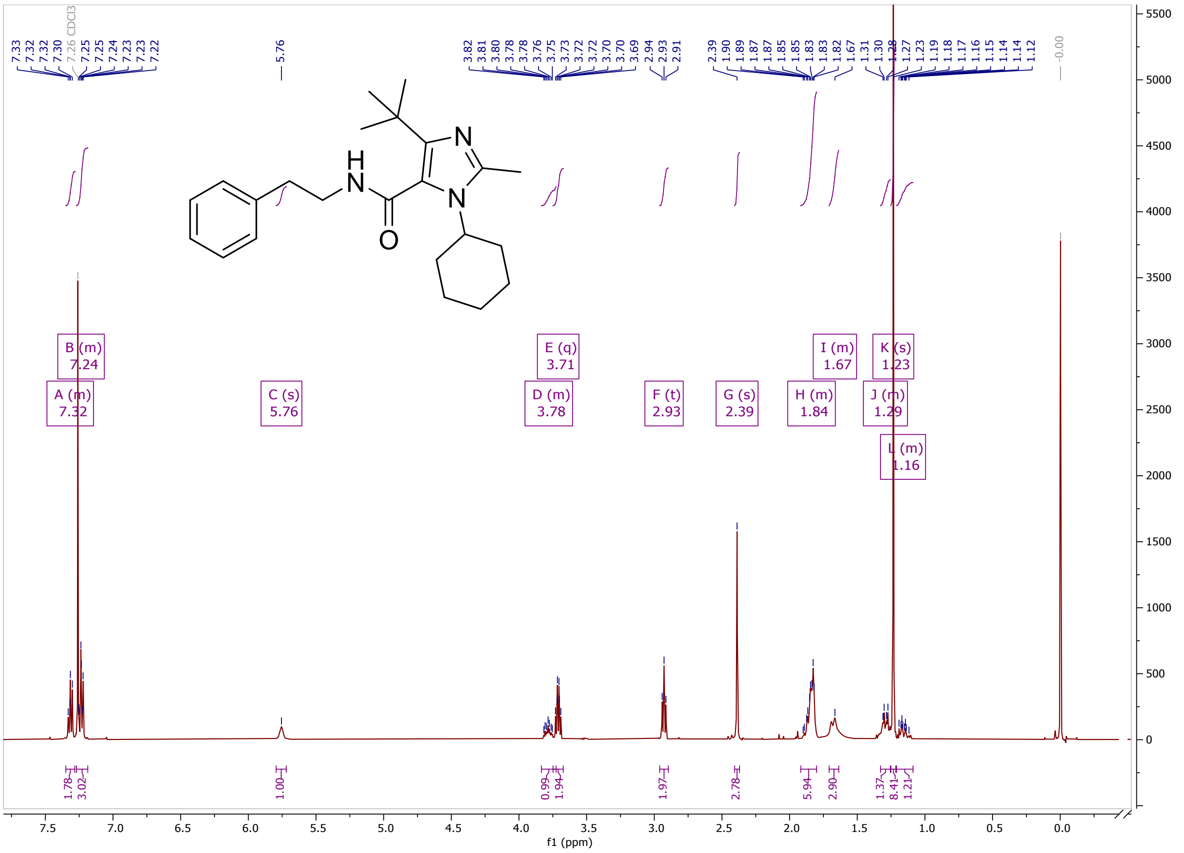

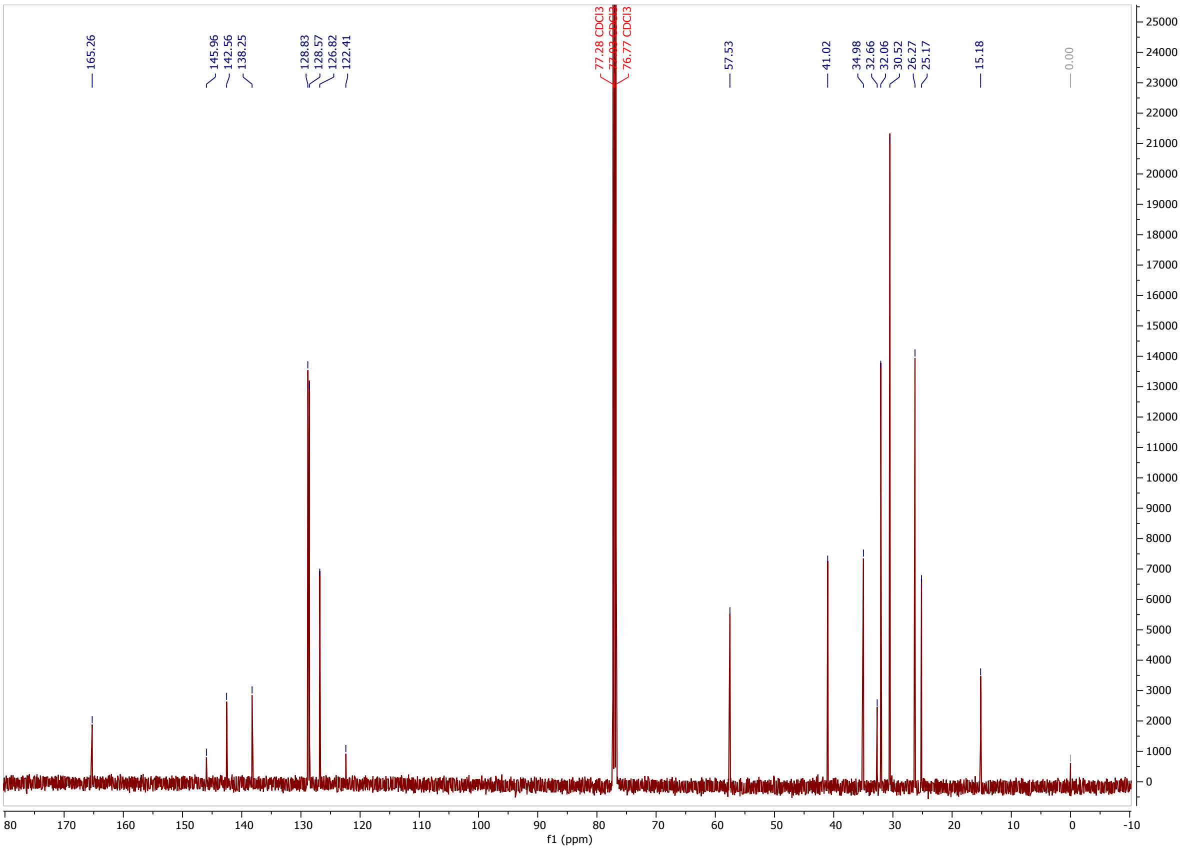

^{1}H and ^{13}C NMR spectra of compound 6aa

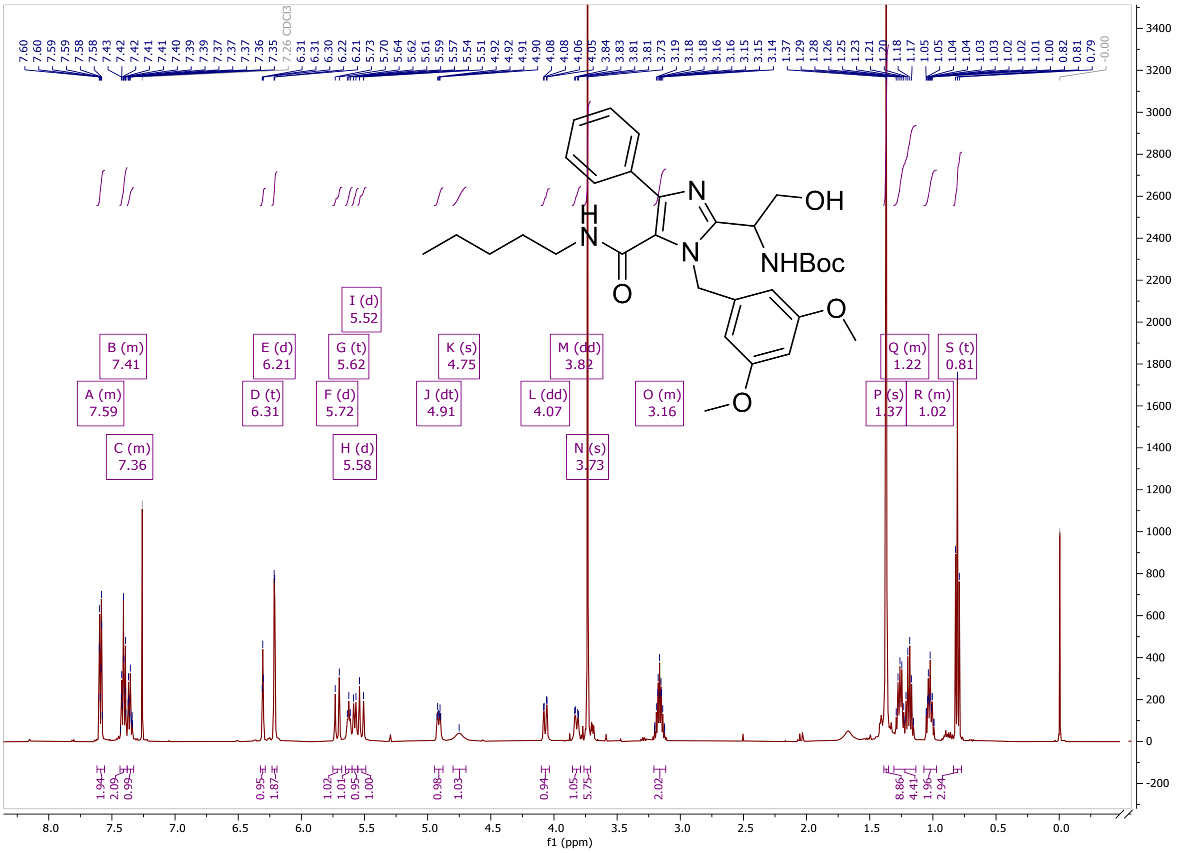

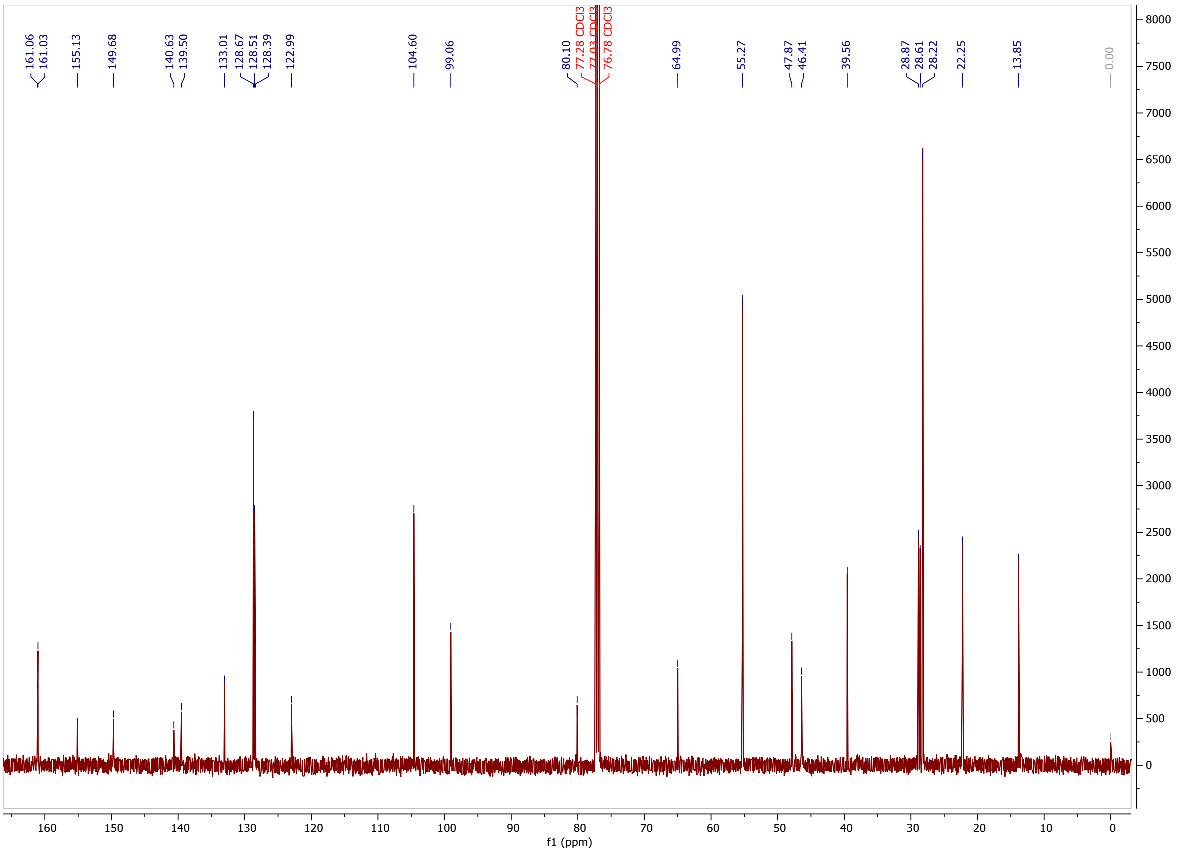

^{1}H and ^{13}C NMR spectra of compound 6ab

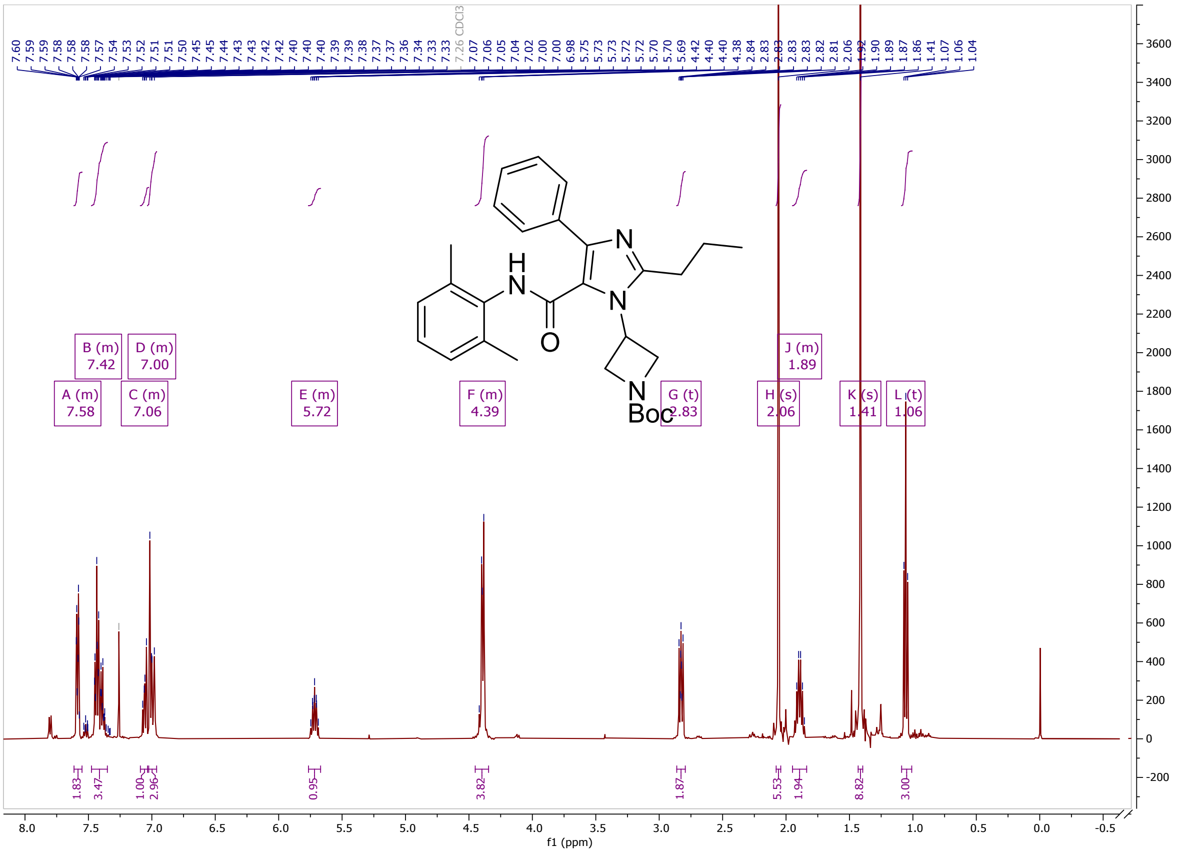

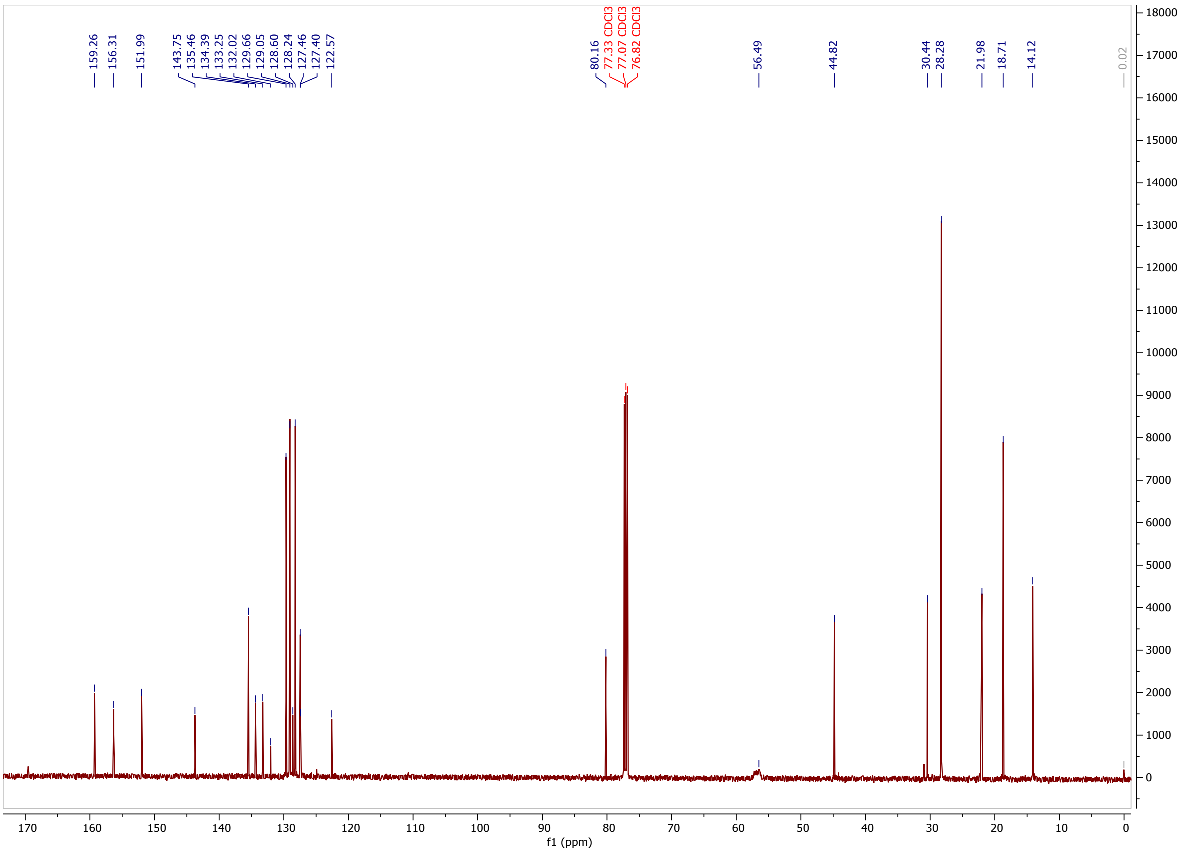

^{1}H and ^{13}C NMR spectra of compound 6ac

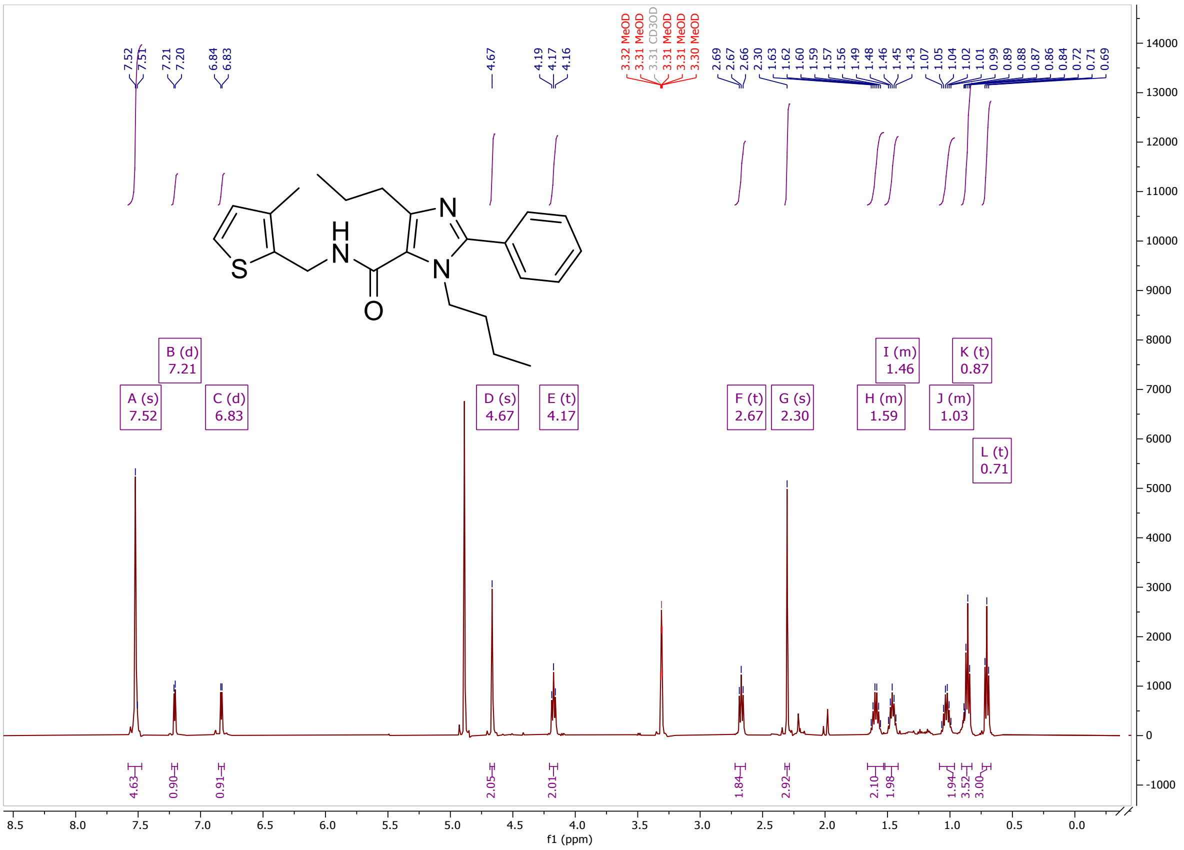

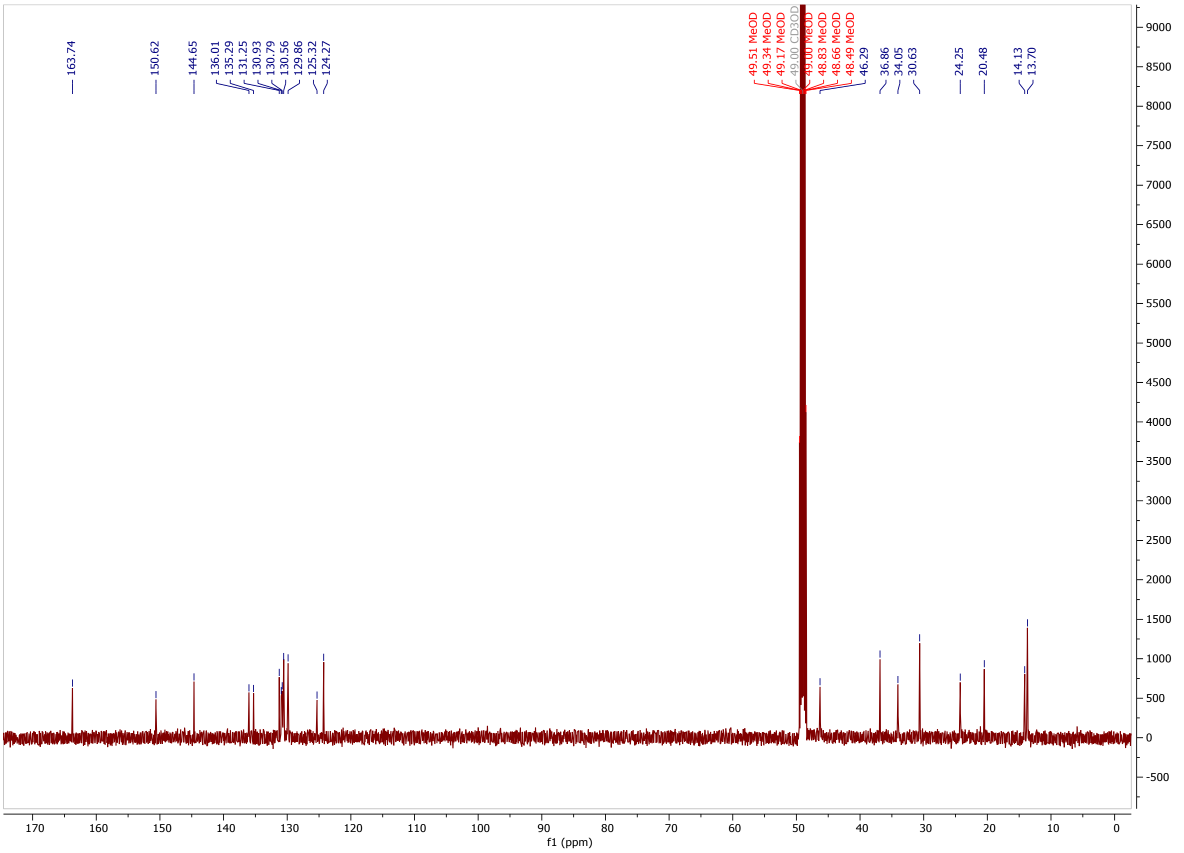

^{1}H and ^{13}C NMR spectra of compound 6ad

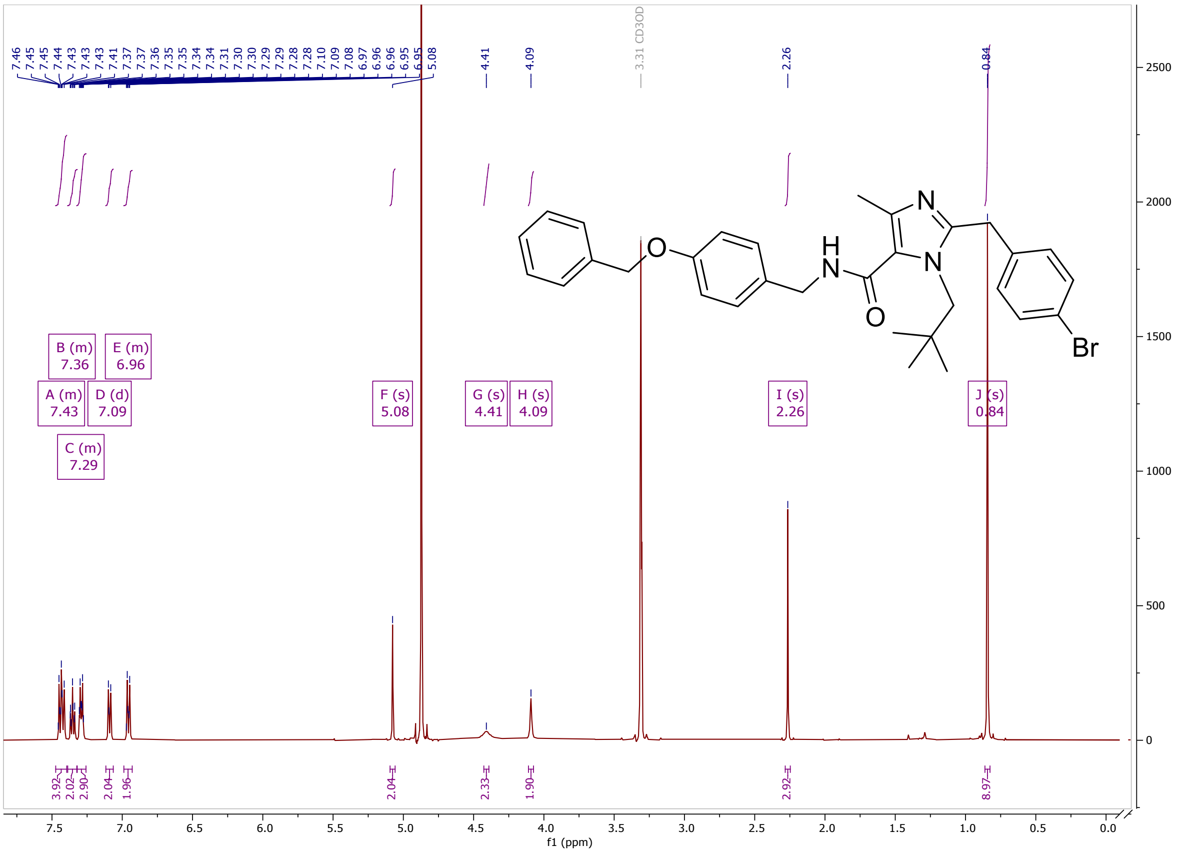

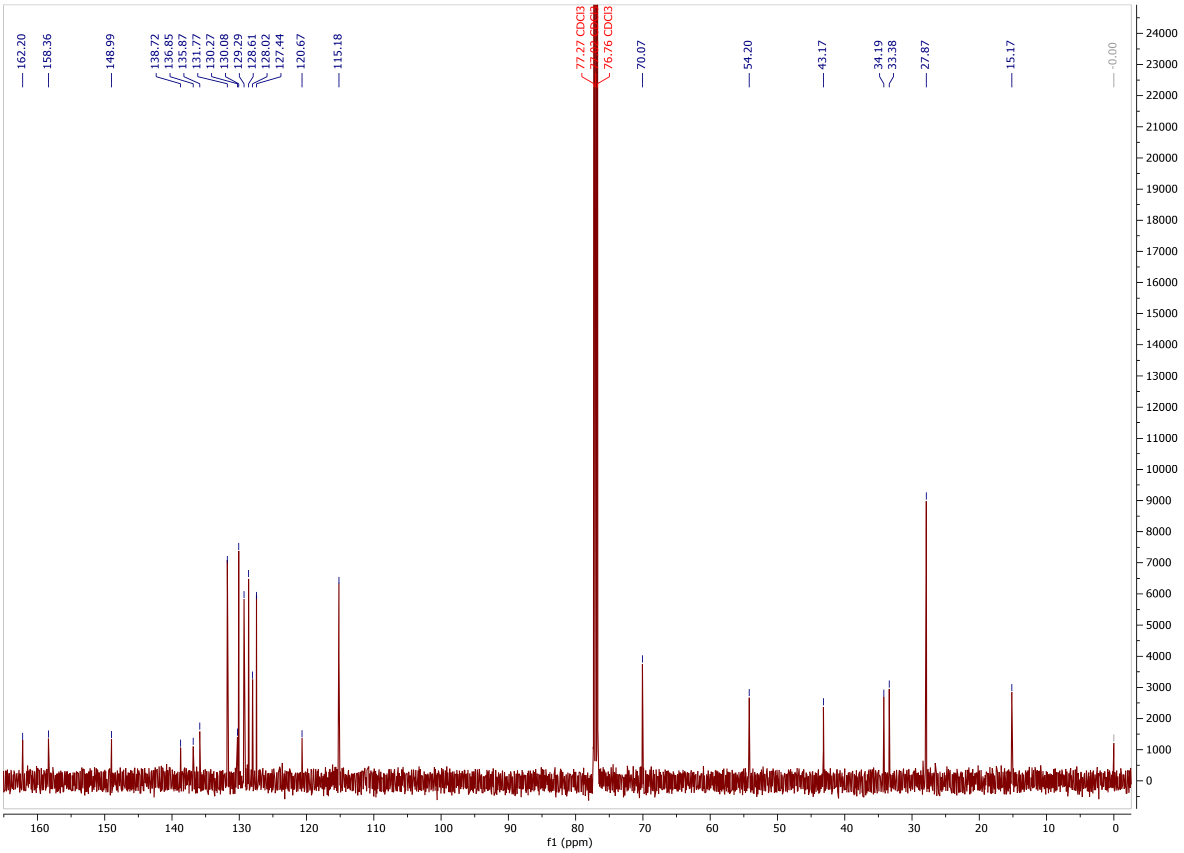

^{1}H and ^{13}C NMR spectra of compound 6ae

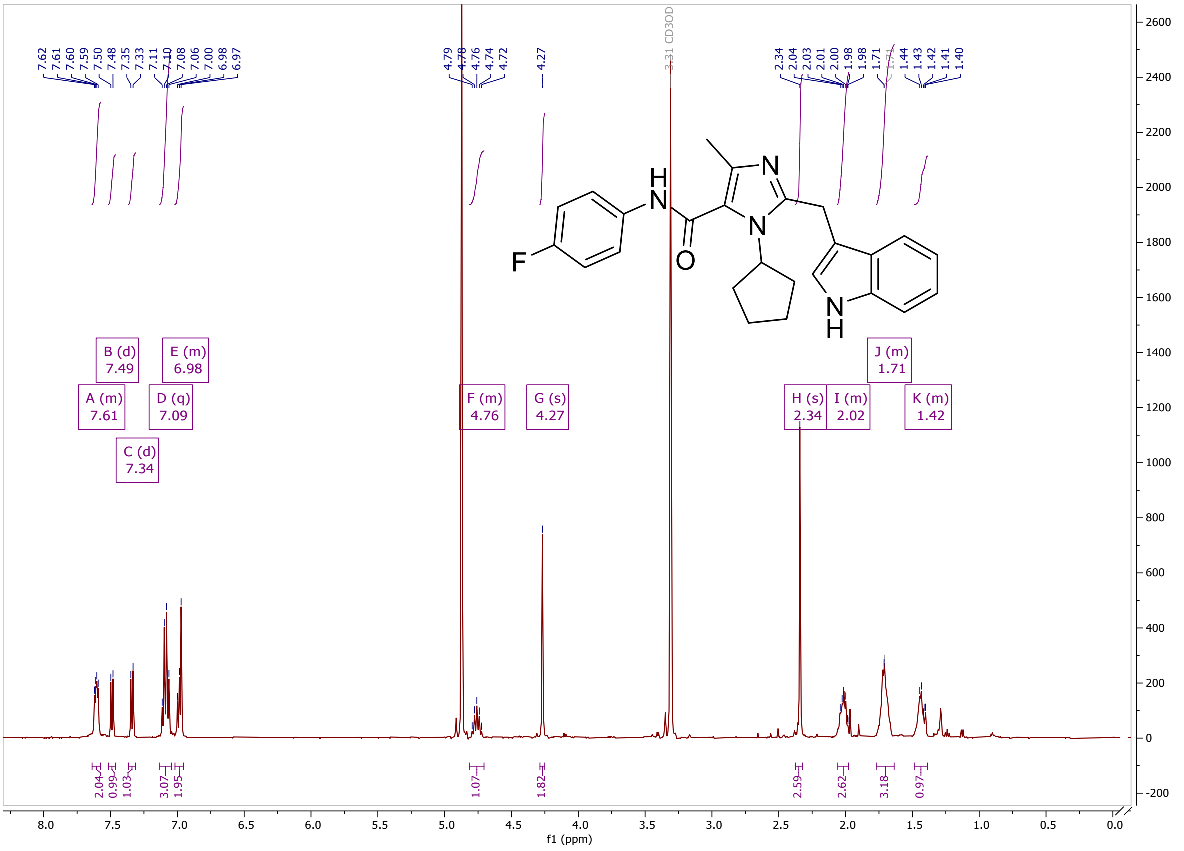

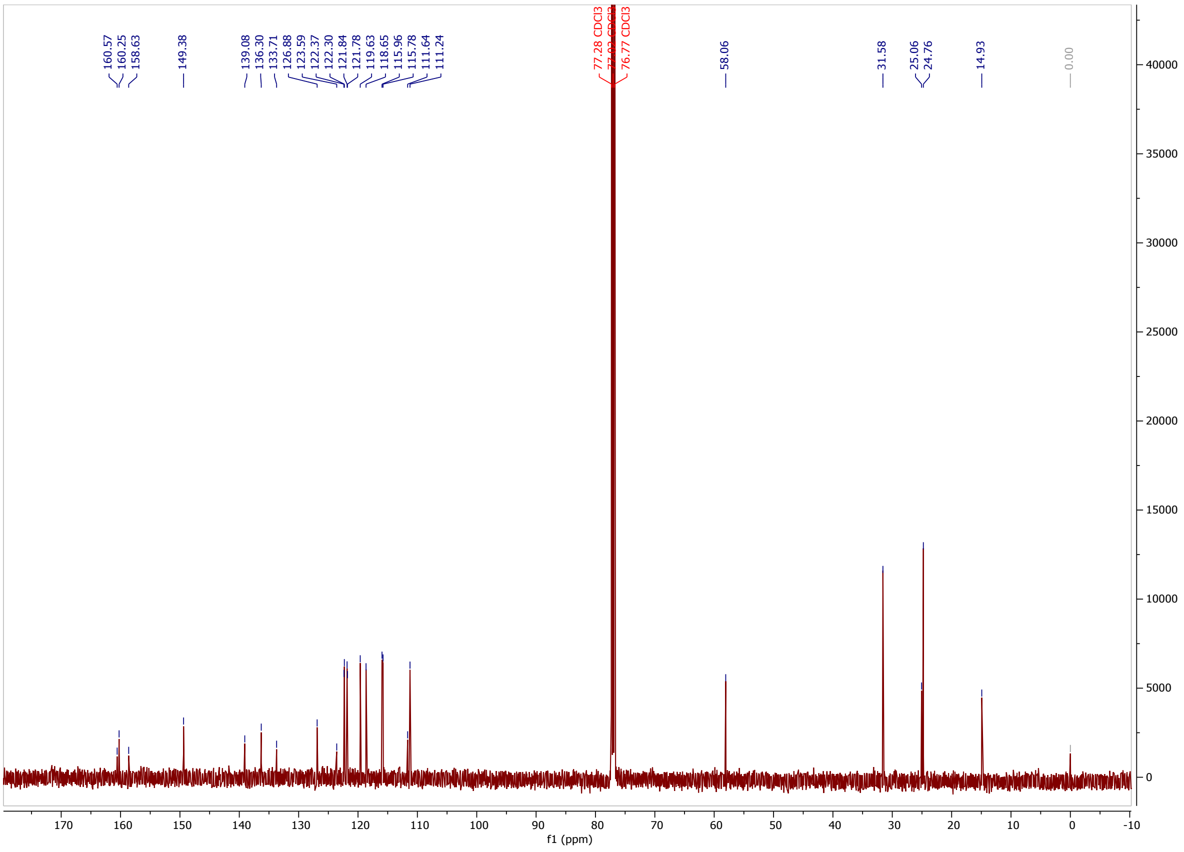

References

[S1] Rigaku-Oxford Diffraction; CrysAlisPro Oxford Diffraction Ltd, Abingdon, England V 1. 171. 36. 2. (release 27-06-2012 CN) 2006.

[S2] Burla M.C.; Caliandro R.; Carrozzini B.; Cascarano G.L.; Cuocci C.; Giacovazzo C.; Mallamo M.; Mazzone A.; Polidori G. *J. Appl. Cryst.* **2015**, *48* (1), 306–309.

[S3] Sheldrick G. M.; *ActaCryst*. **2008**, A64, 112-122.

[S4] Farrugia L.J.; *J. Appl. Cryst*. **1999**, *32*, 837-838.

[S5] Macrae C. F.; Sovago I.; Cottrell S.J.; Galek P.T.A.; McCabe P.; Pidcock E.; Platings M.; Shields G.P.; Stevens J.S.; Towler M. and Wood P.A., *J. Appl. Cryst*. **2020**, *53*, 226-235.

[S6] Grell J.; Bernstein J.; Tinhofer G.; *ActaCryst*. **1999**, B55, 1030-1043.
